# The burden of diseases and risk factors in Bangladesh, 1990–2019: a systematic analysis for the Global Burden of Disease Study 2019

**DOI:** 10.1016/S2214-109X(23)00432-1

**Published:** 2023-11-14

**Authors:** GBD 2019 Bangladesh Burden of Disease Collaborators, Sheikh Mohammed Shariful Islam, Riaz Uddin, Subasish Das, Syed Imran Ahmed, Sojib Bin Zaman, Sheikh Mohammad Alif, Md Tanvir Hossen, Malabika Sarker, George Siopis, Katherine M Livingstone, Max L Mehlman, Md. Marufur Rahman, Rahat I. Chowdhury, Md. Abdul Alim, Sohel Reza Choudhury, Syed Masud Ahmed, Ripon Kumar Adhikary, Afifa Anjum, Palash Chandra Banik, Fazle Rabbi Chowdhury, Md Omar Faruk, Rajat Das Gupta, Md Abdul Hannan, Md Nuruzzaman Haque, Syed Emdadul Haque, M Tasdik Hasan, Md Belal Hossain, Md Mahbub Hossain, Muttaquina Hossain, Sahadat Hossain, Sheikh Jamal Hossain, M Nuruzzaman Khan, Md Jobair Khan, Mohammed A Mamun, Ali H Mokdad, Mohammad Ali Moni, Christopher J L Murray, Mahfuzar Rahman, Md Mosfequr Rahman, Mosiur Rahman, Zubair Ahmed Ratan, Rezaul Karim Ripon, KM Saif-Ur-Rahman, Abu Sayeed, Md Shahjahan Siraj, Saima Sultana, Ralph Maddison, Simon I Hay, Mohsen Naghavi

## Abstract

**Background:**

Bangladesh has made substantial progress in improving socioeconomic and health indicators over the past 50 years, but data on national disease burden are scarce. We used data from the Global Burden of Diseases, Injuries, and Risk Factors Study (GBD) 2019 to estimate the burden of diseases and risk factors in Bangladesh from 1990 to 2019.

**Methods:**

For this systematic analysis, we analysed data from vital registration systems, surveys, and censuses using multistage modelling processes to estimate life expectancy at birth, mortality rate, years of life lost (YLLs), years lived with disability (YLDs), and disability-adjusted life-years (DALYs). Additionally, we compared the health status of Bangladesh with that of the other countries in the GBD south Asia region—Bhutan, India, Nepal, and Pakistan.

**Findings:**

Life expectancy at birth in Bangladesh increased from 58·2 years (95% uncertainty interval 57·1–59·2) in 1990 to 74·6 years (72·4–76·7) in 2019. Between 1990 and 2019, the age-standardised mortality rate decreased from 1509·3 (1428·6–1592·1) to 714·4 (604·9–838·2) deaths per 100 000 population. In 2019, non-communicable diseases represented 14 of the top 20 causes of death; the leading three causes were stroke, ischaemic heart disease, and chronic obstructive pulmonary disease. High blood pressure, high fasting plasma glucose, and smoking were the top three risk factors. From 1990 to 2019, the rate of all-cause DALYs decreased by 54·9% (48·8–60·4). In 2019, the leading causes of DALYs and YLLs were neonatal disorders, stroke, and ischaemic heart disease, whereas musculoskeletal disorders, depressive disorders, and low back pain were the leading causes of YLDs. Bangladesh has the lowest age-standardised rates of mortality, YLDs, and YLLs and the highest life expectancy at birth in south Asia.

**Interpretation:**

Over the past 30 years, mortality rates have reduced by more than half in Bangladesh. Bangladesh must now address the double burden of communicable and non-communicable diseases. Cost-effective, multisectoral efforts are needed to prevent and control non-communicable diseases, promote healthy lifestyles, and prevent premature mortality and disabilities.

**Funding:**

Bill & Melinda Gates Foundation.

**Translation:**

For the Bangla translation of the abstract see Supplementary Materials section.

## Introduction

Bangladesh is a lower-middle-income country in Asia and, with a population of 173 million, is the eighth most densely populated country globally, with 1328 people per km^2^.^1^ Bangladesh has one of the fastest growing economies in Asia, with a gross domestic product growth rate of 7·2% in 2022,^2^ and the country ranks second in Asia in terms of gender parity (World Economic Forum), with women as head of state, leader of the opposition party, and speaker of the parliament. Despite attaining notable progress on most health indicators for the Sustainable Development Goals, Bangladesh has a high prevalence of diarrhoea, tuberculosis, dengue, and other infectious diseases.^3^, ^4^, ^5^, ^6^ Although the risk factors for infectious diseases, such as insufficient sanitation, are still a threat, the increasing pattern of sedentary lifestyles and unhealthy diets among the population of Bangladesh is adding to the burden of non-communicable diseases. This transformation has disrupted the already fragile health service delivery system in Bangladesh.

Bangladesh has a pluralistic health-care system that involves multiple stakeholders—including government, private sectors, donor agencies, and non-governmental organisations—implementing targeted programmes for immunisation, maternal and child care, and family planning, among others.^7^ The Ministry of Health and Family Welfare provides primary, secondary, and tertiary care services at the community and national levels, whereas non-governmental organisations support community health workers in providing priority services to households. Private hospitals in Bangladesh provide modern facilities and specialised care, but are expensive, therefore limiting access for individuals with low incomes. In 1997, the Ministry of Health and Family Welfare embarked on a sector-wide approach to align funding and technical support around national priorities and develop partner coordination. The Bangladesh Government's Health, Population, and Nutrition Sector Development Program 2017–2022 has contributed to reduced mortality, morbidity, and malnutrition; improved immunisation; and reduced neonatal deaths and infectious diseases. Bangladesh is also one of the first nations in Asia to implement digital health strategies, including an electronic immunisation register, text messaging for patient communication, mobile health applications, electronic prescriptions, and remote diagnostic services.^8^, ^9^, ^10^, ^11^, ^12^ However, the country had a low overall score of 52 out of 100 on the 2017 Healthcare Access and Quality Index.^13^ Bangladesh faces several health system challenges, including poor government coordination, a shortage of skilled health workers, a low health budget, high out-of-pocket health expenditure, and inequitable access to health services.^14^

**Research in context**

**Evidence before this study**

We searched PubMed, Google Scholar, and governmental websites for estimates of the burden of diseases in Bangladesh using the search terms “burden”, “cause of death”, “death”, “prevalence”, “epidemiology”, “morbidity”, “mortality”, “trends”, “disability”, “DALY”, “Bangladesh”, “chronic diseases”, “NCD”, “noncommunicable diseases”, “communicable diseases”, “cardiovascular diseases”, “chronic obstructive pulmonary disease”, and “cancer”, from database inception to July 4, 2023 and without language or other publication restrictions. The 2013 Series *Bangladesh: Innovation for Universal Health Coverage*, published in *The Lancet*, reported on Bangladesh's health achievements and challenges. This six-part Series presented case studies on Bangladesh's exceptional health advances at low cost. Additionally, several national and subnational studies have reported the burden of different diseases and risk factors in Bangladesh at different points during the past three decades. However, to our knowledge, there are no studies on the burden and trends of diseases in Bangladesh that enable global comparisons. Bangladesh has experienced demographic and epidemiological transitions over the past three decades. Although the country already has a high prevalence of communicable diseases, the increasing pattern of sedentary lifestyles and energy-dense but poorly nutritious diets among the young and middle-aged population of Bangladesh is contributing to the pervasive burden of non-communicable diseases. Such epidemiological transformations might further weaken the already fragile health service delivery system. To prepare the health system, understanding the prevalence and trends of the burden of diseases and their risk factors is necessary.

**Added value of this study**

To our knowledge, this study is the first to systematically analyse the trends in the burden of diseases and risk factors from 1990 to 2019 in Bangladesh. We used standardised and globally comparable metrics, adjusting for variability in data sources and removing potential biases, to generate results comparable with those of other countries. We analysed 286 causes of death, 369 diseases and injuries, and 87 behavioural, metabolic, and environmental and occupational risks among the Bangladeshi population with a comprehensive analysis of changes in population health from 1990 to 2000, 2000 to 2010, and 2010 to 2019. Our results provide important information for assessing health progress and for attaining the goals and targets set by the Health, Population, and Nutrition Sector Development Program of the Bangladesh Government and the UN's Sustainable Development Goals. Additionally, we compared the performance of Bangladesh's health system with that of health systems in other countries of the Global Burden of Diseases, Injuries, and Risk Factors Study south Asia region to identify crucial areas of improvement and challenges faced by Bangladesh's health sector.

**Implications of all the available evidence**

Although the all-cause mortality rate has reduced over the past three decades in Bangladesh, the top-ranked causes of death now include stroke, ischaemic heart disease, cancer, and diabetes. Road-traffic injuries, musculoskeletal disorders, depressive disorders, low back pain, headache disorders, age-related hearing loss, dietary iron deficiency, blindness and vision loss, and gynaecological disorders are emerging issues on which public health interventions, policies, and programmes should have a strong focus. Both communicable and non-communicable disease risk factors contribute to mortality and morbidity in the Bangladeshi population. Therefore, a multisectoral, coordinated approach targeting prevention, care, and rehabilitation is needed to ensure the sustainability of the country's health-care system.

Bangladesh has made substantial health advances over the past 30 years despite spending less on health care than other countries in south Asia.^7^ Understanding the prevalence and trends of diseases and their risk factors is necessary. Several studies have analysed the burden of specific diseases and risks in Bangladesh.^15^, ^16^, ^17^, ^18^, ^19^, ^20^, ^21^, ^22^, ^23^ However, national-level data over time on the overall burden of diseases and risk factors in Bangladesh are scarce. Furthermore, studies comparing the health-care performance of countries in south Asia using standardised data are not available. The Global Burden of Diseases, Injuries, and Risk Factors Study (GBD) 2019 produced globally comparable estimates of disease burden and risk factors in 204 countries and territories over time. We analysed the burden and trends of diseases and their risk factors in Bangladesh from 1990 to 2019 and compared the results with those from the other countries in the GBD south Asia region—hereafter referred to as south Asia—using the comprehensive GBD methodology. Our study provides information for measuring progress and attaining goals set by the government of Bangladesh and other stakeholders.

## Methods

### Data sources

We accessed country-specific burden of disease estimates for Bangladesh from GBD 2019 using the Global Health Data Exchange, the GBD Results tool, and the GBD Compare tool. In this study, data were obtained from a total of 688 data sources: scientific literature (n=380), survey data (n=132), epidemiological surveillance data (n=45), reports (n=42), administrative data (n=33), estimate data (n=14), vital registration systems (n=10), environmental monitoring data (n=10), event data (n=7), census data (n=6), demographic surveillance data (n=5), registries (n=3), and intervention studies (n=1). All data sources are described in appendix 2 (p 47). This study follows the Guidelines for Accurate and Transparent Health Estimates Reporting.

### Estimates

The data sources listed in the previous subsection were used to examine total fertility rate, age-standardised and all-cause mortality, and cause-specific mortality using GBD methods described previously.^24^ GBD uses a hierarchical list with four levels of causes of death and disease. At Level 1 there are three cause groups: communicable, maternal, neonatal, and nutritional (CMNN) diseases; non-communicable diseases; and injuries. These Level 1 groups are subdivided at Level 2 into 22 cause groups; Levels 3 and 4 further disaggregate the cause groups and contain the finest level of detail for causes captured in GBD 2019. We analysed a total of 369 fatal and non-fatal causes in Bangladesh. Additionally, we estimated mortality and disability-adjusted life-years (DALYs) attributed to 87 risk factors, categorised as three Level 1 groups (behavioural risks, environmental and occupational risks, and metabolic risks), 20 Level 2 groups, 52 Level 3 groups, and 69 Level 4 groups.^25^, ^26^ Unless otherwise specified, we present Level 3 causes of death and disease and Level 4 risk factors.

The Cause of Death Ensemble model (CODEm) was used to estimate cause-specific mortality for each combination of sex, age, location, and year. We used a Bayesian meta-regression method (Disease Modelling Meta-Regression; DisMod-MR 2.1)^24^ to generate most of the prevalence estimates for each combination of sex, age, location, and year. The DisMod-MR tool evaluated and pooled all available data, adjusted data for systematic biases, and produced estimates by world region with uncertainty intervals (UIs) through the use of Bayesian statistical methods. Subsequently, years lived with disability (YLDs), representing the non-fatal burden, were calculated using prevalence estimates and a corresponding disability weight based on disease severity, exclusivity, and comorbidity. Years of life lost (YLLs), a measure of premature death, were calculated by multiplying the sum of each death within each age group by the normative standard reference life expectancy for each respective age group.^24^ DALYs were calculated as the sum of YLLs and YLDs; 1 DALY represents 1 lost year of healthy life. Health-adjusted life expectancy (HALE), defined as the number of years spent in full health, provides a single measure of average population health and was computed using YLDs and life tables.^27^ Life expectancy at birth and for specific age groups was calculated using the world population age standard.^24^ Associations between potential risk factors and disease burden were evaluated using the GBD comparative risk assessment framework, in which estimates of theoretical minimum risk exposure level,^24^ risk exposure, relative disease risk, and attributable burden are generated for 87 risk factors (appendix 2 p 11). Age-standardised rates of all-cause mortality were estimated using multiple data types, including vital registration systems, surveys, and censuses, and total fertility rate was estimated through a systematic synthesis of all data available for all GBD locations, using the age-specific fertility pattern from World Population Prospects.^24^

### Comparisons

We compared estimates for Bangladesh with estimates for the four other countries in the GBD south Asia region (Bhutan, India, Nepal, and Pakistan), which have similar socioeconomic profiles to that of Bangladesh. We compared age-standardised rates of all-cause mortality, YLLs, and YLDs, as well as life expectancy at birth and HALE, and ranked the five countries on the basis of these metrics between 1990 and 2019. Unless otherwise specified, we report age-standardised rates per 100 000 population, with each point estimate presented with 95% UIs generated using a Monte Carlo approach.^24^

### Role of the funding source

The funder of the study had no role in study design, data collection, data analysis, data interpretation, or writing of the report.

## Results

The total fertility rate among women of reproductive age in Bangladesh was 4·4 (95% UI 4·3–4·5) in 1990 and decreased consistently over the subsequent years to 1·8 (1·6–2·0) in 2019. In 1990, the life expectancy at birth was 58·2 years (57·1–59·2), which by 2019 had increased to 74·6 years (72·4–76·7). Bangladesh also achieved a substantial improvement in HALE between 1990 (50·7 years [48·3–52·8]) and 2019 (64·5 years [61·3–67·4]; appendix 2 p 43). The total number of people with non-communicable diseases increased from 95·5 million in 1990 to 145·0 million in 2019 (all-age annual rate of change in prevalence 0·05%); those with CMNN diseases increased from 88·6 million to 104·9 million (−0·18%) and those with injuries increased from 13·1 million to 28·9 million (0·52%) during the same period (appendix 2 p 33).

**Figure 1:**
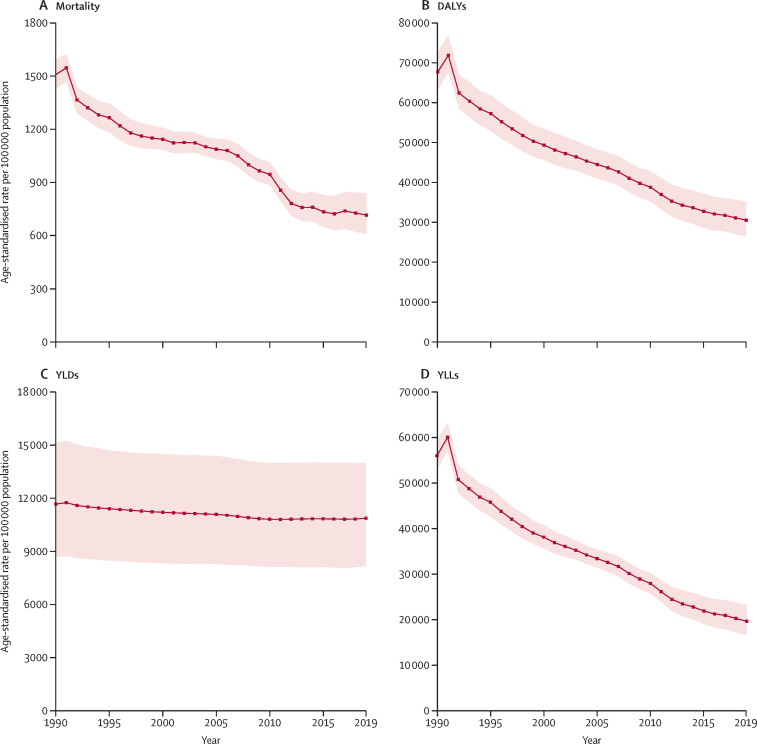
Age-standardised rates of mortality, DALYs, YLDs, and YLLs per 100 000 population in Bangladesh from 1990 to 2019 (A) Mortality. (B) DALYs. (C) YLDs. (D) YLLs. DALYs=disability-adjusted life-years. YLDs=years lived with disability. YLLs=years of life lost.

The age-standardised all-cause mortality rate in Bangladesh was 714·4 deaths (95% UI 604·9–838·2) per 100 000 population in 2019—a 52·7% (44·2–60·4) reduction in mortality rate relative to 1990 (1509·3 deaths [1428·6–1592·1]; figure 1). Deaths due to non-communicable diseases increased between 1990 and 2019, with 14 of the top 20 leading causes of death in 2019 due to such diseases (table 1). Stroke was the leading cause of death throughout 1990–2019; however, the mortality rate due to stroke declined by 22·8% (3·2–41·1) over this period. Although the mortality rate due to diabetes increased between 1990 and 2010, this rate subsequently decreased during 2010–19. Overall, deaths due to communicable diseases decreased substantially over time. Malaria was ranked as the tenth leading cause of death in 1990; however, by 2019, the associated mortality rate had decreased by 99·8% (97·4–100·0) and malaria was ranked the 116th leading cause of death. The mortality rate due to other vaccine-preventable diseases—including tetanus, diphtheria, and measles—also decreased between 1990 and 2019. Additionally, deaths due to drowning decreased by 77·6% (68·5–83·6) during this period.

**Table 1:** Age-standardised mortality rates with percentage changes from 1990 to 2010, 2010 to 2019, and 1990 to 2019 for the leading causes of diseases, disabilities, and injuries in Bangladesh

	**Rank**		**Deaths per 100 000 population**			**Percentage change in age-standardised deaths**		
	1990	2019	1990	2010	2019	1990–2010	2010–19	1990–2019
Stroke	1	1	181·4 (153·9 to 206·7)	199·8 (146·7 to 223·6)	140·0 (107·0 to 169·5)	10·1% (−17·6 to 30·6)	−29·9% (−41·4 to −16·1)	−22·8% (−41·1 to −3·2)
Ischaemic heart disease	4	2	114·3 (97·1 to 129·9)	125·7 (105·2 to 138·7)	111·2 (86·8 to 135·2)	10·0% (−5·2 to 30·5)	−11·5% (−27·3 to 5·9)	−2·7% (−23·0 to 23·1)
COPD	5	3	109·2 (84·5 to 160·0)	68·8 (54·1 to 120·5)	49·5 (36·8 to 86·5)	−37·0% (−46·3 to −21·6)	−28·0% (−40·4 to −13·8)	−54·6% (−65·1 to −41·5)
Lower respiratory infections	6	4	90·5 (79·8 to 101·5)	46·0 (40·6 to 52·2)	31·9 (25·8 to 37·7)	−49·1% (−56·3 to −39·8)	−30·7% (−40·4 to −20·6)	−64·7% (−71·9 to −56·5)
Neonatal disorders	7	5	76·8 (66·9 to 87·4)	51·9 (42·3 to 62·7)	31·2 (23·4 to 40·1)	−32·4% (−47·4 to −15·0)	−39·9% (−50·2 to −28·3)	−59·4% (−70·3 to −46·8)
Diarrhoeal diseases	2	6	166·1 (110·4 to 234)	46·1 (23 to 92·7)	31·2 (14·0 to 67·9)	−72·2% (−84·4 to −46·8)	−32·4% (−47·1 to −14·7)	−81·2% (−91·1 to −60·5)
Diabetes	12	7	29·1 (24·8 to 33·4)	35·8 (31·1 to 40·2)	30·8 (24·6 to 37·2)	23·0% (5·2 to 45·8)	−14·0% (−28·4 to 2·6)	5·8% (−17·2 to 34·7)
Tuberculosis	3	8	135·2 (113·5 to 156·8)	41·9 (34·8 to 51·5)	22·6 (17·4 to 30·8)	−69·0% (−74·2 to −60·9)	−46·1% (−55·8 to −34·1)	−83·3% (−87·1 to −76·6)
Cirrhosis	9	9	46·2 (37·1 to 55·4)	27·4 (23·5 to 31·9)	20·2 (15·8 to 25·6)	−40·8% (−50·3 to −26·3)	−26·2% (−39·4 to −9·6)	−56·3% (−67·0 to −41·5)
Alzheimer's disease	18	10	17·0 (4·0 to 49·3)	18·4 (4·3 to 50·3)	18·2 (4·5 to 49·3)	8·4% (−4·5 to 25·7)	−1·2% (−14·5 to 13·4)	7·2% (−9·5 to 31·1)
Hypertensive heart disease	14	11	21·5 (12·2 to 30·7)	18·4 (11·9 to 26·8)	15·9 (9·6 to 24·5)	−14·4% (−36 to 23·7)	−13·8% (−31·8 to 7·7)	−26·2% (−52·0 to 20·9)
Other malignant neoplasms	17	12	17·5 (7·2 to 24·8)	15·6 (7·2 to 21·2)	15·1 (6·8 to 21·9)	−10·6% (−28·8 to 13·1)	−3·5% (−22·5 to 19·3)	−13·8% (−39·5 to 22·3)
Chronic kidney disease	22	13	13·7 (10·1 to 16·6)	13·4 (11·8 to 15·1)	10·9 (8·7 to 13·3)	−1·8% (−19·4 to 29·8)	−18·7% (−32·6 to −3·7)	−20·2% (−38·1 to 8·1)
Asthma	11	14	32·6 (22·3 to 49·9)	13·9 (9·8 to 22·3)	8·8 (6·2 to 14·5)	−57·4% (−67·4 to −40·9)	−36·3% (−51·4 to −17)	−72·9% (−82·1 to −57·1)
Congenital defects	19	15	16·5 (10·1 to 23·4)	11·3 (7·7 to 15·6)	8·3 (4·7 to 13·8)	−31·3% (−57·5 to 30·5)	−26·8% (−49·9 to 1·8)	−49·7% (−75·2 to 12·6)
Lung cancer	28	16	9·7 (6·9 to 12·6)	8·1 (6·0 to 10·9)	7·8 (5·2 to 12·1)	−16·0% (−35·8 to 10·8)	−3·6% (−28·8 to 26)	−19·1% (−46·0 to 15·0)
Breast cancer	32	17	6·9 (5·3 to 8·7)	6·9 (5·9 to 8·1)	7·2 (5·7 to 9·0)	0·0% (−22·6 to 37·8)	4·2% (−17·6 to 30·1)	4·2% (−23·6 to 44·7)
Stomach cancer	27	18	11·6 (9·7 to 13·3)	8·2 (7·1 to 9·9)	6·6 (5·1 to 8·6)	−29·3% (−40·3 to −12·3)	−19·4% (−34·6 to −2·1)	−43·0% (−56·0 to −24·1)
Road injuries	29	19	9·2 (7·2 to 12·5)	7·2 (5·8 to 8·2)	5·6 (4·1 to 7·0)	−22·1% (−45·3 to 0·3)	−21·3% (−34 to −5·1)	−38·7% (−61·6 to −14·4)
Drowning	13	20	22·7 (17·5 to 29)	11·6 (9·3 to 14·5)	5·1 (3·9 to 7·5)	−49·0% (−59·1 to −35)	−56·1% (−64·7 to −42·4)	−77·6% (−83·6 to −68·5)
Typhoid and paratyphoid	23	21	12·4 (6·1 to 22·1)	7·2 (3·7 to 11·8)	5·0 (2·5 to 8·3)	−41·9% (−52·4 to −23·7)	−30·9% (−43·2 to −19·7)	−59·8% (−70·4 to −44·3)
Maternal disorders	15	31	18·7 (13·1 to 23)	4·4 (3·6 to 5·2)	3·8 (2·8 to 4·9)	−76·5% (−81·9 to −66·7)	−14·8% (−35 to 10·6)	−79·9% (−86·0 to −71·2)
Acute hepatitis	21	33	14·3 (11·2 to 17·3)	3·8 (3·2 to 4·5)	3·3 (2·5 to 4·4)	−73·6% (−79·5 to −65·5)	−11·6% (−32·6 to 15·4)	−76·7% (−83·7 to −66·0)
Protein-energy malnutrition	8	45	56·4 (37·8 to 75·7)	6·5 (5·3 to 7·9)	2·0 (1·4 to 2·7)	−88·5% (−92·0 to −82·3)	−68·5% (−76·7 to −57·6)	−96·4% (−97·9 to −93·6)
Upper digestive diseases	16	58	18·3 (14·0 to 23·1)	2·2 (1·7 to 2·6)	1·3 (1·0 to 1·7)	−88·0% (−90·6 to −84·3)	−40·3% (−53·8 to −25·0)	−92·8% (−95·1 to −89·6)
Tetanus	20	85	14·8 (10·8 to 19·9)	0·3 (0·2 to 0·6)	0·4 (0·1 to 0·7)	−97·8% (−98·9 to −96·2)	26·1% (−37·7 to 133·8)	−97·2% (−99·1 to −94·7)
Malaria	10	116	39·9 (5·3 to 178·3)	3·6 (1·9 to 7·2)	0·1 (0·0 to 0·4)	−90·9% (−97·4 to −32·2)	−98·1% (−100·0 to −86·3)	−99·8% (−100·0 to −97·4)

Data in parentheses are 95% uncertainty intervals. COPD=chronic obstructive pulmonary disease.

In 1990, the rate of mortality in children younger than 5 years (hereafter referred to as under-5 mortality rate) in Bangladesh was 132·9 deaths (95% UI 124·8–142·0) per 1000 livebirths. This rate decreased over subsequent years to 29·1 deaths (24·9–34·2) per 1000 livebirths in 2019 (appendix 2 p 34). Neonatal disorders and lower respiratory infections were the top two causes of death in this age group in both 1990 and 2019. Other major causes of death in 2019 included congenital defects, typhoid and paratyphoid diseases, diarrhoeal diseases, and drowning (appendix 2 p 38).

Age-standardised DALYs (representing the combined fatal and non-fatal burden) in Bangladesh decreased by 54·9% (48·8–60·4) from 67 634·8 DALYs per 100 000 population (95% UI 63 291·5–72 763·3) in 1990 to 30 475·3 DALYs per 100 000 population (26 366·7–35 154·8) in 2019 (figure 1). CMNN diseases contributed the most towards DALYs in Bangladesh. Neonatal disorders remained the top-ranked cause of DALYs between 1990 and 2019. DALYs from vaccine-preventable diseases decreased substantially between 1990 and 2019, as did DALYs due to other CMNN diseases such as diarrhoeal diseases, tuberculosis, typhoid, and paratyphoid. DALYs attributed to nutritional disorders (such as protein-energy malnutrition) and injuries (including drowning and road injuries) decreased between 1990 and 2019. In 2019, 14 of the top 20 leading causes of DALYs were non-communicable diseases. Stroke (2701·1 DALYs per 100 000 population [2095·8–3292·8]), ischaemic heart disease (2328·3 [1839·2–2875·3]), chronic obstructive pulmonary disease (COPD; 1144·2 [921·6–1732·7]), diabetes (934·3 [767·2–1138·8]), and other musculoskeletal disorders (845·1 [586·1–1154·0]) were the major contributors of DALYs attributed to non-communicable diseases in Bangladesh in 2019 (table 2).

**Table 2:** Age-standardised rates of DALYs with percentage changes from 1990 to 2010, 2010 to 2019, and 1990 to 2019 for the leading causes of diseases, disabilities, and injuries in Bangladesh

	**Rank**		**DALYs per 100 000 population**			**Percentage change in age-standardised DALYs**		
	1990	2019	1990	2010	2019	1990–2010	2010–19	1990–2019
Neonatal disorders	1	1	7058·3 (6169·6 to 7987·7)	4915·9 (4067·0 to 5855·8)	3217·6 (2520·6 to 3984·8)	−30·4% (−44·9 to −13·4)	−34·5% (−44·4 to −23·6)	−54·4% (−65·5 to −41·7)
Stroke	5	2	3805·9 (3253·9 to 4303·7)	3683·5 (2825·6 to 4097·5)	2701·1 (2095·8 to 3292·8)	−3·2% (−25·9 to 14·9)	−26·7% (−39·0 to −12·4)	−29·0% (−46·0 to −10·5)
Ischaemic heart disease	7	3	2504·6 (2114·5 to 2848·5)	2649·3 (2257·4 to 2947·2)	2328·3 (1839·2 to 2875·3)	5·8% (−9·5 to 27·4)	−12·1% (−29·2 to 7·2)	−7·0% (−27·8 to 21·1)
Lower respiratory tract infections	3	4	5492·6 (4732·3 to 6307·9)	2272·1 (1945·7 to 2648·6)	1291·4 (1002·5 to 1609·4)	−58·6% (−66·5 to −48·8)	−43·2% (−54·5 to −31·1)	−76·5% (−83·1 to −69·0)
COPD	8	5	2404·4 (1954·8 to 3144·6)	1439·6 (1130·5 to 2221·6)	1144·2 (921·6 to 1732·7)	−40·1% (−51·1 to −26·1)	−20·5% (−33·8 to −2·9)	−52·4% (−61·4 to −41·2)
Diabetes	18	6	869·3 (743·9 to 1009·8)	1007·7 (870·1 to 1170·7)	934·3 (767·2 to 1138·8)	15·9% (2·8 to 31·5)	−7·3% (−17·8 to 4·2)	7·5% (−9·2 to 26·8)
Diarrhoeal diseases	2	7	5634·5 (4375·6 to 7110·4)	1212·9 (778·8 to 2071·4)	846·9 (518·7 to 1483·9)	−78·5% (−86·1 to −64·9)	−30·2% (−41·2 to −15·6)	−85·0% (−90·7 to −74·0)
Other musculoskeletal disorders	24	8	684·0 (475·8 to 939·6)	796·3 (552·3 to 1087·6)	845·1 (586·0 to 1154·0)	16·4% (10·3 to 22·4)	6·1% (−0·7 to 12·8)	23·6% (14·4 to 31·7)
Depressive disorders	19	9	850·6 (578·6 to 1183·1)	812·8 (562·6 to 1147·7)	822·2 (563·0 to 1140·2)	−4·4% (−12·6 to 4·3)	1·2% (−8·2 to 11·6)	−3·3% (−9·4 to 3·4)
Congenital defects	12	10	1482·0 (919·9 to 2083·1)	1030·4 (717·8 to 1404·3)	763·8 (452·9 to 1226·9)	−30·5% (−56·1 to 29·0)	−25·9% (−48·0 to 1·6)	−48·5% (−73·7 to 10·7)
Low back pain	20	11	821·2 (574·5 to 1096)	792·1 (560·9 to 1074·1)	761·0 (536·3 to 1022·9)	−3·5% (−7·5 to 0·6)	−3·9% (−8·3 to 0·7)	−7·3% (−11·6 to −2·6)
Tuberculosis	4	12	4559·6 (3721·1 to 5314·9)	1294·5 (1089·1 to 1583·2)	687·4 (541·9 to 918·0)	−71·6% (−76·0 to −64·3)	−46·9% (−55·4 to −36·4)	−84·9% (−88·2 to −79·4)
Cirrhosis	11	13	1511·5 (1182·6 to 1831·1)	803·5 (689·4 to 940·3)	583·5 (450·6 to 749·6)	−46·8% (−55·8 to −31·4)	−27·4% (−40·5 to −11·5)	−61·4% (−71·3 to −46·7)
Headache disorders	29	14	563·0 (93·9 to 1243·5)	570·6 (93·2 to 1249·5)	574·3 (94·1 to 1279·4)	1·3% (−2·1 to 4·8)	0·7% (−2·7 to 4·1)	2·0% (−1·8 to 5·4)
Road injuries	23	15	739·4 (601·0 to 909·6)	622·2 (517·7 to 729·3)	559·2 (443·5 to 676·8)	−15·9% (−33·5 to −1·0)	−10·1% (−18·0 to −1·5)	−24·4% (−42·5 to −7·8)
Age-related hearing loss	30	16	537·8 (366·6 to 764·3)	520·4 (353·7 to 734·8)	507·0 (344·7 to 719·1)	−3·2% (−6·5 to 0·0)	−2·6% (−5·7 to 0·4)	−5·7% (−9·3 to −2·1)
Dietary iron deficiency	21	17	815·1 (538·2 to 1188)	576·0 (374·3 to 841·5)	488·7 (313·2 to 738·7)	−29·3% (−38·2 to −20·2)	−15·2% (−22·8 to −6·4)	−40·0% (−48·5 to −29·8)
Blindness and vision loss	28	18	564·7 (394·6 to 782·3)	480·9 (331·7 to 670·0)	447·7 (305·6 to 632·6)	−14·9% (−18·6 to −10·8)	−6·9% (−9·8 to −3·6)	−20·7% (−24·9 to −15·8)
Other malignant neoplasms	31	19	520·0 (251·9 to 705·5)	463·6 (240·4 to 614·3)	445·5 (224·5 to 633·0)	−10·9% (−29·7 to 14·2)	−3·9% (−21·8 to 17·4)	−14·3% (−40·9 to 23·0)
Typhoid and paratyphoid	16	21	938·4 (453·1 to 1659·3)	550·1 (282·8 to 910·2)	377·0 (188·9 to 630·1)	−41·4% (−52·7 to −21·5)	−31·5% (−44·5 to −19·4)	−59·8% (−71·0 to −43·6)
Drowning	10	23	1667·1 (1226·6 to 2153·5)	805·4 (633·4 to 1008·6)	311·4 (235·4 to 457·6)	−51·7% (−62·4 to −37·1)	−61·3% (−69·5 to −47·8)	−81·3% (−86·8 to −72·6)
Asthma	17	27	870·6 (632·1 to 1225·4)	359·6 (274·2 to 530·7)	243·4 (181·9 to 362·6)	−58·7% (−67·0 to −42·9)	−32·3% (−46·2 to −16·1)	−72·0% (−80·2 to −57·4)
Maternal disorders	14	28	1143·6 (823·7 to 1390·5)	282·5 (236·6 to 334·6)	237·6 (183·0 to 301·0)	−75·3% (−80·7 to −65·6)	−15·9% (−34·5 to 7·0)	−79·2% (−85·1 to −70·8)
Protein-energy malnutrition	6	43	2883·4 (2102·2 to 3780)	345·2 (268·3 to 445·9)	140·0 (98·3 to 193·6)	−88·0% (−91·9 to −82·1)	−59·5% (−70·0 to −43·0)	−95·1% (−97·1 to −91·9)
Tetanus	13	107	1249·7 (903·1 to 1683·8)	19·8 (12·2 to 31·4)	28·1 (10·4 to 55·1)	−98·4% (−99·1 to −97·3)	42·4% (−34·4 to 192·4)	−97·7% (−99·1 to −95·3)
Measles	15	119	996·2 (317·3 to 2257·6)	112·4 (37·2 to 262·3)	21·6 (6·3 to 51·5)	−88·7% (−93·0 to −82·6)	−80·7% (−90·0 to −65·4)	−97·8% (−98·9 to −96·2)
Malaria	9	146	1977·4 (295·7 to 7983·2)	191·4 (101·7 to 371·6)	4·6 (0·9 to 22·6)	−90·3% (−97·1 to −35·8)	−97·6% (−99·5 to −86·1)	−99·8% (−100·0 to −97·3)

Data in parentheses are 95% uncertainty intervals. COPD=chronic obstructive pulmonary disease. DALYS=disability-adjusted life-years.

The rate of age-standardised YLDs per 100 000 population (representing the non-fatal burden) was 11 667·2 (95% UI 8684·2–15 122·6) in 1990 and 10 865·3 (8169·9–14 008·8) in 2019—a decrease of 6·9% (4·5–9·1) between these years (figure 1, table 3). In 2019, non-communicable diseases accounted for 13 of the 20 leading causes of YLDs (appendix 2 p 36). Musculoskeletal disorders, depressive disorders, low back pain, headache disorders, age-related hearing loss, dietary iron deficiency, blindness and vision loss, and gynaecological disorders all remained in the top ten leading causes of YLDs between 1990 and 2019. During this time, the rates of YLDs attributed to neonatal disorders increased by 90·1% (47·3–146·9), tuberculosis decreased by 73·3% (69·8–76·5), and road injuries decreased by 5·9% (2·8–8·9).

**Table 3:** Age-standardised rate of mortality, YLLs, and YLDs, and life expectancy at birth and HALE at <1 year of age for male and female sexes combined in Bangladesh and other countries in the south Asia region, 1990 and 2019

	**Deaths per 100 000 population (age-standardised)**				**YLLs per 100 000 population (age-standardised)**				**YLDs per 100 000 population (age-standardised)**				**Life expectancy at birth (years)**				**HALE at <1 year (years)**			
	1990		2019		1990		2019		1990		2019		1990		2019		1990		2019	
	Rate	Rank	Rate	Rank	Rate	Rank	Rate	Rank	Rate	Rank	Rate	Rank	Life expectancy	Rank	Life expectancy	Rank	HALE	Rank	HALE	Rank
Bhutan	1390·5 (1211·7–1599·0)	2	818·0 (687·7–942·3)	2	53 021·1 (44 665·2–62 243·2)	2	21 611·9 (17 087·6–26 875·5)	2	12 007·4 (8914·4–15 485·5)	3	11 389·3 (8481·9–14 672·0)	2	60·3 (57·7–62·9)	2	73·2 (70·9–75·6)	2	52·3 (49·1–55·3)	2	63·2 (59·9–66·3)	2
India	1593·8 (1531·1–1659·1)	5	906·6 (818·3–1002·0)	3	53 250·6 (50 601·1–55 996·1)	3	25 749·0 (23 179·5–28 541·4)	4	13 081·2 (9756·6–16 904·8)	5	12 094·3 (9038·8–15 665·3)	5	59·6 (58·7–60·5)	3	70·8 (69·3–72·2)	4	51·1 (48·5–53·3)	3	60·5 (57·4–63·3)	4
Bangladesh	1509·3 (1428·6–1592·1)	3	714·4 (604·9–838·2)	1	55 967·6 (52 916·4–59 448·6)	4	19 610·0 (16 543·8–23 230·0)	1	11 667·2 (8684·2–15 122·6)	2	10 865·3 (8169·9–14 008·8)	1	58·2 (57·1–59·2)	5	74·6 (72·4–76·7)	1	50·7 (48·3–52·8)	4	64·5 (61·3–67·4)	1
Nepal	1539·1 (1388·2–1718·6)	4	959·8 (825·2–1052·2)	4	57 731·3 (52 798·0–63 646·7)	5	24 555·1 (21 042·5–27 706·6)	3	12 292·7 (9124·3–15 840·0)	4	11 397·9 (8510·3–14 688·3)	3	58·3 (56·3–60·3)	4	71·1 (69·4–73·2)	3	50·4 (47·7–53·1)	5	61·5 (58·6–64·4)	3
Pakistan	1373·1 (1306·5–1442·1)	1	1149·9 (1023·7–1309·1)	5	50 045·3 (47 356·9–52 913·7)	1	36 577·5 (32 326·9–41 194·7)	5	11 475·8 (8517·0–14 783·6)	1	11 444·6 (8560·3–14 792·2)	4	61·1 (60·0–62·1)	1	65·9 (63·8–67·8)	5	53·2 (50·8–55·3)	1	57·2 (54·3–60·1)	5

Data in parentheses are 95% uncertainty intervals. 1 indicates the best performance in population health and 5 the worst. HALE=healthy life expectancy. YLDs=years lived with disability. YLLs=years of life lost.

The rate of age-standardised YLLs per 100 000 population (representing premature deaths and the fatal burden) decreased by 65·0% (95% UI 58·2–70·9) between 1990 and 2019. Age-standardised YLLs for all causes were 55 967·6 (52 916·4–59 448·6) in 1990 and 19 610·0 (16 543·8–23 230·0) in 2019 (figure 1, table 3). Overall, YLLs decreased between 1990 and 2019 for all causes except Alzheimer's disease (appendix 2 p 38). YLLs from vaccine-preventable diseases declined substantially between 1990 and 2019, with the largest decrease observed for malaria (99·8% [97·4–100·0]; table 1). During this time, YLLs due to nutritional disorders (such as protein-energy malnutrition) and road injuries also decreased.

**Figure 2:**
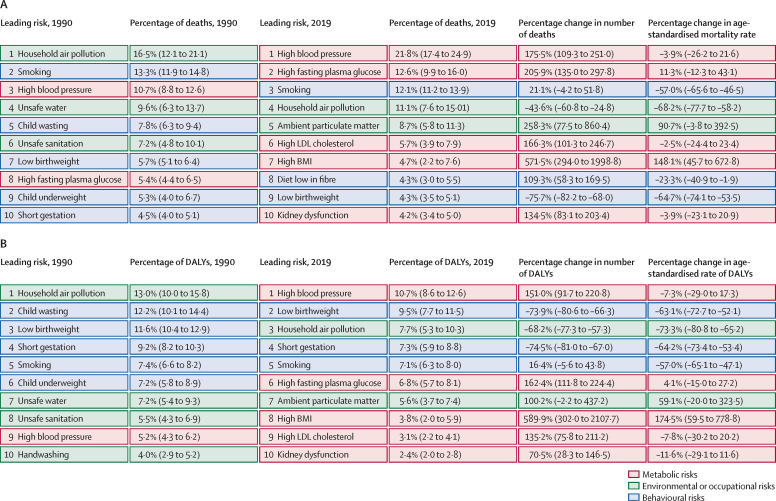
Major risk factors associated with age-standardised all-cause mortality and DALYs in Bangladesh between 1990 and 2019 Data in parentheses are 95% uncertainty intervals. Ranks are based on age-standardised percentage of deaths and DALYs associated with the risk factors; detailed estimates are available via the GBD Compare and GBD Results tools. DALYs=disability-adjusted life-years. GBD=Global Burden of Diseases, Injuries, and Risk Factors Study.

In 2019, of the top ten risk factors for all-cause mortality, five were metabolic, three were behavioural, and two were environmental and occupational (figure 2). High systolic blood pressure was the leading metabolic risk factor for all-cause mortality, accounting for 21·8% (95% UI 17·4–24·9) of age-standardised mortality. Smoking, the leading behavioural risk factor, accounted for 12·1% (11·2–13·9) of deaths. Household air pollution from solid fuels was the leading environmental and occupational risk factor and accounted for 11·1% (7·6–15·1) of age-standardised all-cause mortality. In 1990, of the top ten risk factors for all-cause DALYs, only one (high systolic blood pressure, ranked ninth) was a metabolic risk. In 2019, high systolic blood pressure was also ranked as the number one risk factor, accounting for 10·7% (8·6–12·6) of DALYs (figure 2).

Estimates for Bangladesh were compared with those for the other countries within the GBD south Asia region—Bhutan, India, Nepal, and Pakistan. Of these countries, Bangladesh had the lowest rate of age-standardised mortality, YLLs, and YLDs in 2019 (the 95% UIs for some metrics overlap for some countries in these rankings; appendix 2 p 19). However, Bangladesh ranked among the worst of these countries in terms of DALYs caused by stroke, diabetes, depressive disorders, and congenital defects. Most of the compared countries have a similar profile in terms of causes of age-standardised DALYs between 1990 and 2019, with neonatal disorders being among the top causes throughout these years (appendix 2 p 13). However, Bangladesh had the highest age-standardised mortality rates of stroke in 2019 in south Asia (appendix 2 p 40).

Bangladesh ranks better than most other south Asian countries in terms of DALYs caused by tuberculosis, liver cirrhosis, and dietary iron deficiency disorder. In 1990, Bangladesh ranked worst (fifth) within the compared countries in terms of life expectancy at birth; however, in 2019, Bangladesh had the highest life expectancy at birth and ranked first for HALE in south Asia (appendix 2 pp 20, 21).

In 1990, Bangladesh had the second-highest under-5 mortality rate of the compared countries in south Asia, with Nepal ranking first. However, over the years, this rate has decreased substantially in both countries. In 2019, Bangladesh had the second-lowest under-5 mortality rate (after Nepal) in the region (appendix 2 p 16). In 2019, Bangladesh also had the lowest total fertility rate in south Asia (appendix 2 p 18). Health expenditure across the five countries differs considerably, with India and Pakistan spending the most and Bhutan spending the least (appendix 2 p 113).

## Discussion

This study is the first, to our knowledge, to provide a comprehensive and systematic analysis of disease burden and trends in Bangladesh. Our analysis shows that Bangladesh has made remarkable achievements in reducing mortality rates and health loss by more than half from 1990 to 2019. However, the prevalence of non-communicable diseases—including stroke, ischaemic heart disease, COPD, diabetes, and chronic kidney disease—has increased steadily since 1990. Although downward trends were observed in deaths due to CMNN diseases, including malaria and measles, four infectious diseases remained in the top ten causes of mortality in 2019. Musculoskeletal disorders, low back pain, diabetes, and neonatal disorders were the leading causes of non-fatal burden from 1990 to 2019. Our results highlight an increase in premature mortality from Alzheimer's disease between 1990 and 2019, whereas premature mortality due to stroke, ischaemic heart disease, COPD, and diabetes declined during this time period. Substantial downward trends in premature mortality were observed for diarrhoeal diseases, tetanus, measles, and malaria, demonstrating success in Bangladesh's communicable diseases programmes. Additionally, life expectancy at birth and HALE increased in Bangladesh from 1990 to 2019. These findings reflect improved health-promotion activities and the increase in health-care service provision nationwide. However, Bangladesh still faces a double burden of communicable and non-communicable diseases. If the current trend continues, management of the increased burden of non-communicable diseases will be a considerable challenge for the country's health system.

Over the past three decades, the total number of deaths due to non-communicable diseases in Bangladesh has decreased, which is consistent with previous reports.^28^ Despite this trend, non-communicable diseases remain a substantial cause of death and should be a health-care priority. The prevalence and mortality rates of diabetes and breast cancer increased slightly, which is concerning and warrants further investigation. The 2018 National STEPS Survey for Non-communicable Diseases Risk Factors in Bangladesh, conducted by WHO, reported that 21% of adults had hypertension and 8**·**4% had diabetes.^29^ Our findings differ from estimates of the prevalence of and deaths from non-communicable disease provided by the World Bank, the Ministry of Health and Family Welfare, and WHO owing to differences in data sources, the definition and assignment of causes of death and disability, and analysis and modelling methods. We believe that our rigorous methodology and extensive validation produce the most comprehensive estimates.

Health loss attributed to the leading causes, as measured by DALYs, decreased between 1990 and 2019, with steep declines for protein-energy malnutrition, tetanus, measles, malaria, diarrhoeal diseases, and drowning. This achievement might reflect successful public health policies and practices focused in those areas (eg, vaccination and sanitation programmes). Although drowning rates have decreased since 1990, Bangladesh still has the highest rate of child drowning in south Asia, with nearly 46 children aged 0–17 years drowning each day in 2016.^30^ Diabetes and musculoskeletal disorders increased substantially from 1990 to 2019 and rose in ranking of the causes of health loss. Other areas of concern include stroke (ranked second in 2019), ischaemic heart disease (ranked third), and diabetes (ranked sixth). Health loss due to tuberculosis, asthma, and COPD substantially reduced from 1990 to 2019, while smaller reductions were observed for road injuries and other malignant neoplasms. Despite having tertiary facilities, services for non-communicable disease prevention and rehabilitation are insufficient in Bangladesh. Nonetheless, some innovative measures are being tested. The Control of Blood Pressure and Risk Attenuation-Bangladesh, Pakistan, Sri Lanka (COBRA-BPS) trial, led by Jafar and colleagues,^31^, ^32^ found that a multicomponent intervention led by a community health worker was more successful and more cost-effective in lowering hypertension than standard care. Community-based trials using text messaging have been successful and cost-effective for diabetes management in urban and rural areas in Bangladesh.^10^, ^33^, ^34^ However, there are several challenges to the implementation of innovative measures, such as a limited health budget and the technical capacity of the health workforce. Evidence suggests that health facilities in Bangladesh lack the readiness to manage non-communicable diseases.^35^ Such issues must be addressed if Bangladesh is to achieve the Sustainable Development Goals target of reducing premature mortality from non-communicable diseases by a third by 2030.

During the past three decades, the Government of Bangladesh has worked to improve sanitation; provide safe drinking water, oral rehydration solution, and healthy diets and nutrients for children; promote high coverage of vaccines and use of insecticide-treated bednets; and improve maternal health services and pregnancy care. This success can be attributed to Bangladesh's pluralistic health-care system, involving participation from numerous national and international non-governmental organisations.^36^ For example, the Bangladesh Rural Advancement Committee (BRAC), a non-governmental organisation, had a pivotal role in reducing child mortality due to diarrhoea through its oral rehydration therapy work.^37^ These efforts have reduced mortality from most CMNN diseases (except neonatal disorders; mostly due to the scarcity of hospital facilities in rural and remote areas). Child mortality due to diarrhoea, undernutrition, and tetanus has decreased markedly. Among the whole population, tuberculosis, malaria, and measles are no longer top-ranked causes of mortality, resulting in a sharp reduction of premature mortality. In 2019, stroke and ischaemic heart disease were the leading causes of premature deaths in Bangladesh. Lung cancer, breast cancer, and stomach cancer have shown concerning increases, highlighting the importance of prevention—including screening for cancer in primary-care facilities. Prevention strategies for neonatal disorders and non-communicable diseases should remain a focus of health-care policy and practice. Bangladesh adopted a multisectoral action plan involving nearly 30 ministries and agencies to prevent and control non-communicable diseases from 2018 to 2025.^38^ Finally, road injuries and drowning remain considerable public health problems in Bangladesh and deserve more attention from policy makers.

Compared with the other countries in the south Asia region, Bangladesh had the highest life expectancy at birth, highest HALE, lowest mortality rate, lowest rates of premature mortality, and lowest non-fatal burden in 2019. Maternal health remains a considerable public health issue in Bangladesh and other south Asian countries: almost half of all maternal deaths worldwide occur in these countries.^39^ Multifaceted approaches for improving maternal health-care access and nutrition in south Asia are needed.

Our study is subject to the limitations of the GBD methodology, which have been described previously.^24^, ^26^ Additionally, our findings should be interpreted with the following caveats. First, Sample Vital Registration System data were not available in Bangladesh for all years within the studied time period, and data on risk factors, clinics, and private hospitals are scarce. Second, we could not conduct analyses at the division or district levels to examine disparities between rural and urban areas and among ethnic and migrant populations in Bangladesh. In the future, generating GBD estimates at the subnational level in Bangladesh will be essential. Third, our study did not include comparisons with Sri Lanka, Afghanistan, or the Maldives, because these countries are not included in the GBD south Asia region. Finally, the COVID-19 pandemic could have affected the burden of disease in Bangladesh. Although its effects on these estimates are negligible as we used data up until 2019, the pandemic might have changed the landscape regarding disease burden and health-care capacity in the country since 2020, which could have implications for future planning and interventions and policies.

In Bangladesh, life expectancy at birth has increased by more than 16 years since 1990, therefore increasing the ageing population. Bangladesh is now experiencing an epidemiological transition from CMNN diseases towards non-communicable diseases and disabilities. Multisectoral policies and practices prioritising the prevention and management of and rehabilitation from non-communicable diseases are mandatory to control the increase in mortality and morbidity. A focus on cardiovascular health, maternal health, musculoskeletal disorders, neurological diseases, and cancer care is necessary. Our findings can be used to prioritise health-care needs, identify gaps in health-care systems, and guide future research and investments to improve health outcomes in Bangladesh.

## Data sharing

To download the data used in these analyses, please visit the Global Health Data Exchange at http://ghdx.healthdata.org/gbd-2019.

## Declaration of interests

S M S Islam reports support for this manuscript from an Emerging Leadership Fellowship from the National Health and Medical Research Council of Australia (APP1195406) and Vanguard grants from the National Heart Foundation of Australia. S M S Islam has unpaid roles, outside the submitted work, with the IT Committee of the Cardiac Society of Australia and New Zealand, as a volunteer on the Cardiac Devices Committee of the ESC Heart Failure Association, and as a volunteer topic group leader for the WHO-ITU Global Initiative on AI for Health. K M Livingstone reports support for this manuscript from a National Health and Medical Research Council of Australia grant (APP1173803).

## Supplementary Material

Bangla translation of the abstract
