# Supplementary material for: The burden of diseases and risk factors in Bangladesh, 1990–2019: a systematic analysis for the Global Burden of Disease Study 2019
Source: Lancet Glob Health. 2023 Nov 14;11(12):e1931–42. doi: 10.1016/S2214-109X(23)00432-1 (PMC10664824; doi:10.1016/S2214-109X(23)00432-1)
Supplement: Bangla translation of the abstract [file mmc1.pdf]

THE LANCET
Global Health

Supplementary appendix 1

This translation in Bangla was submitted by the authors and we reproduce it as supplied. It has not been peer reviewed. *The Lancet*'s editorial processes have only been applied to the original in English, which should serve as reference for this manuscript.

'এই [বাংলায়] অনুবাদটি লেখকরা জমা দিয়েছিলেন এবং এটি যেমনভাবে দেওয়া হয়েছে আমরা সেইভাবেই পুনরায় বর্ণনা করছি। এটি কোনো সমকক্ষ ব্যক্তি পর্যালোচনা করেননি। দ্য ল্যানসেট-এর সম্পাদকীয় প্রক্রিয়াগুলি শুধুমাত্র মূল ইংরেজিতে প্রয়োগ করা হয়েছে, যা এই পান্ডুলিপির রেফারেন্স হিসাবে কাজ করবে।'

Supplement to: GBD 2019 Bangladesh Burden of Disease Collaborators. The burden of diseases and risk factors in Bangladesh, 1990–2019: a systematic analysis for the Global Burden of Disease Study 2019. *Lancet Glob Health* 2023; **11:** e1931–42.

সংক্ষেপিত

পটভূমি: বাংলাদেশ বিগত ৫০ বছরে আর্থ-সামাজিক অবস্থা ও স্বাস্থ্য সম্পর্কিত সূচকের উন্নয়নে উল্লেখযোগ্য অগ্রগতি করেছে, কিন্তু জাতীয় পর্যায়ে রোগের বোঝা সম্পর্কে প্রয়োজনীয় তথ্যের অভাব রয়েছে। আমরা গ্লোবাল বার্ডেন অফ ডিজিজ গবেষণা পদ্ধতি ব্যবহার করে ১৯৯০ থেকে ২০১৯ সন পর্যন্ত বাংলাদেশের রোগের বিস্তার এবং ঝুঁকির কারণগুলি মূল্যায়ন করেছি।

গবেষনা পদ্ধতি: এই গবেষণার জন্য, আমরা অত্যাবশ্যক রেজিস্ট্রেশন সিস্টেম, জরিপ, এবং আদমশুমারি থেকে প্রাপ্ত তথ্য নিয়ে মাল্টিস্টেজ মডেলিং প্রক্রিয়া ব্যবহার করে আনুমানিক মৃত্যুহার, জীবনের অতিবাহিত বছরগুলো (YLLs), অক্ষমতার সাথে বেঁচে থাকা বছরগুলো (YLDs) এবং অক্ষমতার সাথে সমন্বয় করা বছরগুলো (DALYs) নির্নয় করেছি। উপরন্তু, আমরা দক্ষিণ এশিয়ার অন্যান্য দেশের সঙ্গে বাংলাদেশের স্বাস্থ্য ব্যবস্থার তুলনা করেছি- ভুটান, ভারত, নেপাল এবং পাকিস্তান।

ফলাফল: বাংলাদেশে জন্মকালীন গড় আয়ু ১৯৯০ সনের ৫৮.২ বছর (৯৫% কনফিডেন্স ইন্টারভাল ৫৭.১–৫৯.২) থেকে বেড়ে ২০১৯ সানে ৭৪.৬ বছর (৭২.৪-৭৬.৭) হয়েছে। একই সঙ্গে এই সময়ের মধ্যে দেশে বয়স অনুপাতে মৃত্যুহার প্রতি লাখে ১৫০৯.৩ জন (১৪২৮.৬-১৫৯২.১) থেকে কমে ৭১৪.৪ জন (৬০৪.৯-৮৩৮.২) হয়েছে। ২০১৯ সালে, মৃত্যুর শীর্ষ ২০টি কারণের মধ্যে ১৪টিই হচ্ছে বিভিন্ন অসংক্রামক রোগের কারনে। এর মধ্যে স্ট্রোক, ইস্কেমিক হার্ট ডিজিজ এবং ক্রনিক অবস্ট্রাকটিভ পালমোনারি ডিজিজ ছিল মৃত্যুর শীর্ষস্থানীয় কারণ। রোগসংক্রান্ত ঝুকির মাঝে উচ্চ রক্তচাপ, রক্তে উচ্চ শর্করা এবং ধূমপান ছিল শীর্ষ ঝুঁকির কারণ। ১৯৯০ সালের তুলনায় ২০১৯ সালে দেশে DALYs হার কমে ৫৪.৯% (৪৮.৮ – ৬০.৪) দাঁড়িয়েছে। ২০১৯ সালে, DALYs এবং YLLs এর প্রধান কারণগুলোর মধ্যে নিউনেটাল ডিজঅর্ডার, স্ট্রোক এবং ইস্কেমিক হার্ট ডিজিজ; পেশী ও হাড়ের রোগ, বিষণ্নতাজনিত রোগ, এবং পিঠের নিম্নভাগের ব্যথা YLDs এর প্রধান কারণ ছিল। দক্ষিণ এশিয়ার অন্যান্য দেশের তুলনায় বাংলাদেশে মৃত্যুহার, YLDs ও YLLs সবচেয়ে কম এবং গড় আয়ু সবচেয়ে বেশি।

ব্যাখ্যা: গত ৩০ বছরে বাংলাদেশে মৃত্যুহার অর্ধেকেরও বেশি কমেছে। তবে, বাংলাদেশকে এখন সংক্রামক এবং অসংক্রামক রোগের দ্বিগুণ বোঝা মোকাবেলা করতে হবে। অসংক্রামক রোগ প্রতিরোধ ও নিয়ন্ত্রণ, স্বাস্থ্যকর জীবনযাত্রাকে উৎসাহিত করা এবং অকাল মৃত্যু ও অক্ষমতা প্রতিরোধের জন্য সাশ্রয়ী বহুমাত্রিক প্রচেষ্টার প্রয়োজন পরবে।
