# Supplementary material for: The burden of diseases and risk factors in Bangladesh, 1990–2019: a systematic analysis for the Global Burden of Disease Study 2019
Source: Lancet Glob Health. 2023 Nov 14;11(12):e1931–42. doi: 10.1016/S2214-109X(23)00432-1 (PMC10664824; doi:10.1016/S2214-109X(23)00432-1)
Supplement: Supplementary appendix 2 [file mmc2.pdf]

THE LANCET
Global Health

Supplementary appendix 2

This appendix formed part of the original submission and has been peer reviewed. We post it as supplied by the authors.

Supplement to: GBD 2019 Bangladesh Burden of Disease Collaborators. The burden of diseases and risk factors in Bangladesh, 1990–2019: a systematic analysis for the Global Burden of Disease Study 2019. *Lancet Glob Health* 2023; **11:** e1931–42.

Supplementary appendix

Appendix 1: Supplementary methods and results to "The burden of diseases and risk factors in Bangladesh, 1990–2019: a systematic analysis for the Global Burden of Disease Study 2019"

This appendix provides supplemental figures and more detailed results for "The burden of diseases and risk factors in Bangladesh, 1990–2019: a systematic analysis for the Global Burden of Disease Study 2019".

Portions of this appendix have been reproduced or adapted from GBD 2019 Diseases and Injuries Collaborators.[1] References are provided for reproduced sections.

Table of Contents

List of Figures and Tables 5

Figures 5

Figure S1. Total fertility rate and under-5 mortality during 1990 and 2019: comparison between Bangladesh and south Asian countries 5

Figure S2A. Ranking of the leading causes of age-standardised rates of disability-adjusted life-years (DALYs) in Bangladesh compared to other south Asian countries, 1990. 5

Figure S2B. Ranking of the leading causes of age-standardised rates of disability-adjusted life-years (DALYs) in Bangladesh compared to other south Asian countries, 2010. 5

Figure S2C. Ranking of the leading causes of age-standardised rates of disability-adjusted life-years (DALYs) in Bangladesh compared to other south Asian countries, 2019. 5

Figure S3. Changes in ranking of causes of under-5 mortality in Bangladesh between 1990 and 2019 5

Figure S4. Population density of Bangladesh according to districts (census 2011) 5

Figure S5. Total Fertility Rate in Bangladesh, Bhutan, India, Nepal, and Pakistan 1990-2019 5

Figure S6. Deaths per 1000 live births in Bangladesh, Bhutan, India, Nepal, and Pakistan 1990-2019 5

Figure S7. Life expectancy at birth in Bangladesh, Bhutan, India, Nepal, and Pakistan 1990-2019 5

Figure S8. Healthy life expectancy in Bangladesh, Bhutan, India, Nepal, and Pakistan 1990-2019 5

Figure S9. Changes in age-standardised prevalence of diseases and injuries (level 1) in Bangladesh from 1990-2019 5

Figure S10. Changes in age-standardised prevalence of diseases and injuries (level 2) in Bangladesh from 1990-2019 5

Tables 5

Section 1. Statement of GATHER compliance 7

Guidelines for Accurate and Transparent Health Estimates Reporting (GATHER) checklist 7

Section 2. Methods Appendix 9

Systematic literature review and identification and selection of studies and data sources 9

DisMod-MR model development and validation 9

TMREL 11

Section 3. List of Figures.. 12

Figure S1. Total fertility rate and under-5 mortality during 1990 and 2019: comparison between Bangladesh and south Asian countries.. 12

Figure S2A. Ranking of the leading causes of age-standardised rates of disability-adjusted life-years (DALYs) in Bangladesh compared to other south Asian countries, 1990. 13

Figure S2B. Ranking of the leading causes of age-standardised rates of disability-adjusted life-years (DALYs) in Bangladesh compared to other south Asian countries, 2010. 14

Figure S2C. Ranking of the leading causes of age-standardised rates of disability-adjusted life-years (DALYs) in Bangladesh compared to other south Asian countries, 2019. 15

Figure S3. Changes in ranking of causes of under-5 mortality in Bangladesh between 1990 and 2019 16

Figure S4. Population density of Bangladesh according to districts (census 2011) 17

Figure S5. Total Fertility Rate in Bangladesh, Bhutan, India, Nepal, and Pakistan 1990-2019 18

Figure S6. Deaths per 1000 live births in Bangladesh, Bhutan, India, Nepal, and Pakistan 1990-2019 19

Figure S7. Life expectancy at birth in Bangladesh, Bhutan, India, Nepal, and Pakistan 1990-2019 ... 20

Figure S8. Healthy life expectancy in Bangladesh, Bhutan, India, Nepal, and Pakistan 1990-2019 ... 21

Figure S9. Changes in age-standardised prevalence of diseases and injuries (level 1) in Bangladesh from 1990-2019 22

Figure S10. Changes in age-standardised prevalence of diseases and injuries (level 2) in Bangladesh from 1990-2019 23

Section 4. Tables 24

Table S1. Total fertility rate, life expectancy at birth (years), healthy life expectancy at birth (years), and under-5 mortality (deaths per 1000 live births) during 1990 and 2019 in Bangladesh 24

Table S2. Prevalence of diseases and injuries in Bangladesh in 1990 and 2019 (Level 1 Causes).... 28

Table S3. Prevalence of diseases and injuries in Bangladesh in 1990 and 2019 (Level 2 Causes).... 29

Table S4. Annual rate of changes in prevalence of diseases and injuries in Bangladesh from 1990 and 2019 33

Table S5. Under 5 mortality (deaths per 1,000 live births) during 1990 and 2019: comparison between Bangladesh and South Asian countries 34

Table S6. Age-standardised rate YLDs with percentage changes between 1990 and 2010, 2010 and 2019, and 1990 and 2019 for the leading causes of diseases, disabilities, and injuries in Bangladesh 36

Table S7. Age-standardised rate YLLs with percentage changes between 1990 and 2010, 2010 and 2019, and 1990 and 2019 for the leading causes of diseases, disabilities, and injuries in Bangladesh 38

Table S8. Age-standardised rates of deaths for NCDs, cardiovascular diseases and stroke in south Asia in 2019 40

Table S9. Life expectancy at birth (years) during 1990 and 2019: comparison between Bangladesh and South Asian countries 41

Table S10. Healthy life expectancy (years) at birth during 1990 and 2019: comparison between Bangladesh and South Asian countries 43

Table S11. Total fertility rate during 1990 and 2019: comparison between Bangladesh and South Asian countries 45

Section 5. Data sources 47

Table S12. Data sources used for estimation of burden of diseases in Bangladesh. 47

Table S13. Bangladesh current healthcare expenditure in 2020 112

Table S14. Health expenditure and donor assistance for health in Bangladesh and comparator countries in 2020 113

Section 6. References 114

Section 7. Author’s Contributions 115

List of Figures and Tables

Figures

Figure S1. Total fertility rate and under-5 mortality during 1990 and 2019: comparison between Bangladesh and south Asian countries

Figure S2A. Ranking of the leading causes of age-standardised rates of disability-adjusted life-years (DALYs) in Bangladesh compared to other south Asian countries, 1990.

Figure S2B. Ranking of the leading causes of age-standardised rates of disability-adjusted life-years (DALYs) in Bangladesh compared to other south Asian countries, 2010.

Figure S2C. Ranking of the leading causes of age-standardised rates of disability-adjusted life-years (DALYs) in Bangladesh compared to other south Asian countries, 2019.

Figure S3. Changes in ranking of causes of under-5 mortality in Bangladesh between 1990 and 2019

Figure S4. Population density of Bangladesh according to districts (census 2011)

Figure S5. Total Fertility Rate in Bangladesh, Bhutan, India, Nepal, and Pakistan 1990-2019

Figure S6. Deaths per 1000 live births in Bangladesh, Bhutan, India, Nepal, and Pakistan 1990-2019

Figure S7. Life expectancy at birth in Bangladesh, Bhutan, India, Nepal, and Pakistan 1990-2019

Figure S8. Healthy life expectancy in Bangladesh, Bhutan, India, Nepal, and Pakistan 1990-2019

Figure S9. Changes in age-standardised prevalence of diseases and injuries (level 1) in Bangladesh from 1990-2019

Figure S10. Changes in age-standardised prevalence of diseases and injuries (level 2) in Bangladesh from 1990-2019

Tables

Table S1. Total fertility rate, life expectancy at birth (years), healthy life expectancy at birth (years), and under-5 mortality (deaths per 1000 live births) during 1990 and 2019 in Bangladesh

Table S2. Prevalence of diseases and injuries in Bangladesh in 1990 and 2019 (Level 1 Causes)

Table S3. Prevalence of diseases and injuries in Bangladesh in 1990 and 2019 (Level 2 Causes)

Table S4. Annual rate of changes in prevalence of diseases and injuries in Bangladesh from 1990 and 2019

Table S5. Under 5 mortality (deaths per 1,000 live births) during 1990 and 2019: comparison between Bangladesh and South Asian countries

Table S6. Age-standardised rate YLDs with percentage changes between 1990 and 2010, 2010 and 2019, and 1990 and 2019 for the leading causes of diseases, disabilities, and injuries in Bangladesh

Table S7. Age-standardised rate YLLs with percentage changes between 1990 and 2010, 2010 and 2019, and 1990 and 2019 for the leading causes of diseases, disabilities, and injuries in Bangladesh

Table S8. Age-standardised rates of deaths for NCDs, cardiovascular diseases and stroke in south Asia in 2019

Table S9. Life expectancy at birth (years) during 1990 and 2019: comparison between Bangladesh and South Asian countries

Table S10. Healthy life expectancy (years) at birth during 1990 and 2019: comparison between Bangladesh and South Asian countries

Table S11. Total fertility rate during 1990 and 2019: comparison between Bangladesh and South Asian countries

Table S12. Data sources used for estimation of burden of diseases in Bangladesh.

Table S13. Bangladesh current healthcare expenditure in 2020

Table S14. Health expenditure and donor assistance for health in Bangladesh and comparator countries in 2020

Section 1. Statement of GATHER compliance

This study complies with the Guidelines for Accurate and Transparent Health Estimates Reporting (GATHER) recommendations.[2]

Guidelines for Accurate and Transparent Health Estimates Reporting (GATHER) checklist

Item #	Checklist item	Reference
**Objectives and funding**		
1	Define the indicator(s), populations (including age, sex, and geographic entities), and time period(s) for which estimates were made.	Main text (Methods) and Appendix (Sections 2)
2	List the funding sources for the work.	Summary (Funding)
**Data Inputs**		
For all data inputs from multiple sources that are synthesized as part of the study:		
3	Describe how the data were identified and how the data were accessed.	Main text (Methods) and Appendix (Sections 2)
4	Specify the inclusion and exclusion criteria. Identify all ad-hoc exclusions.	Main text (Methods) and Appendix (Sections 2)
5	Provide information on all included data sources and their main characteristics. For each data source used, report reference information or contact name/institution, population represented, data collection method, year(s) of data collection, sex and age range, diagnostic criteria or measurement method, and sample size, as relevant.	Appendix (Sections 5) with additional information about these sources available at https://ghdx.healthdata.org
6	Identify and describe any categories of input data that have potentially important biases (e.g., based on characteristics listed in item 5).	Appendix (Sections 5)
For data inputs that contribute to the analysis but were not synthesized as part of the study:		
7	Describe and give sources for any other data inputs.	Global Health Data Exchange (https://ghdx.healthdata.org/)
For all data inputs:		
8	Provide all data inputs in a file format from which data can be efficiently extracted (e.g., a spreadsheet rather than a PDF), including all relevant meta-data listed in item 5. For any data inputs that cannot be shared because of ethical or legal reasons, such as third-party ownership, provide a contact name or the name of the institution that retains the right to the data.	Global Health Data Exchange (https://ghdx.healthdata.org/)
**Data analysis**		
9	Provide a conceptual overview of the data analysis method. A diagram may be helpful.	Main text (Methods) and Appendix (Sections 2)
10	Provide a detailed description of all steps of the analysis, including mathematical formulae. This description should cover, as relevant, data cleaning, data pre-processing, data adjustments and weighting of data sources, and mathematical or statistical model(s).	Main text (Methods) and Appendix (Sections 2)

**11**	Describe how candidate models were evaluated and how the final model(s) were selected.	Main text (Methods) and Appendix (Sections 2)
**12**	Provide the results of an evaluation of model performance, if done, as well as the results of any relevant sensitivity analysis.	Main text (Methods) and Appendix (Sections 2)
**13**	Describe methods for calculating uncertainty of the estimates. State which sources of uncertainty were, and were not, accounted for in the uncertainty analysis.	Main text (Methods) and Appendix (Sections 2)
**14**	State how analytic or statistical source code used to generate estimates can be accessed.	Remote code repository
**Results and Discussion**		
**15**	Provide published estimates in a file format from which data can be efficiently extracted.	Main text (Methods, Results and Discussion), Appendix
**16**	Report a quantitative measure of the uncertainty of the estimates (e.g. uncertainty intervals).	Main text (Methods)
**17**	Interpret results in light of existing evidence. If updating a previous set of estimates, describe the reasons for changes in estimates.	Main text (Methods, Results and Discussion)
**18**	Discuss limitations of the estimates. Include a discussion of any modelling assumptions or data limitations that affect interpretation of the estimates.	Main text (Methods and Discussion)

This checklist should be used in conjunction with the GATHER statement and Explanation and Elaboration document, found on gather-statement.org.2

Section 2. Methods Appendix

This appendix provides further methodological detail for "The burden of diseases and risk factors in Bangladesh, 1990-2019: a systematic analysis for the Global Burden of Disease Study 2019"

Portions of this appendix have been reproduced or adapted from the Appendix-1, Vos T, Lim SS, Abbafati C, et al. Global burden of 369 diseases and injuries in 204 countries and territories, 1990–2019: a systematic analysis for the Global Burden of Disease Study 2019. *The Lancet* 2020; **396**(10258): 1204-22.[1]

Systematic literature review and identification and selection of studies and data sources

GBD conducts a large number of systematic reviews of published literature using search strings developed by researchers and in conjunction with GBD collaborators on an annual basis. PubMed is the largest source. Details of the search terms are available in the Appendix-1, GBD 2019 capstone paper (Vos T, Lim SS, Abbafati C, et al. Global burden of 369 diseases and injuries in 204 countries and territories, 1990–2019: a systematic analysis for the Global Burden of Disease Study 2019. *The Lancet* 2020; **396**(10258): 1204-22.).[1] In addition, we systematically search for unpublished data on Ministry of Health websites, surveillance systems, case notification systems (e.g., infectious diseases), disease registries (e.g., cancer, congenital birth defects, chronic kidney disease), and hospital data (inpatient and outpatient, ICD-coded, primarily for non-fatal models). GBD 2019 synthesises a large and growing number of data input sources including surveys, censuses, vital statistics, and other health-related data sources. All data and sources are shared with GBD Scientific Council, experts, partners, and country collaborators for validation and requesting additional sources if available. Data are standardised and pooled into a single database used to generate cause-specific mortality estimates by age, sex, year, and geography.

Citations for specific GBD components, causes and risks, and locations can be found through the GBD Data Input Sources Tool in GHDx: http://ghdx.healthdata.org/gbd-2019/data-input-sources. This tool allows users to view and access GHDx records for input sources and export a comma-separated value (CSV) file that includes metadata, citations, and information about where the data were used in GBD. Users can select data input sources by GBD component (e.g., causes of death, non-fatal outcomes, covariates, mortality and population, risk factors), and specific location, cause, risk, covariate, or impairment, and then view and access the Global Health Data Exchange (GHDx) records for each data source used by GBD.

DisMod-MR model development and validation

The DisMod-MR tool evaluated and pooled all available data, adjusted data for systematic bias associated with methods that varied from the reference and produced estimates by world regions with UIs by using Bayesian statistical methods. In cross-validation tests, the log rates specification worked as well or better than the negative binomial specification. The sequence of estimation occurs at five levels: global, super-region, region, country and, where applicable, subnational location.

The super-region priors are generated at the global level with mixed-effects, nonlinear regression by using all available data; the super-region fit, in turn, informs the region fit, and so on down the cascade. The wrapper gives analysts the choice to branch the cascade in terms of time and sex at different levels depending on data density. The default used in most models is to branch by sex after the global fit but to retain all years of data until the lowest level in the cascade is reached. The computational engine is limited to three levels of random effects; we differentiate estimates at the super-region, region and country level. The coefficients for country covariates are re-estimated at each level of the cascade. For a given location, country coefficients are calculated by using both data and prior information available for that location. In the absence of data, the coefficient of its parent location is used to utilise the predictive power of our covariates in data-sparse situations. To determine the robustness of the models, we included the option again to have random effects on cause-specific mortality rates (CSMR) and EMR. Based on simulation testing we found that coverage improved, and errors reduced when passing down priors with a wider setting of minimum coefficient of variation (which determines the uncertainty around priors and hence how ‘informative’ the priors are) than had generally been used in past GBD iterations. We settled on a default value of 0.8.

We carried out simulation testing using DisMod-MR 2.1 based on an internally consistent set of 15,601 data points for prevalence, incidence, excess mortality, CSMR, and remission. We aimed to test what level of minimum CV would create the best fit based on the following three performance statistics:
(1) Coverage, ie, the proportion of data point mean values that fall between the 2.5th and 97.5th percentile of the draws of the fit values.
(2) Root mean square error: the square root of the mean of the squares of the difference between data point mean values and the mean fit value; and
(3) Bias: the difference between the mean fit value and the data point mean value.

We created different datasets culling the initial complete set with values at every age, sex, and location to more realistic data sparsity scenarios for analysis.

A first strategy was to randomly reduce the dataset to 10%, 5%, 2.5%, 1%, and 0.5% of the original data points. Initial results indicated little variation between the data samples culled to 10%, 5%, 2.5%, and 1%. The 0.5% culled dataset was an exception with markedly worse performance statistics, particularly with regard to bias and RMSE. We conducted further studies using the datasets culled to 10%, 5%, and 0.5%.

For some causes such as HIV/AIDS or measles, disease-specific natural history models have been used for which the underlying three state model in DisMod-MR 2.1 (susceptible, cases, dead) is insufficient to capture the complexity of a disease process. For some diseases with a range of sequelae differentiated by severity, such as COPD or diabetes mellitus, DisMod-MR 2.1 was used to meta-analyse the data on overall prevalence with separate DisMod-MR 2.1 models of the proportions of cases with different severity levels or sequelae. Likewise, DisMod-MR 2.1 was used to meta-analyse data on the proportions of liver cancer and cirrhosis due to underlying aetiologies such as hepatitis B, hepatitis C, and alcohol use. The GBD modelling software has been previously validated by comparing projected estimates

with observed data for 2014-16. Model performance was assessed using mean error and root-mean-squared error. The model performed better than the most commonly used approach, i.e. the Lee-Carter method.[1]

TMREL

Associations between potential risk factors and disease burden were evaluated using the GBD comparative risk assessment framework, in which estimates of theoretical minimum risk exposure level (TMREL),[1] risk exposure, relative disease risk, and attributable burden are generated for 87 risk factors. TMREL refers to the level of exposure that is theoretically possible and associated with the lowest disease risk. Population attributable fraction—the proportional reduction in disease burden that would occur if a risk were reduced to the TMREL—is multiplied by the total number of deaths or YLLs to compute attributable burden, the proportion of health burden attributable to the risk factor.[1]

Section 3. List of Figures

Figure S1. Total fertility rate and under-5 mortality during 1990 and 2019: comparison between Bangladesh and south Asian countries

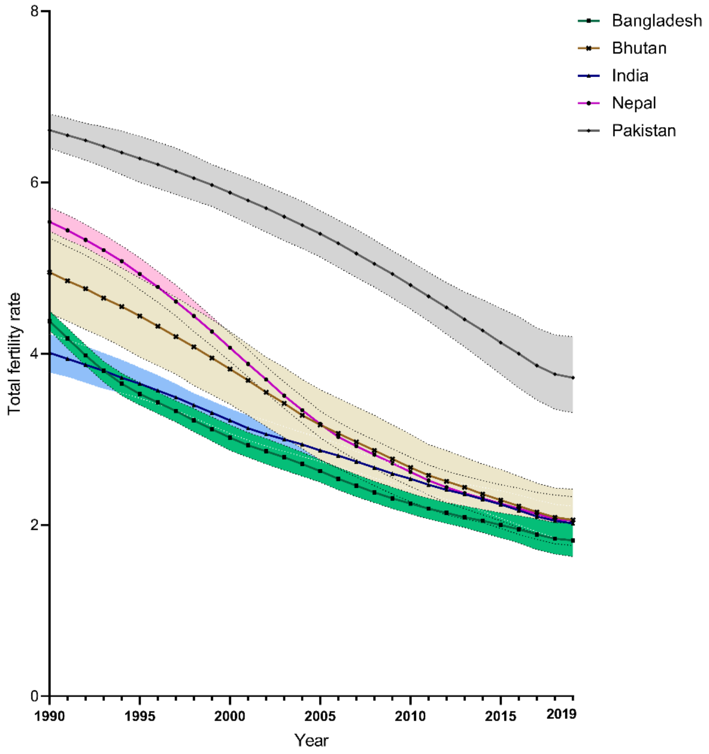

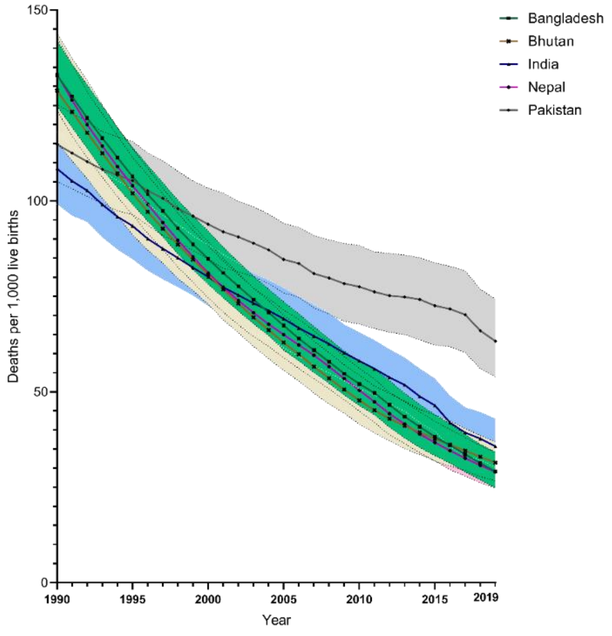

Figure S2A. Ranking of the leading causes of age-standardised rates of disability-adjusted life-years (DALYs) in Bangladesh compared to other south Asian countries, 1990.

	**Bangladesh**	Bhutan	India	Nepal	Pakistan
Neonatal disorders	1	1	2	3	1
Diarrheal diseases	2	4	1	2	2
Lower respiratory infect	3	3	3	1	4
Tuberculosis	4	5	4	4	3
Stroke	5	9	7	9	7
Protein-energy malnutrition	6	25	8	10	23
Ischemic heart disease	7	6	5	8	6
COPD	8	7	6	5	8
Malaria	9	2	10	105	27
Drowning	10	31	29	15	43
Cirrhosis	11	11	18	11	9
Congenital defects	12	10	14	14	11
Tetanus	13	17	19	6	12
Maternal disorders	14	16	21	13	16
Measles	15	8	11	7	5
Typhoid & paratyph	16	28	17	23	15
Asthma	17	14	16	12	20
Diabetes	18	23	22	31	14
Depressive disorders	19	20	25	20	19
Low back pain	20	18	20	18	22
Dietary iron deficiency	21	12	13	22	13
Whooping cough	22	32	34	24	34
Road injuries	23	13	9	17	21
Other musculoskeletal	24	29	32	32	32
Self-harm	25	35	12	19	33

Figure S2B. Ranking of the leading causes of age-standardised rates of disability-adjusted life-years (DALYs) in Bangladesh compared to other south Asian countries, 2010.

	Bangladesh	Bhutan	India	Nepal	Pakistan
Neonatal disorders	1	1	1	1	1
Stroke	2	5	7	5	4
Ischemic heart disease	3	2	2	4	2
Lower respiratory infect	4	4	4	3	6
COPD	5	3	5	2	7
Tuberculosis	6	11	6	7	5
Diarrheal diseases	7	6	3	6	3
Congenital defects	8	9	14	13	13
Diabetes	9	10	10	14	8
Depressive disorders	10	15	17	9	18
Drowning	11	37	32	28	75
Cirrhosis	12	8	12	8	9
Other musculoskeletal	13	17	15	17	26
Low back pain	14	13	18	10	19
Road injuries	15	12	8	19	12
Dietary iron deficiency	16	7	9	15	11
Headache disorders	17	19	21	18	23
Typhoid & paratyph	18	23	24	30	17
Age-related hearing loss	19	20	23	25	25
Blindness and vision loss	20	27	20	22	21
Other malignant neoplasms	21	48	57	23	31
Chronic kidney disease	22	14	16	20	10
Gynecological diseases	23	24	27	29	30
Asthma	24	21	19	11	24
Protein-energy malnutrition	25	55	29	27	35

Figure S2C. Ranking of the leading causes of age-standardised rates of disability-adjusted life-years (DALYs) in Bangladesh compared to other south Asian countries, 2019.

	**Bangladesh**	Bhutan	India	Nepal	Pakistan
Neonatal disorders	1	1	1	2	1
Stroke	2	4	6	4	3
Ischemic heart disease	3	2	2	3	2
Lower respiratory infect	4	5	5	5	5
COPD	5	3	3	1	6
Diabetes	6	7	9	11	8
Diarrheal diseases	7	9	4	8	4
Other musculoskeletal	8	15	12	15	18
Depressive disorders	9	14	16	9	15
Congenital defects	10	11	15	19	14
Low back pain	11	10	19	10	16
Tuberculosis	12	18	7	6	7
Cirrhosis	13	8	13	7	10
Headache disorders	14	17	18	16	19
Road injuries	15	13	8	17	11
Age-related hearing loss	16	19	20	22	22
Dietary iron deficiency	17	6	10	18	12
Blindness and vision loss	18	28	21	23	21
Other malignant neoplasms	19	45	54	21	27
Gynecological diseases	20	21	23	24	31
Typhoid & paratyph	21	25	25	33	17
Chronic kidney disease	22	12	17	14	9
Drowning	23	46	32	30	77
Anxiety disorders	24	24	28	27	34
Alzheimer's disease	25	26	31	31	37

Figure S3. Changes in ranking of causes of under-5 mortality in Bangladesh between 1990 and 2019

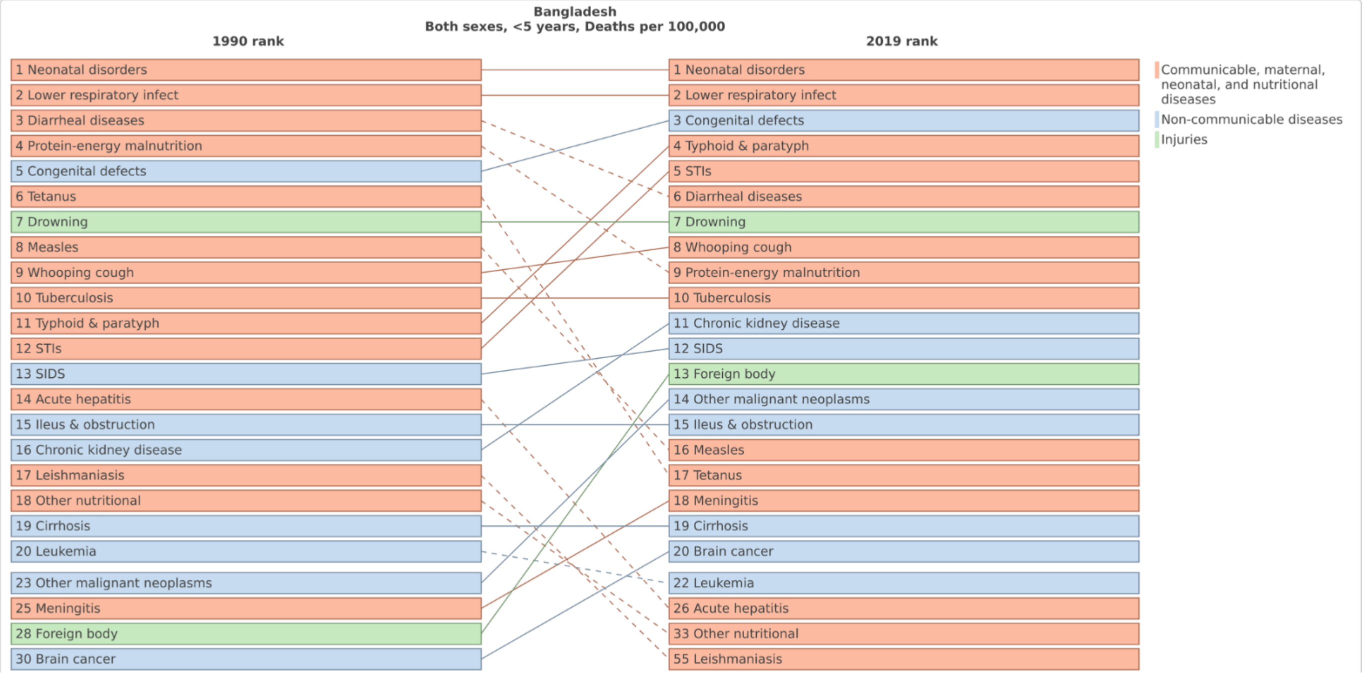

Figure S4. Population density of Bangladesh according to districts (census 2011)

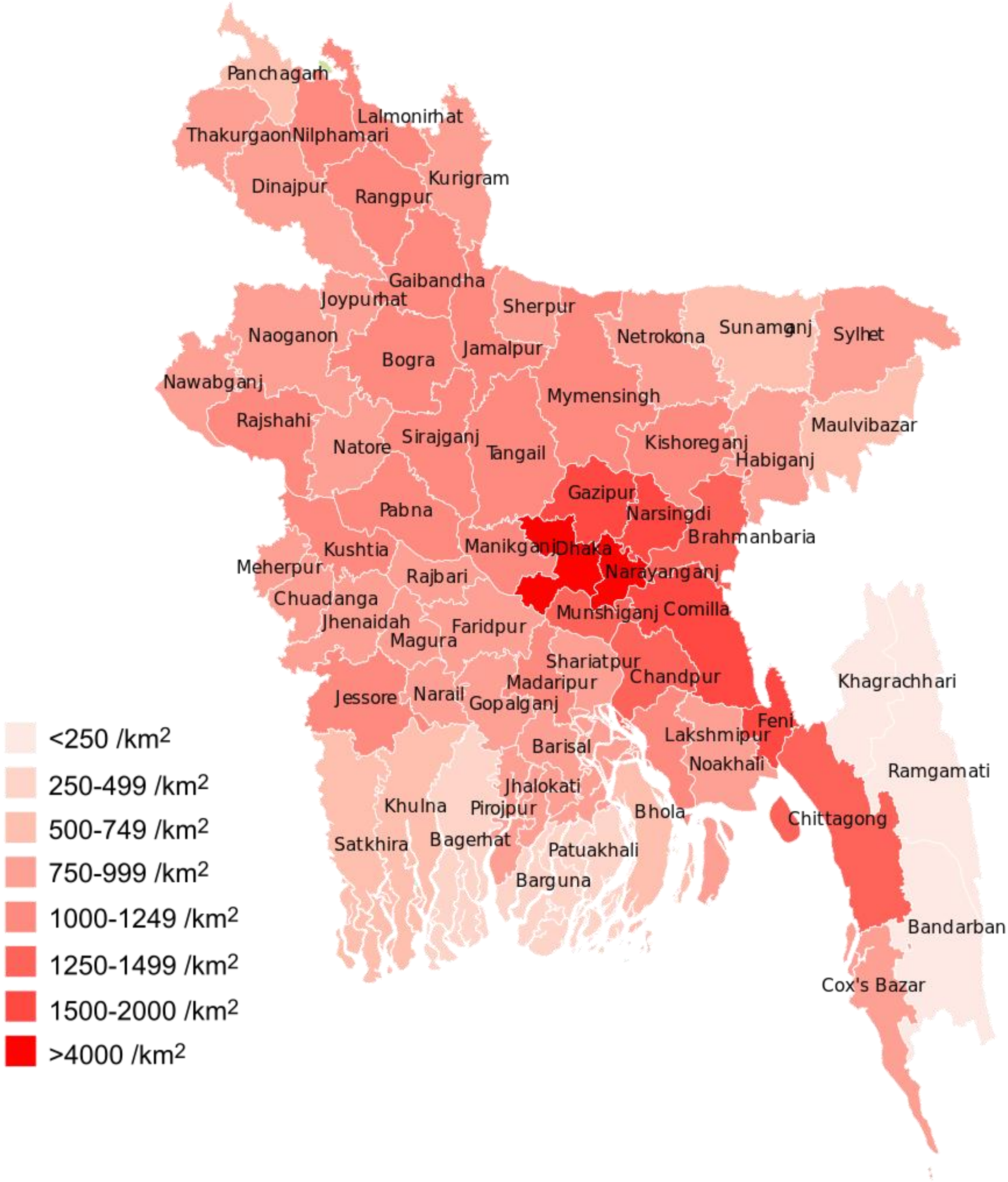

(Source: commons.wikimedia.org)

Figure S5. Total Fertility Rate in Bangladesh, Bhutan, India, Nepal, and Pakistan 1990-2019

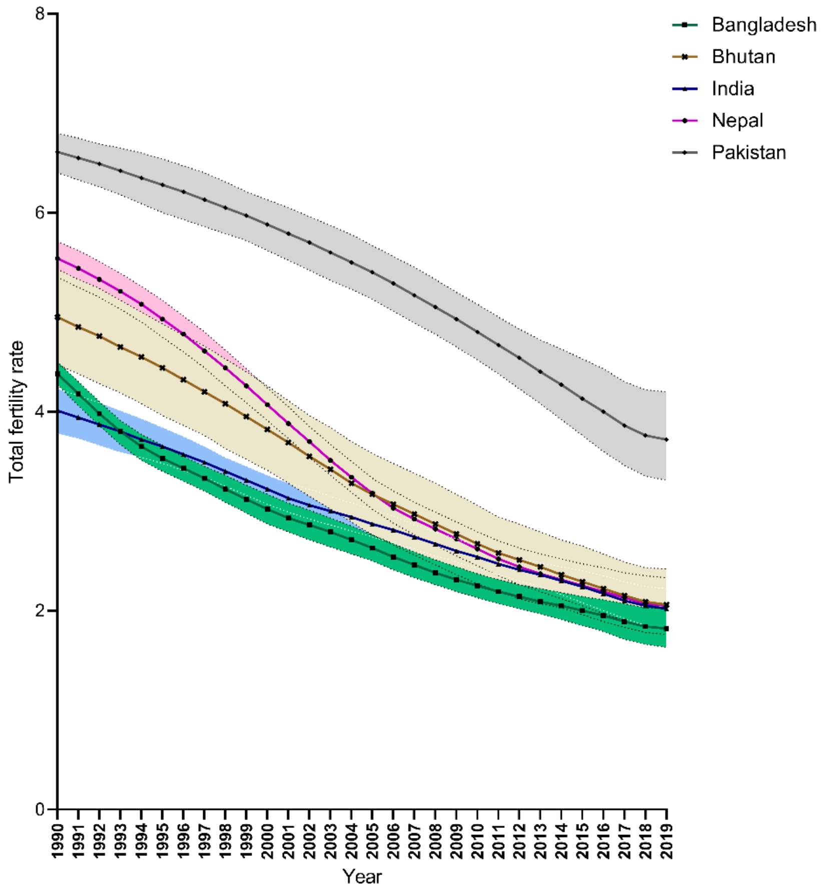

Figure S6. Deaths per 1000 live births in Bangladesh, Bhutan, India, Nepal, and Pakistan 1990-2019

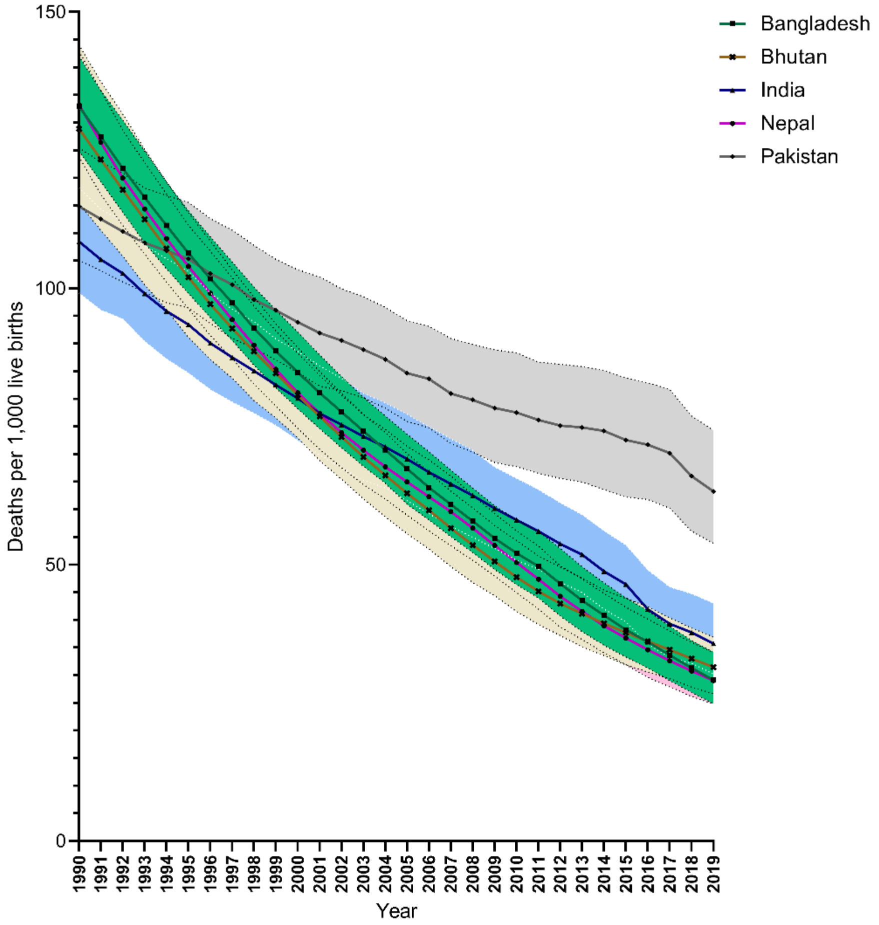

Figure S7. Life expectancy at birth in Bangladesh, Bhutan, India, Nepal, and Pakistan 1990-2019

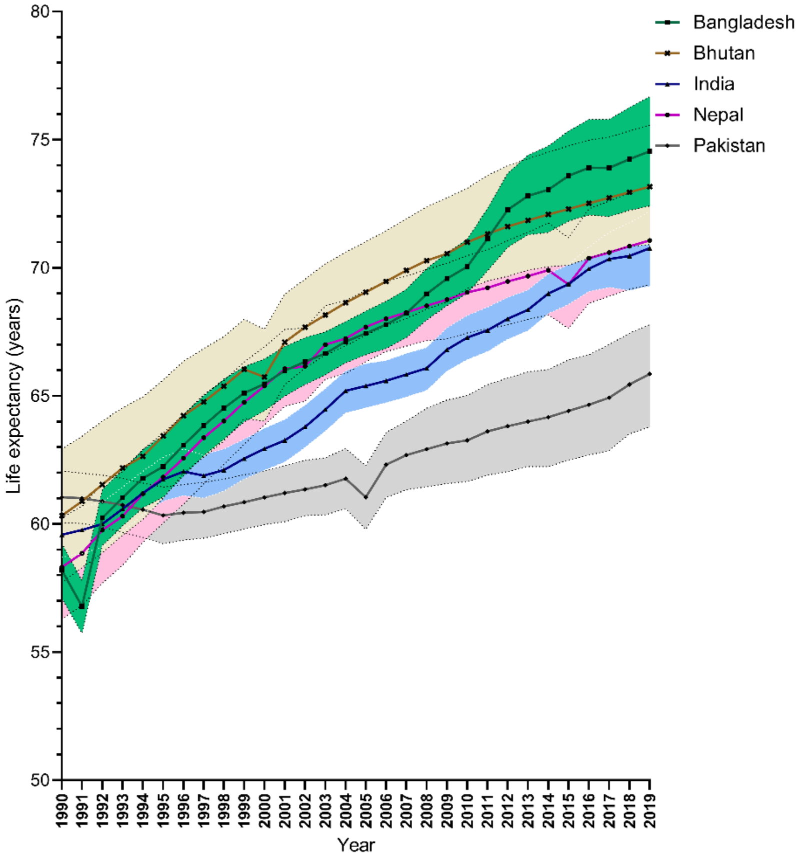

Figure S8. Healthy life expectancy in Bangladesh, Bhutan, India, Nepal, and Pakistan 1990-2019

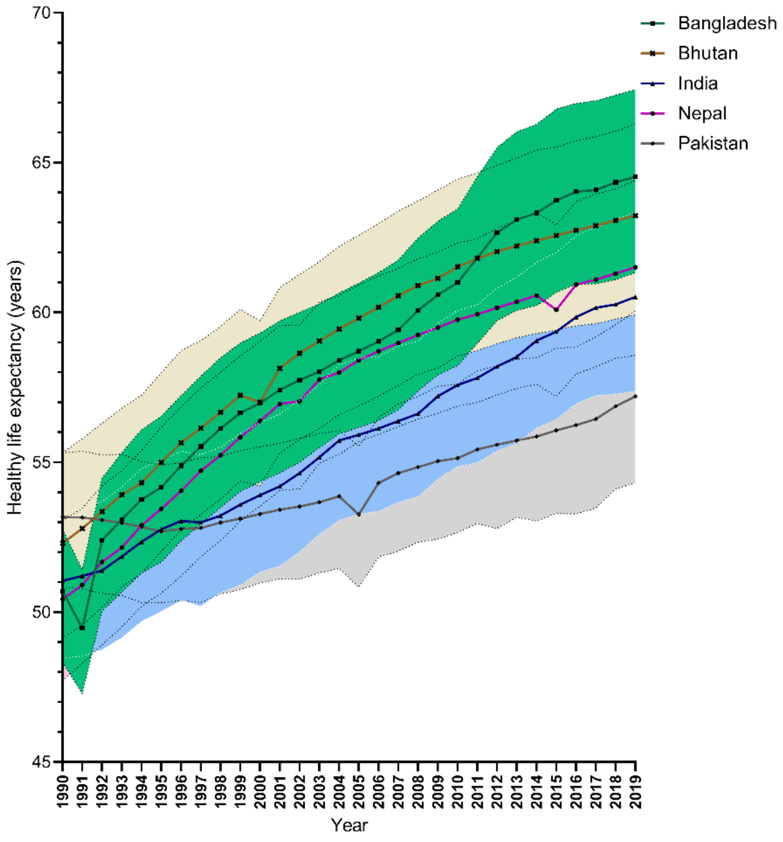

Figure S9. Changes in age-standardised prevalence of diseases and injuries (level 1) in Bangladesh from 1990-2019

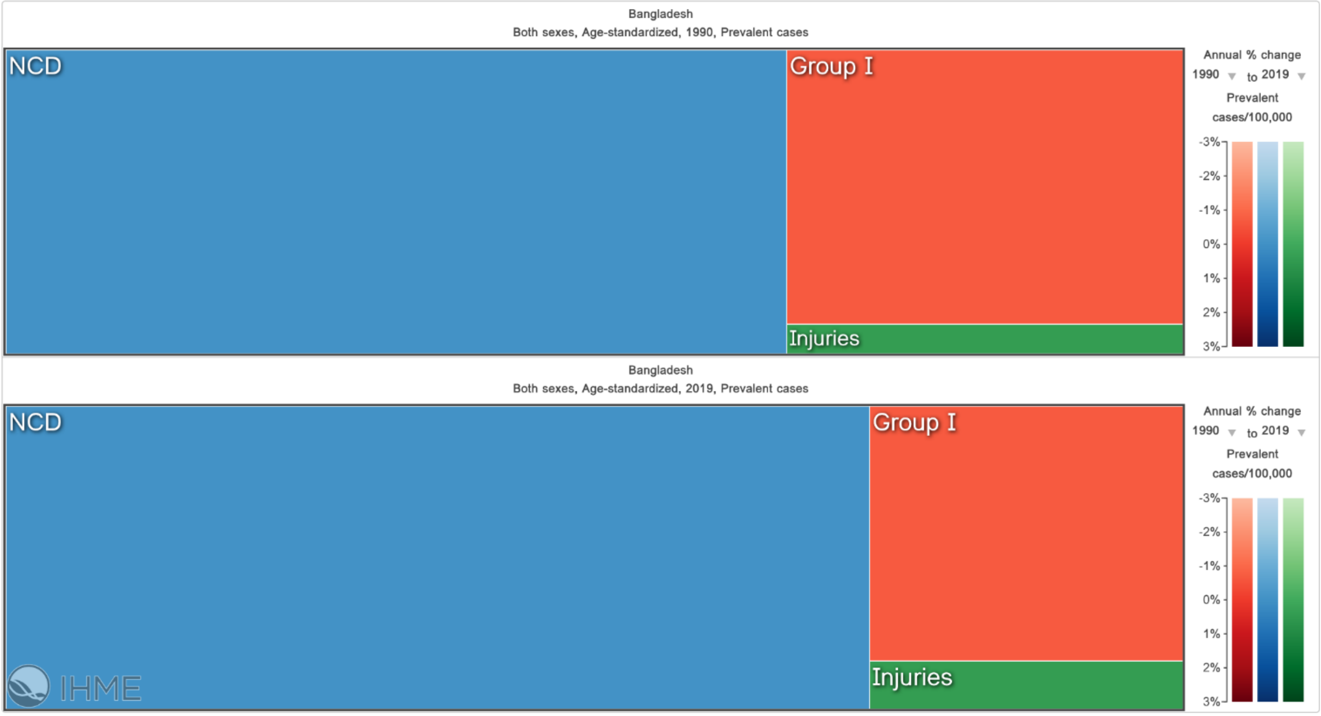

Figure S10. Changes in age-standardised prevalence of diseases and injuries (level 2) in Bangladesh from 1990-2019

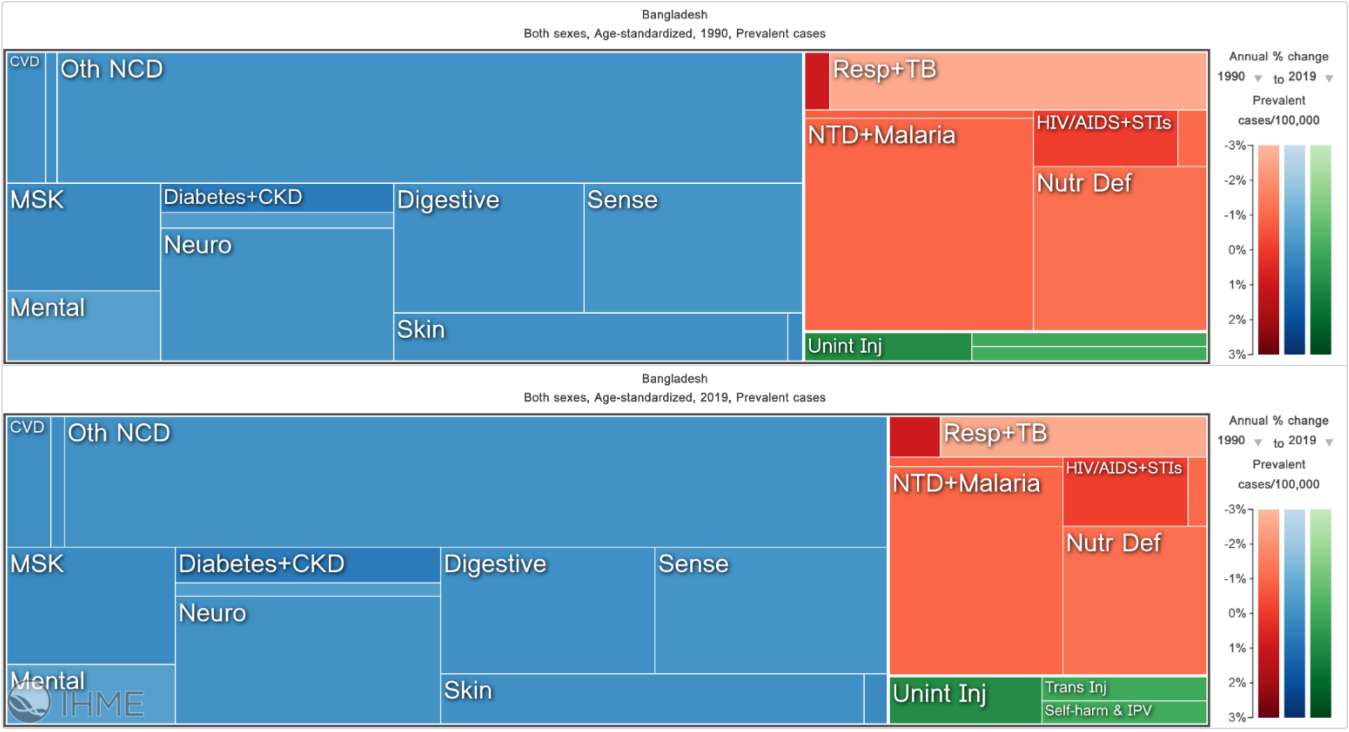

Section 4. Tables

Table S1. Total fertility rate, life expectancy at birth (years), healthy life expectancy at birth (years), and under-5 mortality (deaths per 1000 live births) during 1990 and 2019 in Bangladesh

Year	Total fertility rate	Life expectancy at birth (years)	Healthy life expectancy at birth (years)	Under-5 mortality (deaths per 1000 livebirths)
1990	4.38 (4.27-4.50)	58.19 (57.10-59.23)	50.70 (48.33-52.76)	132.90 (124.80-141.99)
1991	4.18 (4.06-4.30)	56.79 (55.74-57.80)	49.47 (47.28-51.42)	127.40 (119.31-135.84)
1992	3.98 (3.86-4.10)	60.24 (59.16-61.34)	52.40 (50.04-54.47)	121.74 (113.79-130.46)
1993	3.80 (3.67-3.91)	61.03 (59.93-62.12)	53.10 (50.67-55.33)	116.46 (108.24-124.88)
1994	3.65 (3.51-3.77)	61.79 (60.64-62.90)	53.76 (51.31-56.09)	111.40 (103.55-119.23)
1995	3.53 (3.40-3.66)	62.24 (61.05-63.45)	54.17 (51.67-56.54)	106.37 (99.09-114.05)
1996	3.43 (3.30-3.55)	63.08 (61.88-64.28)	54.89 (52.38-57.23)	101.75 (94.72-109.33)
1997	3.33 (3.20-3.45)	63.84 (62.67-65.00)	55.53 (52.95-57.88)	97.40 (90.57-104.77)
1998	3.22 (3.09-3.36)	64.52 (63.31-65.66)	56.13 (53.49-58.49)	92.81 (86.13-100.27)
1999	3.12 (2.98-3.26)	65.11 (63.99-66.14)	56.65 (54.03-58.98)	88.68 (82.02-96.11)

2000	3.02 (2.87-3.17)	65.47 (64.42-66.44)	56.98 (54.35-59.32)	84.83 (78.20-92.13)
2001	2.93 (2.79-3.08)	65.99 (64.99-66.93)	57.42 (54.65-59.72)	81.12 (74.64-87.92)
2002	2.86 (2.71-3.01)	66.34 (65.44-67.27)	57.74 (55.00-60.01)	77.67 (71.16-84.09)
2003	2.79 (2.64-2.93)	66.66 (65.82-67.50)	58.03 (55.49-60.29)	74.19 (67.83-80.30)
2004	2.71 (2.57-2.85)	67.12 (66.29-67.90)	58.41 (55.95-60.65)	70.79 (64.78-76.90)
2005	2.63 (2.50-2.76)	67.44 (66.61-68.31)	58.71 (56.16-60.98)	67.39 (60.97-73.63)
2006	2.54 (2.41-2.67)	67.79 (66.85-68.70)	59.04 (56.39-61.33)	63.98 (58.13-70.46)
2007	2.46 (2.33-2.59)	68.22 (67.28-69.16)	59.42 (56.76-61.75)	60.91 (55.10-67.13)
2008	2.38 (2.26-2.51)	68.98 (67.97-69.97)	60.07 (57.38-62.49)	57.90 (52.21-63.85)
2009	2.31 (2.19-2.44)	69.58 (68.52-70.58)	60.59 (57.92-63.05)	54.80 (49.14-60.69)
2010	2.25 (2.13-2.37)	70.04 (69.01-71.12)	61.00 (58.23-63.45)	52.11 (46.41-58.04)
2011	2.19 (2.07-2.31)	71.14 (69.89-72.32)	61.83 (58.97-64.53)	49.70 (44.00-56.02)
2012	2.14 (2.02-2.26)	72.26 (70.80-73.69)	62.66 (59.72-65.50)	46.59 (40.75-52.95)
2013	2.09 (1.97-2.22)	72.81 (71.29-74.40)	63.10 (60.07-66.03)	43.56 (37.91-49.64)

2014	2.05 (1.91-2.18)	73.05 (71.38-74.75)	63.32 (60.23-66.28)	40.85 (35.45-46.72)
2015	2.00 (1.85-2.14)	73.59 (71.82-75.34)	63.74 (60.67-66.79)	38.25 (33.29-44.01)
2016	1.95 (1.79-2.11)	73.91 (72.06-75.79)	64.03 (60.95-66.98)	36.04 (31.31-41.68)
2017	1.89 (1.71-2.07)	73.90 (72.00-75.80)	64.09 (60.95-67.07)	33.63 (29.04-38.89)
2018	1.84 (1.66-2.03)	74.24 (72.25-76.26)	64.34 (61.10-67.26)	31.31 (26.92-36.29)
2019	1.82 (1.63-2.02)	74.55 (72.41-76.68)	64.53 (61.33-67.44)	29.13 (24.85-34.20)

Table S2. Prevalence of diseases and injuries in Bangladesh in 1990 and 2019 (Level 1 Causes)

Age	Diseases	Metric	Year	Prevalence	Upper	Lower
All ages	NCDs	Number	1990	95499097.11	97249607.58	93738238.62
All ages	NCDs	Percent	1990	0.89	0.91	0.88
All ages	NCDs	Rate	1990	87570.33	89175.50	85955.66
All ages	NCDs	Number	2019	145022380.05	146801210.27	143197334.96
All ages	NCDs	Percent	2019	0.94	0.95	0.93
All ages	NCDs	Rate	2019	91060.23	92177.16	89914.27
Age-standardized	NCDs	Percent	1990	0.93	0.94	0.92
Age-standardized	NCDs	Rate	1990	91326.97	92411.04	90215.63
Age-standardized	NCDs	Percent	2019	0.94	0.95	0.93
Age-standardized	NCDs	Rate	2019	90985.00	92105.38	89838.61
All ages	Injuries	Number	1990	13109784.81	13902736.17	12369419.73
All ages	Injuries	Percent	1990	0.12	0.13	0.12
All ages	Injuries	Rate	1990	12021.35	12748.47	11342.45
All ages	Injuries	Number	2019	28933877.02	30739391.30	27255518.63
All ages	Injuries	Percent	2019	0.19	0.20	0.18
All ages	Injuries	Rate	2019	18167.72	19301.41	17113.87
Age-standardized	Injuries	Percent	1990	0.17	0.18	0.16
Age-standardized	Injuries	Rate	1990	16428.29	17314.33	15610.18
Age-standardized	Injuries	Percent	2019	0.19	0.20	0.18
Age-standardized	Injuries	Rate	2019	18715.65	19815.05	17681.06
All ages	CMNNDs	Number	2019	104864051.25	110702060.16	99228030.14
All ages	CMNNDs	Percent	2019	0.68	0.71	0.64
All ages	CMNNDs	Rate	2019	65844.63	69510.34	62305.74
Age-standardized	CMNNDs	Percent	1990	0.81	0.83	0.79
Age-standardized	CMNNDs	Rate	1990	80060.97	81957.87	78088.48
Age-standardized	CMNNDs	Percent	2019	0.68	0.71	0.64
Age-standardized	CMNNDs	Rate	2019	65989.35	69525.94	62502.72
All ages	CMNNDs	Number	1990	88600078.66	90815389.30	86171091.05
All ages	CMNNDs	Percent	1990	0.83	0.85	0.81
All ages	CMNNDs	Rate	1990	81244.10	83275.48	79016.77

NCDs Non-communicable diseases; CMNNDs Communicable, maternal, neonatal, and nutritional diseases; Rate per 100,000 individuals

Table S3. Prevalence of diseases and injuries in Bangladesh in 1990 and 2019 (Level 2 Causes)

Age	Diseases	Metric	Prevalence	Upper	Lower
All ages	Digestive diseases	Number	1.05	1.10	1.00
All ages	Digestive diseases	Percent	0.41	0.45	0.38
All ages	Digestive diseases	Rate	0.40	0.44	0.37
Age-standardized	Digestive diseases	Percent	0.02	0.04	0.00
Age-standardized	Digestive diseases	Rate	0.01	0.02	-0.01
All ages	HIV/AIDS and sexually transmitted infections	Number	1.00	1.09	0.92
All ages	HIV/AIDS and sexually transmitted infections	Percent	0.38	0.44	0.33
All ages	HIV/AIDS and sexually transmitted infections	Rate	0.37	0.43	0.32
Age-standardized	HIV/AIDS and sexually transmitted infections	Percent	-0.01	0.03	-0.04
Age-standardized	HIV/AIDS and sexually transmitted infections	Rate	-0.02	0.01	-0.05
All ages	Respiratory infections and tuberculosis	Number	-0.33	-0.26	-0.40
All ages	Respiratory infections and tuberculosis	Percent	-0.54	-0.49	-0.59
All ages	Respiratory infections and tuberculosis	Rate	-0.54	-0.49	-0.59
Age-standardized	Respiratory infections and tuberculosis	Percent	-0.52	-0.48	-0.56
Age-standardized	Respiratory infections and tuberculosis	Rate	-0.53	-0.49	-0.57
All ages	Enteric infections	Number	0.16	0.29	0.05
All ages	Enteric infections	Percent	-0.20	-0.11	-0.28
All ages	Enteric infections	Rate	-0.21	-0.12	-0.28
Age-standardized	Enteric infections	Percent	-0.19	-0.11	-0.25
Age-standardized	Enteric infections	Rate	-0.20	-0.13	-0.26
All ages	Other infectious diseases	Number	-0.09	0.01	-0.18
All ages	Other infectious diseases	Percent	-0.37	-0.31	-0.43
All ages	Other infectious diseases	Rate	-0.38	-0.31	-0.44
Age-standardized	Other infectious diseases	Percent	-0.25	-0.18	-0.31
Age-standardized	Other infectious diseases	Rate	-0.26	-0.19	-0.32
All ages	Chronic respiratory diseases	Number	0.92	1.02	0.83
All ages	Chronic respiratory diseases	Percent	0.32	0.39	0.26
All ages	Chronic respiratory diseases	Rate	0.31	0.38	0.25
Age-standardized	Chronic respiratory diseases	Percent	-0.10	-0.06	-0.13

Age-standardized	Chronic respiratory diseases	Rate	-0.11	-0.08	-0.14
All ages	Nutritional deficiencies	Number	0.02	0.11	-0.08
All ages	Nutritional deficiencies	Percent	-0.30	-0.23	-0.36
All ages	Nutritional deficiencies	Rate	-0.30	-0.24	-0.37
Age-standardized	Nutritional deficiencies	Percent	-0.27	-0.22	-0.33
Age-standardized	Nutritional deficiencies	Rate	-0.28	-0.22	-0.34
All ages	Neurological disorders	Number	0.72	0.78	0.67
All ages	Neurological disorders	Percent	0.19	0.23	0.15
All ages	Neurological disorders	Rate	0.18	0.22	0.14
Age-standardized	Neurological disorders	Percent	0.02	0.02	0.01
Age-standardized	Neurological disorders	Rate	0.00	0.01	-0.01
All ages	Neglected tropical diseases and malaria	Number	0.06	0.26	-0.12
All ages	Neglected tropical diseases and malaria	Percent	-0.27	-0.14	-0.39
All ages	Neglected tropical diseases and malaria	Rate	-0.27	-0.14	-0.39
Age-standardized	Neglected tropical diseases and malaria	Percent	-0.22	-0.08	-0.35
Age-standardized	Neglected tropical diseases and malaria	Rate	-0.23	-0.09	-0.36
All ages	Cardiovascular diseases	Number	1.64	1.71	1.57
All ages	Cardiovascular diseases	Percent	0.82	0.87	0.78
All ages	Cardiovascular diseases	Rate	0.81	0.86	0.76
Age-standardized	Cardiovascular diseases	Percent	0.04	0.07	0.02
Age-standardized	Cardiovascular diseases	Rate	0.03	0.05	0.00
All ages	Skin and subcutaneous diseases	Number	0.53	0.56	0.49
All ages	Skin and subcutaneous diseases	Percent	0.06	0.08	0.03
All ages	Skin and subcutaneous diseases	Rate	0.05	0.07	0.02
Age-standardized	Skin and subcutaneous diseases	Percent	0.04	0.05	0.02
Age-standardized	Skin and subcutaneous diseases	Rate	0.02	0.03	0.01
All ages	Mental disorders	Number	0.45	0.55	0.36
All ages	Mental disorders	Percent	0.00	0.07	-0.06
All ages	Mental disorders	Rate	-0.01	0.06	-0.07
Age-standardized	Mental disorders	Percent	-0.13	-0.08	-0.17
Age-standardized	Mental disorders	Rate	-0.14	-0.10	-0.18
All ages	Neoplasms	Number	1.08	1.23	0.95
All ages	Neoplasms	Percent	0.44	0.54	0.34
All ages	Neoplasms	Rate	0.43	0.53	0.33

Age-standardized	Neoplasms	Percent	0.04	0.08	0.00
Age-standardized	Neoplasms	Rate	0.03	0.07	-0.01
All ages	Other non-communicable diseases	Number	0.53	0.56	0.50
All ages	Other non-communicable diseases	Percent	0.06	0.08	0.04
All ages	Other non-communicable diseases	Rate	0.05	0.07	0.03
Age-standardized	Other non-communicable diseases	Percent	0.02	0.03	0.01
Age-standardized	Other non-communicable diseases	Rate	0.00	0.02	-0.01
All ages	Maternal and neonatal disorders	Number	0.67	0.92	0.45
All ages	Maternal and neonatal disorders	Percent	0.15	0.33	0.00
All ages	Maternal and neonatal disorders	Rate	0.14	0.31	-0.01
Age-standardized	Maternal and neonatal disorders	Percent	0.34	0.54	0.15
Age-standardized	Maternal and neonatal disorders	Rate	0.32	0.52	0.14
All ages	Sense organ diseases	Number	1.26	1.33	1.20
All ages	Sense organ diseases	Percent	0.56	0.61	0.52
All ages	Sense organ diseases	Rate	0.55	0.59	0.51
Age-standardized	Sense organ diseases	Percent	-0.01	0.01	-0.04
Age-standardized	Sense organ diseases	Rate	-0.03	0.00	-0.05
All ages	Unintentional injuries	Number	1.49	1.58	1.41
All ages	Unintentional injuries	Percent	0.72	0.79	0.66
All ages	Unintentional injuries	Rate	0.71	0.77	0.65
Age-standardized	Unintentional injuries	Percent	0.34	0.40	0.29
Age-standardized	Unintentional injuries	Rate	0.32	0.38	0.27
All ages	Musculoskeletal disorders	Number	1.36	1.43	1.27
All ages	Musculoskeletal disorders	Percent	0.63	0.68	0.57
All ages	Musculoskeletal disorders	Rate	0.61	0.67	0.55
Age-standardized	Musculoskeletal disorders	Percent	0.08	0.11	0.05
Age-standardized	Musculoskeletal disorders	Rate	0.07	0.09	0.03
All ages	Transport injuries	Number	1.26	1.30	1.21
All ages	Transport injuries	Percent	0.56	0.59	0.53
All ages	Transport injuries	Rate	0.55	0.58	0.52
Age-standardized	Transport injuries	Percent	0.04	0.06	0.02
Age-standardized	Transport injuries	Rate	0.02	0.04	0.01

All ages	Self-harm and interpersonal violence	Number	0.80	0.86	0.76
All ages	Self-harm and interpersonal violence	Percent	0.25	0.28	0.21
All ages	Self-harm and interpersonal violence	Rate	0.24	0.27	0.20
Age-standardized	Self-harm and interpersonal violence	Percent	0.01	0.02	0.00
Age-standardized	Self-harm and interpersonal violence	Rate	0.00	0.01	-0.01
All ages	Substance use disorders	Number	1.02	1.18	0.87
All ages	Substance use disorders	Percent	0.40	0.50	0.30
All ages	Substance use disorders	Rate	0.38	0.49	0.28
Age-standardized	Substance use disorders	Percent	0.08	0.15	0.01
Age-standardized	Substance use disorders	Rate	0.07	0.14	-0.01
All ages	Diabetes and kidney diseases	Number	1.80	1.88	1.71
All ages	Diabetes and kidney diseases	Percent	0.93	0.99	0.87
All ages	Diabetes and kidney diseases	Rate	0.92	0.97	0.86
Age-standardized	Diabetes and kidney diseases	Percent	0.25	0.28	0.21
Age-standardized	Diabetes and kidney diseases	Rate	0.23	0.26	0.20

Rate per 100,000 individuals

Table S4. Annual rate of changes in prevalence of diseases and injuries in Bangladesh from 1990 and 2019

Age	Diseases	Metric	Changes in Prevalence	Upper	Lower
All ages	NCDs	Number	0.52	0.53	0.51
All ages	NCDs	Percent	0.05	0.06	0.04
All ages	NCDs	Rate	0.04	0.05	0.03
Age-standardized	NCDs	Percent	0.01	0.01	0.00
Age-standardized	NCDs	Rate	0.00	0.00	-0.01
All ages	CMNNDs	Number	0.18	0.26	0.11
All ages	CMNNDs	Percent	-0.18	-0.13	-0.23
All ages	CMNNDs	Rate	-0.19	-0.14	-0.24
Age-standardized	CMNNDs	Percent	-0.16	-0.12	-0.21
Age-standardized	CMNNDs	Rate	-0.18	-0.12	-0.22
Age-standardized	Injuries	Percent	0.15	0.18	0.13
Age-standardized	Injuries	Rate	0.14	0.17	0.12
All ages	Injuries	Number	1.21	1.26	1.16
All ages	Injuries	Percent	0.52	0.56	0.49
All ages	Injuries	Rate	0.51	0.55	0.48

NCDs Non-communicable diseases; CMNNDs Communicable, maternal, neonatal, and nutritional diseases; Rate per 100,000 individuals

Table S5. Under 5 mortality (deaths per 1,000 live births) during 1990 and 2019: comparison between Bangladesh and South Asian countries

Years	Bangladesh	Bhutan	India	Nepal	Pakistan
1990	132.90 (124.80-141.99)	128.88 (115.40-143.99)	108.49 (99.16-118.15)	133.22 (123.75-142.85)	114.82 (105.07-125.39)
1991	127.40 (119.31-135.84)	123.35 (110.53-137.43)	105.23 (96.12-114.31)	126.44 (117.01-135.84)	112.53 (103.22-122.91)
1992	121.74 (113.79-130.46)	117.84 (105.86-131.56)	102.73 (94.49-112.15)	119.97 (111.39-128.62)	110.32 (101.19-120.86)
1993	116.46 (108.24-124.88)	112.52 (100.66-125.16)	99.05 (90.47-107.78)	114.36 (106.22-122.79)	108.26 (99.28-118.14)
1994	111.40 (103.55-119.23)	107.22 (96.13-119.29)	95.88 (87.24-105.36)	109.01 (101.10-117.07)	106.78 (97.41-116.81)
1995	106.37 (99.09-114.05)	102.01 (91.15-113.72)	93.47 (84.72-103.08)	104.00 (96.32-111.42)	105.34 (96.47-115.56)
1996	101.75 (94.72-109.33)	97.15 (87.14-108.57)	90.08 (81.74-99.19)	99.12 (91.49-106.76)	102.67 (93.69-112.63)
1997	97.40 (90.57-104.77)	92.76 (83.75-103.37)	87.52 (79.45-96.23)	94.36 (87.30-101.91)	100.71 (90.94-110.54)
1998	92.81 (86.13-100.27)	88.60 (79.72-98.45)	85.10 (77.42-94.01)	89.72 (82.71-96.81)	97.96 (89.24-107.82)
1999	88.68 (82.02-96.11)	84.64 (76.58-93.58)	82.56 (75.23-90.88)	85.34 (78.69-92.34)	96.03 (87.06-105.28)
2000	84.83 (78.20-92.13)	80.73 (72.97-89.32)	80.10 (72.52-88.60)	81.12 (74.68-88.33)	93.92 (84.80-103.38)
2001	81.12 (74.64-87.92)	76.88 (68.82-84.97)	77.39 (69.87-85.99)	77.38 (70.89-84.22)	91.91 (82.33-102.08)
2002	77.67 (71.16-84.09)	73.12 (65.46-81.04)	75.35 (67.49-83.76)	73.93 (67.56-80.69)	90.57 (81.51-99.91)
2003	74.19 (67.83-80.30)	69.50 (61.93-77.15)	73.22 (65.30-81.01)	70.73 (64.49-77.17)	88.94 (80.20-98.51)
2004	70.79 (64.78-76.90)	66.18 (58.68-73.90)	71.29 (63.90-79.29)	67.72 (61.87-74.47)	87.19 (78.33-96.54)
2005	67.39 (60.97-73.63)	62.95 (55.62-70.16)	69.12 (61.60-77.23)	64.99 (58.98-71.46)	84.69 (75.95-94.16)
2006	63.98 (58.13-70.46)	59.86 (52.84-66.72)	66.76 (59.27-74.96)	62.29 (56.14-69.00)	83.64 (74.76-93.13)

2007	60.91 (55.10-67.13)	56.62 (49.67-63.31)	64.60 (56.96-72.79)	59.60 (53.45-66.08)	80.98 (71.92-90.97)
2008	57.90 (52.21-63.85)	53.55 (46.78-60.29)	62.53 (54.96-70.70)	56.62 (50.79-62.59)	79.83 (70.38-89.85)
2009	54.80 (49.14-60.69)	50.62 (44.41-57.09)	60.19 (52.94-67.69)	53.49 (47.96-59.29)	78.38 (68.51-88.91)
2010	52.11 (46.41-58.04)	47.76 (41.51-54.39)	58.11 (50.81-65.64)	50.40 (44.98-56.12)	77.53 (67.75-88.33)
2011	49.70 (44.00-56.02)	45.20 (39.22-51.57)	56.04 (49.18-63.57)	47.38 (41.99-53.36)	76.19 (66.55-86.64)
2012	46.59 (40.75-52.95)	43.00 (37.21-49.59)	53.78 (46.91-61.20)	44.28 (38.73-49.83)	75.20 (65.64-86.28)
2013	43.56 (37.91-49.64)	41.14 (35.15-47.60)	51.82 (44.98-59.05)	41.55 (36.49-47.44)	74.85 (65.00-85.87)
2014	40.85 (35.45-46.72)	39.38 (33.55-45.42)	48.82 (41.97-56.15)	38.96 (34.04-44.80)	74.21 (63.59-85.15)
2015	38.25 (33.29-44.01)	37.73 (31.90-43.93)	46.45 (39.68-53.56)	36.76 (31.92-42.30)	72.58 (62.27-83.79)
2016	36.04 (31.31-41.68)	36.15 (30.58-42.44)	41.95 (35.66-49.04)	34.62 (29.61-40.17)	71.73 (61.81-82.92)
2017	33.63 (29.04-38.89)	34.63 (29.43-40.39)	39.33 (33.19-45.98)	32.64 (27.94-38.05)	70.23 (60.25-81.72)
2018	31.31 (26.92-36.29)	33.01 (27.84-38.59)	37.77 (32.18-44.74)	30.76 (26.17-35.96)	66.02 (56.05-76.91)
2019	29.13 (24.85-34.20)	31.46 (26.67-36.91)	35.75 (30.19-43.01)	29.05 (24.78-34.08)	63.28 (53.89-74.32)

Table S6. Age-standardised rate YLDs with percentage changes between 1990 and 2010, 2010 and 2019, and 1990 and 2019 for the leading causes of diseases, disabilities, and injuries in Bangladesh

Cause	Rank		YLDs per 100,000 population (95% UI)			% change (95% UI) in age-standardised YLDs		
	1990	2019	1990	2010	2019	1990–2010	2010–2019	1990–2019
Other musculoskeletal disorders	4	1	664.1 (454.4-917.7)	777.2 (532.8-1070.1)	826.5 (568.1-1138.2)	17.0% (10.8 to 23)	6.3% (-0.4 to 13.1)	24.5% (15.3 to 32.7)
Depressive disorders	1	2	850.6 (578.6-1183.2)	812.8 (562.6-1147.7)	822.2 (563-1140.2)	-4.4% (-12.6 to 4.3)	1.2% (-8.2 to 11.6)	-3.3% (-9.4 to 3.4)
Low back pain	2	3	821.2 (574.5-1096)	792.1 (560.9-1074.1)	761 (536.3-1022.9)	-3.5% (-7.5 to 0.6)	-3.9% (-8.3 to 0.7)	-7.3% (-11.6 to -2.6)
Headache disorders	6	4	563 (93.9-1243.5)	570.6 (93.2-1249.5)	574.3 (94.1-1279.4)	1.3% (-2.1 to 4.8)	0.7% (-2.7 to 4.1)	2.0% (-1.8 to 5.4)
Age-related hearing loss	7	5	537.8 (366.6-764.3)	520.4 (353.7-734.8)	507 (344.7-719.1)	-3.2% (-6.5 to 0)	-2.6% (-5.7 to 0.4)	-5.7% (-9.3 to -2.1)
Dietary iron deficiency	3	6	815.1 (538.2-1188)	576 (374.3-841.5)	488.7 (313.2-738.7)	-29.3% (-38.2 to -20.2)	-15.2% (-22.8 to -6.4)	-40.0% (-48.5 to -29.8)
Blindness and vision loss	5	7	564.7 (394.6-782.3)	480.9 (331.7-670)	447.7 (305.6-632.6)	-14.9% (-18.6 to -10.8)	-6.9% (-9.8 to -3.6)	-20.7% (-24.9 to -15.8)
Neonatal disorders	15	8	235.5 (172.1-319.3)	304.6 (233-385.7)	447.6 (344.7-568.7)	29.4% (2.1 to 64.9)	46.9% (22.7 to 78.4)	90.1% (47.3 to 146.9)
Diabetes	12	9	273.5 (182.5-379.1)	368.1 (247.4-507.2)	397.2 (263.6-551.1)	34.6% (24.9 to 45.2)	7.9% (0.1 to 16)	45.2% (37.9 to 52.5)
Gynaecological disorders	8	10	383.4 (264.5-542.2)	378.8 (260.4-537.7)	381.5 (261.3-538.1)	-1.2% (-6 to 3.2)	0.7% (-3.2 to 4.4)	-0.5% (-6 to 4.6)
COPD	9	11	331.4 (274.8-380.9)	290.5 (129.6-454.7)	325.7 (267.7-374)	-12.3% (-59.4 to 38.4)	12.1% (-28.5 to 140.7)	-1.7% (-6.2 to 2.9)
Anxiety disorders	11	12	291.3 (195.4-413.5)	298.6 (198.1-420.6)	302.2 (200.1-428)	2.5% (-4.6 to 10.1)	1.2% (-5.9 to 8.3)	3.7% (-3.5 to 11.2)
Road injuries	10	13	311.7 (228.9-407.9)	280.3 (203.9-368.6)	293.5 (212.7-388.6)	-10.1% (-12.5 to -7.6)	4.7% (2.4 to 6.7)	-5.9% (-8.9 to -2.8)

Oral disorders	16	14	227.2 (120.6-393.9)	225.9 (118-394.5)	226.2 (117.5-394.9)	-0.6% (-3.3 to 1.7)	0.1% (-2.4 to 2.6)	-0.4% (-3.5 to 2.6)
Diarrhoeal disorders	13	15	267.5 (186.1-370.2)	207.1 (143.5-285.7)	218.1 (150.7-301.9)	-22.6% (-28.9 to -15.6)	5.3% (-1.2 to 12.3)	-18.5% (-24.8 to -11)
Endocrine, metabolic, blood, and immune disorders	18	16	202.9 (136.6-281.1)	188.6 (127.2-262.6)	183.8 (123.8-256)	-7.1% (-11.7 to -2.5)	-2.5% (-6.9 to 1.5)	-9.4% (-14.5 to -3.7)
Schizophrenia	19	17	178.5 (125.4-235.5)	177.7 (122.9-233.4)	176.6 (122.3-234.5)	-0.4% (-8 to 7.8)	-0.6% (-8.6 to 8.1)	-1.1% (-8.8 to 7.4)
Osteoarthritis	22	18	153.2 (77.7-304.4)	162.3 (81.8-322.6)	166.8 (85.4-331.7)	6.0% (2.3 to 9.6)	2.8% (-0.6 to 6.3)	8.9% (5.5 to 12.4)
Stroke	23	19	149 (105.9-190.3)	159 (114.7-201.1)	152.5 (109.6-192.6)	6.7% (1.3 to 12.1)	-4.1% (-9.2 to 0.8)	2.3% (-2.9 to 8.2)
Neck pain	24	20	138.5 (90.7-201.7)	139.4 (91-200.4)	139.7 (91.2-201.4)	0.7% (-2.7 to 4.2)	0.2% (-3.1 to 3.5)	0.9% (-2.5 to 4.4)
Upper digestive diseases	21	21	163.2 (99.1-255.4)	132.4 (75.6-217.3)	135.3 (78.3-223.3)	-18.8% (-26.5 to -12.7)	2.2% (-2.4 to 6.5)	-17.1% (-24.1 to -11.5)
Falls	33	22	86.5 (62.2-117.1)	101.6 (73.3-138.4)	126.1 (89.5-173.7)	17.4% (13.8 to 21)	24.0% (20.5 to 27.4)	45.7% (40.6 to 50.9)
Haemoglobinopathies	20	23	170.6 (114.2-244.4)	133.7 (87.4-194.4)	124.4 (81.1-183.2)	-21.7% (-29.2 to -14.6)	-6.9% (-13.6 to 0.5)	-27.1% (-35.2 to -17.8)
Alcohol use disorders	28	24	107.9 (68.2-155.8)	111.8 (71.5-162.1)	112.5 (72.5-164.6)	3.6% (-6.6 to 13.8)	0.6% (-8.2 to 10.9)	4.3% (-5 to 13.8)
Intestinal nematodes	17	27	216 (125.4-357)	108.4 (61.3-179.5)	89 (48.5-150)	-49.8% (-59.2 to -37.7)	-17.9% (-30.5 to -4.6)	-58.8% (-67.5 to -48.5)
Tuberculosis	14	36	241.5 (160.5-333.1)	94.9 (63-130.5)	64.4 (43.8-89)	-60.7% (-65.3 to -56.1)	-32.1% (-39 to -24.1)	-73.3% (-76.5 to -69.8)

Table S7. Age-standardised rate YLLs with percentage changes between 1990 and 2010, 2010 and 2019, and 1990 and 2019 for the leading causes of diseases, disabilities, and injuries in Bangladesh

**Cause**	**Rank**		**YLLs per 100,000 population (95% UI)**			**% change (95% UI) in age-standardised YLLs**		
	**1990**	**2019**	**1990**	**2010**	**2019**	**1990–2010**	**2010–2019**	**1990–2019**
Neonatal disorders	1	1	6822.9 (5941.8-7766)	4611.3 (3754.9-5568.1)	2770 (2081.8-3560.8)	-32.4% (-47.4 to -15)	-39.9% (-50.2 to -28.3)	-59.4% (-70.3 to -46.8)
Stroke	5	2	3656.8 (3117.5-4148.1)	3524.5 (2701.2-3941)	2548.6 (1950.4-3132)	-3.6% (-27.3 to 15.1)	-27.7% (-40.5 to -12.6)	-30.3% (-48.1 to -11.2)
Ischaemic heart disease	7	3	2449.4 (2052.2-2793.4)	2592.6 (2201.6-2882)	2270 (1785.3-2815.4)	5.8% (-10 to 28.2)	-12.4% (-29.7 to 7.3)	-7.3% (-28.5 to 21.5)
Lower respiratory infections	2	4	5474.3 (4703.3-6290.7)	2260.6 (1933.9-2639.6)	1281.8 (992.5-1598.4)	-58.7% (-66.6 to -48.8)	-43.3% (-54.8 to -31.2)	-76.6% (-83.2 to -69.1)
COPD	8	5	2073 (1621-2814.4)	1149.1 (896.5-1904.6)	818.5 (598.8-1382.5)	-44.6% (-53.3 to -28.4)	-28.8% (-42 to -13.9)	-60.5% (-70.1 to -47.3)
Congenital defects	12	6	1415.6 (853.5-2022.6)	970.4 (665.7-1342)	707.3 (399.1-1178.1)	-31.4% (-57.7 to 31)	-27.1% (-50.5 to 2.1)	-50.0% (-75.6 to 13)
Diarrhoeal diseases	3	7	5367.1 (4113.5-6832.2)	1005.7 (574-1850.5)	628.8 (322.4-1278.5)	-81.3% (-89 to -67)	-37.5% (-51.4 to -19.5)	-88.3% (-93.9 to -76.7)
Tuberculosis	4	8	4318.1 (3519.1-5102.6)	1199.6 (999.6-1485)	623 (478.4-858.2)	-72.2% (-76.7 to -64.6)	-48.1% (-57.3 to -37)	-85.6% (-88.9 to -79.8)
Cirrhosis	11	9	1502.2 (1173.8-1822.4)	794.4 (680.8-931)	574.8 (443.7-738.9)	-47.1% (-56.2 to -31.6)	-27.6% (-41.1 to -11.5)	-61.7% (-71.6 to -46.9)
Diabetes	20	10	595.8 (497.2-685.8)	639.6 (563-713.9)	537.2 (430.9-651.5)	7.3% (-8.9 to 29.9)	-16.0% (-30.8 to 1.2)	-9.8% (-30.4 to 17.2)
Other malignant neoplasms	22	11	515 (249.5-698.6)	458.4 (238-608)	440 (221.2-626)	-11.0% (-29.9 to 14.1)	-4.0% (-21.9 to 17.3)	-14.6% (-41.1 to 22.7)
Typhoid & paratyphoid	16	12	929.4 (445.4-1651.7)	545.8 (279.8-904.8)	374 (186.5-627.2)	-41.3% (-52.6 to -20.9)	-31.5% (-44.5 to -19.4)	-59.8% (-71 to -43.1)
Drowning	10	13	1664.4 (1224.2-2151)	803.5 (632.1-1006.5)	309.2 (233.8-455.3)	-51.7% (-62.5 to -37.2)	-61.5% (-69.7 to -47.9)	-81.4% (-86.9 to -72.7)
Road injuries	24	14	427.7 (321.9-601.1)	341.8 (273.7-394.2)	265.8 (191-331.5)	-20.1% (-46 to 7.2)	-22.3% (-34.6 to -6.5)	-37.9% (-62.5 to -9.6)
Chronic kidney disease	25	15	390.5 (259-494.6)	345.4 (303.6-392.9)	263.2 (214.1-318.8)	-11.6% (-31.3 to 27.2)	-23.8% (-35.7 to -10.4)	-32.6% (-50.9 to 0.2)

Hypertensive heart disease	26	16	378.3 (203.7-522.5)	292.9 (187.4-432.2)	251.2 (151.5-396.3)	-22.6% (-43.2 to 17.8)	-14.2% (-32.4 to 7)	-33.6% (-57.7 to 15.9)
Maternal disorders	14	17	1090.3 (761.8-1337.5)	261.5 (214.5-312.7)	223.3 (168.7-288.4)	-76.0% (-81.5 to -66.1)	-14.6% (-34.9 to 11)	-79.5% (-85.6 to -70.7)
Self-harm	19	18	605.8 (429-741.3)	295.2 (249.5-358)	218.7 (169.2-277.8)	-51.3% (-61 to -32.7)	-25.9% (-40.4 to -9.1)	-63.9% (-73.8 to -46.8)
Breast cancer	32	19	223.1 (165.9-287.5)	212.1 (181.4-250.9)	218.4 (170.3-276.4)	-4.9% (-28.3 to 34)	2.9% (-19.9 to 30.7)	-2.1% (-29.8 to 40.8)
Asthma	17	20	799.9 (563.2-1152)	306.1 (224.8-464.5)	190.1 (133.9-306)	-61.7% (-70.2 to -45.2)	-37.9% (-53.1 to -18.9)	-76.2% (-84.1 to -60.9)
Alzheimer's disease	37	21	183.3 (41.9-527)	190.4 (44.5-526.1)	189.7 (46.2-518.2)	3.9% (-8 to 20.4)	-0.3% (-14.7 to 15.1)	3.5% (-14.4 to 26.9)
Acute hepatitis	21	27	577.6 (449.5-693.7)	135.7 (116.1-159.9)	109.4 (83.4-142.8)	-31.0% (-44 to -7.9)	-4.1% (-21.6 to 19)	-33.8% (-51.1 to -8.8)
Pertussis	18	28	773.6 (68.8-2483.2)	144.1 (11.8-452.7)	106 (9.8-326)	-81.4% (-98.5 to 120.2)	-26.5% (-94.1 to 718.1)	-86.3% (-99 to 84.6)
Protein-energy malnutrition	6	34	2812.9 (2040.1-3711.4)	290.4 (218.3-388.9)	93.8 (55.6-144.5)	-89.7% (-93.3 to -84.1)	-67.7% (-79.3 to -49.4)	-96.7% (-98.3 to -93.8)
Tetanus	13	65	1249.5 (902.9-1683.4)	19.7 (12.1-31.3)	28.1 (10.3-55)	-98.4% (-99.1 to -97.3)	42.5% (-34.4 to 192.8)	-97.8% (-99.1 to -95.3)
Measles	15	74	991.2 (314.7-2242.4)	111.4 (36.9-260.1)	21.3 (6.2-50.9)	-88.8% (-93 to -82.7)	-80.9% (-90.1 to -65.4)	-97.8% (-98.9 to -96.3)
Malaria	9	111	1952.6 (279.6-7965.8)	188 (97.8-368.3)	3.6 (0.1-21.6)	-90.4% (-97.2 to -32.7)	-98.1% (-100 to -86.2)	-99.8% (-100 to -97.4)

Table S8. Age-standardised rates of deaths for NCDs, cardiovascular diseases and stroke in south Asia in 2019

Countries	Diseases	Deaths	Upper	Lower
Bangladesh	Cardiovascular diseases	283.87	341.78	220.65
Bhutan	Cardiovascular diseases	246.56	286.88	198.92
Nepal	Cardiovascular diseases	245.38	286.14	199.70
India	Cardiovascular diseases	256.37	292.57	220.98
Pakistan	Cardiovascular diseases	357.88	423.57	307.76
Bangladesh	Non-communicable diseases	543.53	641.78	453.08
Bhutan	Non-communicable diseases	620.30	703.99	525.16
Pakistan	Non-communicable diseases	814.48	942.91	712.88
India	Non-communicable diseases	604.42	680.50	530.69
Nepal	Non-communicable diseases	721.21	799.66	611.10
Bhutan	Stroke	69.42	85.44	54.26
Bangladesh	Stroke	140.04	169.50	107.04
Nepal	Stroke	80.44	98.61	63.44
India	Stroke	69.52	80.07	59.32
Pakistan	Stroke	114.86	139.21	98.69

Rate per 100,000 individuals

Table S9. Life expectancy at birth (years) during 1990 and 2019: comparison between Bangladesh and South Asian countries

Years	Bangladesh	Bhutan	India	Nepal	Pakistan
1990	58.19 (57.10-59.23)	60.33 (57.72-62.92)	59.58 (58.71-60.47)	58.32 (56.29-60.26)	61.05 (60.05-62.07)
1991	56.79 (55.74-57.80)	60.89 (58.30-63.43)	59.77 (58.84-60.64)	58.86 (56.83-60.69)	61.01 (60.02-61.99)
1992	60.24 (59.16-61.34)	61.54 (58.87-64.02)	60.00 (59.10-60.85)	59.76 (57.69-61.57)	60.88 (59.89-61.91)
1993	61.03 (59.93-62.12)	62.19 (59.56-64.54)	60.59 (59.69-61.44)	60.31 (58.39-62.03)	60.74 (59.68-61.79)
1994	61.79 (60.64-62.90)	62.65 (60.18-64.96)	61.21 (60.38-62.06)	61.19 (59.29-62.91)	60.56 (59.48-61.60)
1995	62.24 (61.05-63.45)	63.44 (61.01-65.63)	61.74 (60.89-62.61)	61.83 (60.04-63.56)	60.34 (59.23-61.44)
1996	63.08 (61.88-64.28)	64.23 (61.99-66.35)	62.05 (61.11-62.92)	62.57 (60.78-64.26)	60.44 (59.37-61.54)
1997	63.84 (62.67-65.00)	64.77 (62.62-66.85)	61.89 (61.02-62.70)	63.37 (61.56-65.06)	60.47 (59.45-61.63)
1998	64.52 (63.31-65.66)	65.38 (63.24-67.32)	62.11 (61.29-62.93)	64.02 (62.33-65.59)	60.69 (59.63-61.76)
1999	65.11 (63.99-66.14)	66.04 (64.12-67.99)	62.56 (61.76-63.31)	64.75 (63.12-66.33)	60.85 (59.81-61.92)
2000	65.47 (64.42-66.44)	65.74 (64.01-67.60)	62.94 (62.10-63.74)	65.39 (63.87-66.92)	61.04 (59.98-62.06)
2001	65.99 (64.99-66.93)	67.10 (65.44-68.98)	63.27 (62.42-64.08)	66.06 (64.60-67.60)	61.21 (60.09-62.28)
2002	66.34 (65.44-67.27)	67.68 (66.10-69.56)	63.79 (62.99-64.64)	66.16 (64.79-67.65)	61.35 (60.33-62.49)
2003	66.66 (65.82-67.50)	68.16 (66.59-70.16)	64.47 (63.63-65.29)	67.00 (65.65-68.52)	61.52 (60.36-62.59)
2004	67.12 (66.29-67.90)	68.64 (66.96-70.61)	65.20 (64.34-65.98)	67.24 (65.88-68.72)	61.77 (60.60-62.95)
2005	67.44 (66.61-68.31)	69.05 (67.37-71.02)	65.39 (64.54-66.26)	67.69 (66.34-69.12)	61.05 (59.80-62.28)
2006	67.79 (66.85-68.70)	69.47 (67.75-71.45)	65.59 (64.73-66.38)	68.01 (66.73-69.47)	62.32 (61.03-63.58)

2007	68.22 (67.28-69.16)	69.90 (68.02-71.92)	65.84 (64.96-66.66)	68.26 (66.94-69.68)	62.69 (61.34-64.02)
2008	68.98 (67.97-69.97)	70.28 (68.39-72.38)	66.08 (65.22-66.89)	68.52 (67.16-69.94)	62.92 (61.46-64.52)
2009	69.58 (68.52-70.58)	70.55 (68.67-72.73)	66.81 (65.97-67.66)	68.76 (67.23-70.18)	63.15 (61.58-64.84)
2010	70.04 (69.01-71.12)	71.00 (69.19-73.11)	67.28 (66.42-68.14)	69.04 (67.46-70.48)	63.27 (61.66-65.02)
2011	71.14 (69.89-72.32)	71.32 (69.48-73.61)	67.56 (66.73-68.47)	69.22 (67.62-70.71)	63.62 (61.91-65.44)
2012	72.26 (70.80-73.69)	71.61 (69.67-73.99)	68.01 (67.21-68.84)	69.47 (67.78-71.07)	63.82 (62.05-65.70)
2013	72.81 (71.29-74.40)	71.85 (69.91-74.26)	68.36 (67.55-69.13)	69.68 (67.99-71.39)	64.00 (62.24-65.94)
2014	73.05 (71.38-74.75)	72.08 (70.04-74.52)	69.00 (68.21-69.77)	69.91 (68.16-71.75)	64.17 (62.24-66.03)
2015	73.59 (71.82-75.34)	72.29 (70.10-74.76)	69.36 (68.57-70.12)	69.35 (67.64-71.17)	64.42 (62.50-66.41)
2016	73.91 (72.06-75.79)	72.52 (70.37-74.98)	69.96 (69.08-70.81)	70.37 (68.62-72.31)	64.66 (62.69-66.61)
2017	73.90 (72.00-75.80)	72.73 (70.64-75.10)	70.34 (69.24-71.39)	70.60 (68.89-72.61)	64.93 (62.87-67.01)
2018	74.24 (72.25-76.26)	72.95 (70.83-75.34)	70.46 (69.12-71.76)	70.84 (69.16-72.91)	65.45 (63.52-67.45)
2019	74.55 (72.41-76.68)	73.17 (70.91-75.56)	70.77 (69.29-72.21)	71.07 (69.35-73.17)	65.86 (63.79-67.79)

Table S10. Healthy life expectancy (years) at birth during 1990 and 2019: comparison between Bangladesh and South Asian countries

**Years**	**Bangladesh**	**Bhutan**	**India**	**Nepal**	**Pakistan**
1990	50.70 (48.33-52.76)	52.31 (49.14-55.34)	51.05 (48.46-53.29)	50.43 (47.72-53.06)	53.18 (50.83-55.32)
1991	49.47 (47.28-51.42)	52.79 (49.56-55.79)	51.21 (48.54-53.45)	50.91 (48.29-53.47)	53.16 (50.78-55.37)
1992	52.40 (50.04-54.47)	53.36 (50.17-56.31)	51.39 (48.75-53.69)	51.68 (48.92-54.22)	53.08 (50.63-55.25)
1993	53.10 (50.67-55.33)	53.92 (50.85-56.81)	51.86 (49.15-54.19)	52.16 (49.51-54.72)	52.98 (50.56-55.27)
1994	53.76 (51.31-56.09)	54.32 (51.30-57.24)	52.35 (49.70-54.79)	52.90 (50.20-55.40)	52.85 (50.33-55.03)
1995	54.17 (51.67-56.54)	55.00 (52.03-58.00)	52.77 (50.03-55.08)	53.45 (50.64-56.18)	52.69 (50.32-54.91)
1996	54.89 (52.38-57.23)	55.66 (52.75-58.73)	53.05 (50.40-55.37)	54.05 (51.21-56.85)	52.78 (50.39-54.99)
1997	55.53 (52.95-57.88)	56.14 (53.26-59.07)	53.00 (50.21-55.26)	54.72 (51.85-57.49)	52.81 (50.33-55.15)
1998	56.13 (53.49-58.49)	56.67 (53.77-59.53)	53.22 (50.66-55.51)	55.24 (52.39-58.01)	52.99 (50.61-55.20)
1999	56.65 (54.03-58.98)	57.24 (54.38-60.11)	53.59 (50.91-55.97)	55.84 (53.04-58.55)	53.12 (50.76-55.39)
2000	56.98 (54.35-59.32)	57.01 (54.22-59.73)	53.91 (51.34-56.32)	56.38 (53.54-59.04)	53.28 (50.97-55.52)
2001	57.42 (54.65-59.72)	58.14 (55.30-60.84)	54.20 (51.55-56.61)	56.95 (54.06-59.57)	53.42 (51.11-55.64)
2002	57.74 (55.00-60.01)	58.64 (55.77-61.27)	54.64 (52.02-57.09)	57.04 (54.12-59.58)	53.53 (51.10-55.80)
2003	58.03 (55.49-60.29)	59.05 (56.13-61.70)	55.17 (52.60-57.58)	57.77 (54.98-60.35)	53.67 (51.32-55.96)
2004	58.41 (55.95-60.65)	59.45 (56.60-62.20)	55.73 (53.07-58.26)	58.00 (55.27-60.56)	53.87 (51.46-56.06)
2005	58.71 (56.16-60.98)	59.81 (56.88-62.58)	55.92 (53.27-58.47)	58.40 (55.68-60.92)	53.26 (50.83-55.53)
2006	59.04 (56.39-61.33)	60.18 (57.21-62.97)	56.13 (53.38-58.53)	58.71 (55.94-61.22)	54.31 (51.84-56.49)

2007	59.42 (56.76-61.75)	60.56 (57.57-63.39)	56.38 (53.67-58.88)	58.98 (56.19-61.48)	54.64 (52.04-56.95)
2008	60.07 (57.38-62.49)	60.90 (57.94-63.72)	56.62 (53.86-59.05)	59.25 (56.44-61.79)	54.84 (52.34-57.20)
2009	60.59 (57.92-63.05)	61.14 (58.15-64.09)	57.21 (54.45-59.65)	59.50 (56.64-62.02)	55.04 (52.44-57.54)
2010	61.00 (58.23-63.45)	61.52 (58.55-64.45)	57.58 (54.86-60.07)	59.76 (56.88-62.32)	55.14 (52.66-57.61)
2011	61.83 (58.97-64.53)	61.80 (58.73-64.66)	57.82 (54.99-60.26)	59.94 (57.00-62.46)	55.43 (52.96-58.06)
2012	62.66 (59.72-65.50)	62.03 (58.96-64.91)	58.20 (55.39-60.81)	60.16 (57.25-62.82)	55.59 (52.79-58.22)
2013	63.10 (60.07-66.03)	62.22 (59.16-65.16)	58.52 (55.64-61.18)	60.36 (57.47-63.06)	55.73 (53.17-58.44)
2014	63.32 (60.23-66.28)	62.40 (59.30-65.42)	59.06 (56.15-61.67)	60.57 (57.61-63.35)	55.86 (53.04-58.49)
2015	63.74 (60.67-66.79)	62.57 (59.41-65.52)	59.36 (56.44-62.01)	60.09 (57.20-62.91)	56.07 (53.30-58.81)
2016	64.03 (60.95-66.98)	62.74 (59.56-65.74)	59.85 (56.96-62.56)	60.93 (57.95-63.70)	56.24 (53.28-58.85)
2017	64.09 (60.95-67.07)	62.90 (59.63-65.87)	60.16 (57.23-62.84)	61.10 (58.19-63.95)	56.45 (53.48-59.19)
2018	64.34 (61.10-67.26)	63.07 (59.78-66.04)	60.27 (57.28-63.11)	61.30 (58.47-64.12)	56.87 (54.12-59.59)
2019	64.53 (61.33-67.44)	63.23 (59.92-66.30)	60.52 (57.38-63.34)	61.51 (58.57-64.41)	57.20 (54.31-60.06)

Table S11. Total fertility rate during 1990 and 2019: comparison between Bangladesh and South Asian countries

Years	Bangladesh	Bhutan	India	Nepal	Pakistan
1990	4.38 (4.27-4.50)	4.95 (4.47-5.43)	4.01 (3.78-4.23)	5.54 (5.35-5.71)	6.61 (6.40-6.80)
1991	4.18 (4.06-4.30)	4.85 (4.38-5.33)	3.94 (3.73-4.16)	5.44 (5.25-5.62)	6.55 (6.33-6.75)
1992	3.98 (3.86-4.10)	4.76 (4.28-5.24)	3.87 (3.66-4.09)	5.33 (5.15-5.51)	6.49 (6.26-6.69)
1993	3.80 (3.67-3.91)	4.65 (4.19-5.12)	3.80 (3.59-4.01)	5.21 (5.03-5.39)	6.42 (6.18-6.65)
1994	3.65 (3.51-3.77)	4.55 (4.08-5.00)	3.72 (3.54-3.93)	5.08 (4.90-5.26)	6.35 (6.09-6.60)
1995	3.53 (3.40-3.66)	4.44 (3.96-4.88)	3.65 (3.48-3.84)	4.93 (4.75-5.12)	6.28 (6.00-6.54)
1996	3.43 (3.30-3.55)	4.32 (3.86-4.77)	3.57 (3.41-3.75)	4.78 (4.60-4.96)	6.21 (5.93-6.47)
1997	3.33 (3.20-3.45)	4.20 (3.76-4.66)	3.49 (3.34-3.65)	4.61 (4.44-4.80)	6.13 (5.86-6.40)
1998	3.22 (3.09-3.36)	4.08 (3.63-4.53)	3.40 (3.25-3.54)	4.44 (4.26-4.62)	6.05 (5.79-6.31)
1999	3.12 (2.98-3.26)	3.95 (3.52-4.40)	3.31 (3.16-3.45)	4.26 (4.09-4.43)	5.97 (5.72-6.21)
2000	3.02 (2.87-3.17)	3.82 (3.41-4.26)	3.22 (3.07-3.36)	4.07 (3.91-4.24)	5.88 (5.62-6.13)
2001	2.93 (2.79-3.08)	3.69 (3.28-4.11)	3.13 (2.99-3.28)	3.88 (3.73-4.05)	5.79 (5.52-6.05)
2002	2.86 (2.71-3.01)	3.55 (3.15-3.96)	3.06 (2.92-3.22)	3.70 (3.54-3.85)	5.70 (5.42-5.96)
2003	2.79 (2.64-2.93)	3.42 (3.02-3.84)	3.00 (2.86-3.15)	3.51 (3.36-3.67)	5.60 (5.32-5.87)
2004	2.71 (2.57-2.85)	3.28 (2.89-3.70)	2.94 (2.80-3.08)	3.34 (3.19-3.50)	5.50 (5.23-5.78)
2005	2.63 (2.50-2.76)	3.17 (2.76-3.58)	2.87 (2.74-3.02)	3.18 (3.02-3.33)	5.40 (5.13-5.67)
2006	2.54 (2.41-2.67)	3.07 (2.66-3.48)	2.81 (2.68-2.95)	3.03 (2.88-3.20)	5.29 (5.01-5.56)
2007	2.46 (2.33-2.59)	2.97 (2.56-3.38)	2.74 (2.62-2.86)	2.92 (2.76-3.09)	5.17 (4.89-5.45)
2008	2.38 (2.26-2.51)	2.87 (2.46-3.28)	2.67 (2.55-2.79)	2.82 (2.66-2.99)	5.05 (4.78-5.33)
2009	2.31 (2.19-2.44)	2.77 (2.36-3.17)	2.60 (2.49-2.72)	2.72 (2.55-2.89)	4.93 (4.65-5.20)
2010	2.25 (2.13-2.37)	2.67 (2.27-3.06)	2.54 (2.42-2.65)	2.62 (2.45-2.79)	4.80 (4.52-5.08)
2011	2.19 (2.07-2.31)	2.58 (2.19-2.94)	2.47 (2.36-2.58)	2.52 (2.35-2.70)	4.67 (4.38-4.95)
2012	2.14 (2.02-2.26)	2.51 (2.12-2.87)	2.41 (2.30-2.52)	2.44 (2.26-2.63)	4.54 (4.23-4.83)
2013	2.09 (1.97-2.22)	2.44 (2.07-2.79)	2.36 (2.24-2.48)	2.37 (2.19-2.57)	4.40 (4.08-4.72)
2014	2.05 (1.91-2.18)	2.36 (2.03-2.71)	2.30 (2.16-2.44)	2.31 (2.12-2.52)	4.27 (3.92-4.63)

2015	2.00 (1.85-2.14)	2.29 (1.96-2.65)	2.24 (2.08-2.40)	2.25 (2.05-2.47)	4.13 (3.76-4.53)
2016	1.95 (1.79-2.11)	2.22 (1.89-2.57)	2.17 (2.00-2.35)	2.19 (1.97-2.43)	4.00 (3.60-4.43)
2017	1.89 (1.71-2.07)	2.15 (1.83-2.49)	2.10 (1.92-2.29)	2.13 (1.90-2.38)	3.86 (3.46-4.30)
2018	1.84 (1.66-2.03)	2.09 (1.78-2.43)	2.05 (1.85-2.24)	2.07 (1.84-2.35)	3.76 (3.35-4.22)
2019	1.82 (1.63-2.02)	2.06 (1.76-2.42)	2.02 (1.82-2.22)	2.05 (1.83-2.33)	3.72 (3.31-4.20)

Section 5. Data sources

Table S12. Data sources used for estimation of burden of diseases in Bangladesh.

Time period covered: **1990–2019**. Total documents: **688**	
**Data type (number of documents)**	**Data source**
Administrative data (33)	All India Institute of Medical Sciences, New Delhi (AIIMS), Bangabandhu Sheikh Mujib Medical University, De Soysa Maternity Hospital, Gadjah Mada University, Institute of Medicine, Tribhuvan University, Queen Sirikt National Institute of Child Health (Thailand), World Health Organization Regional Office for South-East Asia (SEARO). WHO South East Asia Regional Neonatal-Perinatal Database Report 2007-2008. New Delhi, India: World Health Organization Regional Office for South-East Asia (SEARO), 2008.
	Helen Keller International. Bangladesh Nutritional Surveillance Project National Rural Data Using the WHO Child Growth Standards 2006.
	Helen Keller International. Bangladesh Nutritional Surveillance Project Data on Urban Poor Using the WHO Child Growth Standards 2006.
	Helen Keller International. Bangladesh Nutritional Surveillance Project Data on Urban Poor Using the WHO Child Growth Standards 2005.
	Helen Keller International. Bangladesh Nutritional Surveillance Project Rural Data Using the WHO Child Growth Standards 2005.
	Helen Keller International. Bangladesh Nutritional Surveillance Project Data on Urban Poor Using the WHO Child Growth Standards 2004.
	Helen Keller International. Bangladesh Nutritional Surveillance Project National Rural Data Using the WHO Child Growth Standards 2004.
	Helen Keller International. Bangladesh Nutritional Surveillance Project National Rural Data Using the WHO Child Growth Standards 2003.
	Helen Keller International. Bangladesh Nutritional Surveillance Project Data on Urban Poor Using the WHO Child Growth Standards 2003.
	Helen Keller International. Bangladesh Nutritional Surveillance Project National Rural Data Using the WHO Child Growth Standards 2002.

	Helen Keller International. Bangladesh Nutritional Surveillance Project Data on Urban Poor Using the WHO Child Growth Standards 2002.
	Helen Keller International. Bangladesh Nutritional Surveillance Project National Rural Data Using the WHO Child Growth Standards 2001.
	Helen Keller International. Bangladesh Nutritional Surveillance Project Data on Urban Poor Using the WHO Child Growth Standards 2001.
	Helen Keller International. Bangladesh Nutritional Surveillance Project National Rural Data Using the WHO Child Growth Standards 2000.
	Helen Keller International. Bangladesh Nutritional Surveillance Project Data on Urban Poor Using the WHO Child Growth Standards 2000.
	Helen Keller International. Bangladesh Nutritional Surveillance Project National Rural Data Using the WHO Child Growth Standards 1999.
	Helen Keller International. Bangladesh Nutritional Surveillance Project Data on Urban Poor Using the WHO Child Growth Standards 1999.
	Helen Keller International. Bangladesh Nutritional Surveillance Project Data on Rural National Using the WHO Child Growth Standards 1998.
	Helen Keller International. Bangladesh Nutritional Surveillance Project Data on Urban Poor Using the WHO Child Growth Standards 1998.
	Helen Keller International. Bangladesh Nutritional Surveillance Project Data on Urban Poor Using the WHO Child Growth Standards 1997.
	Helen Keller International. Bangladesh Nutritional Surveillance Project Data on Urban Poor Using the WHO Child Growth Standards 1996.
	Helen Keller International. Bangladesh Nutritional Surveillance Project National Rural Data Using the WHO Child Growth Standards 1996.
	Kidney Foundation (Bangladesh). Situation of Kidney Disease in Bangladesh. Kidney Foundation [Internet]. Dhaka, Bangladesh: Kidney Foundation (Bangladesh); [updated 2011].
	Helen Keller International. Bangladesh Nutritional Surveillance Project Data on Urban Poor Using the WHO Child Growth Standards 1995.

	Helen Keller International. Bangladesh Nutritional Surveillance Project National Rural Data Using the WHO Child Growth Standards 1995.
	Helen Keller International. Bangladesh Nutritional Surveillance Project Data on Urban Poor Using the WHO Child Growth Standards 1994.
	Helen Keller International. Bangladesh Nutritional Surveillance Project National Rural Data Using the WHO Child Growth Standards 1994.
	Helen Keller International. Bangladesh Nutritional Surveillance Project Data on Urban Poor Using the WHO Child Growth Standards 1993.
	Helen Keller International. Bangladesh Nutritional Surveillance Project National Rural Data Using the WHO Child Growth Standards 1993.
	Helen Keller International. Bangladesh Nutritional Surveillance Project Data on Urban Poor Using the WHO Child Growth Standards 1992.
	Helen Keller International. Bangladesh Nutritional Surveillance Project National Rural Data Using the WHO Child Growth Standards 1992.
	Helen Keller International. Bangladesh Nutritional Surveillance Project Data on Urban Poor Using the WHO Child Growth Standards 1991.
	Helen Keller International. Bangladesh Nutritional Surveillance Project National Rural Data Using the WHO Child Growth Standards 1991.
Census (6)	Bangladesh Bureau of Statistics (BBS). Bangladesh Population and Housing Census 2011.
	Minnesota Population Center, Bangladesh Bureau of Statistics. Bangladesh Population and Housing Census 2011 from the Integrated Public Use Microdata Series, International: [Machine-readable database]. Minneapolis: University of Minnesota, 2013.
	Bangladesh Bureau of Statistics (BBS). Bangladesh Population and Housing Census 2001.
	Minnesota Population Center, Bangladesh Bureau of Statistics. Bangladesh Population and Housing Census 2001 from the Integrated Public Use Microdata Series, International: [Machine-readable database]. Minneapolis: University of Minnesota, 2013.
	Bangladesh Bureau of Statistics (BBS). Bangladesh Population and Housing Census 1991.

	Minnesota Population Center, Bangladesh Bureau of Statistics. Bangladesh Population and Housing Census 1991 from the Integrated Public Use Microdata Series, International: [Machine-readable database]. Minneapolis: University of Minnesota, 2013.
Demographic surveillance (5)	Hanifi SMA, Sulatan A, Mia MN, Hoque S, Mahmood SS, Iqbal M, Bhuiya A .International Centre for Diarrhoeal Disease Research, Bangladesh (ICDDR,B). Chakaria health and demographic surveillance system report 2016: focusing on the Sustainable Development Goals. 2018.
	Centers for Disease Control and Prevention (CDC), Child Health and Mortality Prevention Surveillance (CHAMPS) Network, Deloitte Consulting LLP, Emory Global Health Institute (EGHI), International Association of National Public Health Institutes (IANPHI), Task Force for Global Health, Emory University. Child Health and Mortality Prevention Surveillance (CHAMPS) Network Program.
	Centers for Disease Control and Prevention (CDC), INDEPTH, International Vaccine Institute. Bangladesh - Abhoynagar, Mirsarai, and Kamalapur Health and Demographic Surveillance System.
	INDEPTH. Africa, Asia, Oceania - INDEPTH Network Cause-Specific Mortality - Release 2014. Accra, Ghana: INDEPTH, 2014.
	INDEPTH. INDEPTHStats Desktop Version. Accra, Ghana: INDEPTH.
Disease registry (3)	United States Renal Data System. 2015 USRDS Annual Data Report: Epidemiology of Kidney Disease in the United States. Bethesda, MD, United States: National Institute of Diabetes and Digestive and Kidney Diseases, National Institutes of Health, 2015.
	International Society of Nephrology (ISN). International Society of Nephrology Kidney Disease Data Center 2006-2009.
	Rashid HU, Kidney Foundation (Bangladesh). Bangladesh Renal Registry 2002-2013. [Unpublished].
Environmental monitoring (10)	U.S. Department of State, United States Environmental Protection Agency. Bangladesh - Dhaka AirNow Air Quality Data 2017.
	U.S. Department of State, United States Environmental Protection Agency. Bangladesh - Dhaka AirNow Air Quality Data 2016.
	Ministry of Environment and Forests (Bangladesh). Bangladesh Clean Air and Sustainable Environment Project PM2.5 and PM10 Data 2015.
	Ministry of Environment and Forests (Bangladesh). Bangladesh Clean Air and Sustainable Environment Project PM2.5 and PM10 Data 2014.
	Bandung Institute of Technology (Indonesia), Council for Scientific and Industrial Research (CSIR) (South Africa), Dalhousie University, Emory University (United States), Indian Institute of Technology Kanpur, Institute of Scientific and Technological Research for Defense (CITEDEF) (Argentina), Manila Observatory (Philippines), National Park Service, U.S. Department of

	the Interior, SPARTAN Global Particulate Matter Network, Tsinghua University, University of Dhaka, University of Ilorin, Vietnam Academy of Science and Technology. SPARTAN Global Particulate Matter Network Chemical Speciation Data. Halifax, Canada: SPARTAN Global Particulate Matter Network.
	Ministry of Environment and Forests (Bangladesh). Bangladesh Air Quality Status and Trends 2013-2015. Dhaka, Bangladesh: Ministry of Environment and Forests (Bangladesh), 2016.
	Ministry of Environment and Forests (Bangladesh). Bangladesh Summary Report on Monthly Air Quality Data December 2012. Dhaka, Bangladesh: Ministry of Environment and Forests (Bangladesh).
	Ministry of Environment and Forests (Bangladesh). Bangladesh Summary Report on Monthly Air Quality Data November 2012. Dhaka, Bangladesh: Ministry of Environment and Forests (Bangladesh).
	Clean Air Asia. Asia Air Quality Annual PM10 Averages 2005-2012, as provided by Clean Air Asia. [Unpublished].
	Hopke, Philip K. (Bayard D. Clarkson Distinguished Professor, Director, Institute for a Sustainable Environment, and Director, Center for Air Resources Engineering and Science, Clarkson University, Potsdam). Email regarding South and Southeast Asia Air Quality Annual Averages for PM2.5 and PM10 2002-2012 to: Michael Brauer (Member GBD 2013 Core Analytic Group; Professor, Faculty of Medicine, School of Population and Public Health, The University of British Columbia, Vancouver, BC Canada). 2014 March 4.
Epi surveillance (45)	World Health Organization (WHO). WHO Global Health Observatory Interactive Graph - Number of Cases of Cutaneous Leishmaniasis Reported 2015. Geneva, Switzerland: World Health Organization (WHO).
	World Health Organization (WHO). WHO Global Health Observatory Interactive Graph - Number of Cases of Visceral Leishmaniasis Reported 2015. Geneva, Switzerland: World Health Organization (WHO).
	World Health Organization (WHO). Bangladesh WHO Leishmaniasis Country Profiles 2014. Geneva, Switzerland: World Health Organization (WHO), 2016.
	World Health Organization (WHO). WHO Global Health Observatory Interactive Graph - Number of Cases of Cutaneous Leishmaniasis Reported 2014. Geneva, Switzerland: World Health Organization (WHO).
	World Health Organization (WHO). WHO Global Health Observatory Interactive Graph - Number of Cases of Visceral Leishmaniasis Reported 2014. Geneva, Switzerland: World Health Organization (WHO).

	Institute of Epidemiology, Disease Control & Research (Bangladesh). IEDCR Dengue Report on 12/31/2018. Dhaka, Bangladesh: Institute of Epidemiology, Disease Control & Research (Bangladesh), 2018.
	World Health Organization (WHO). WHO Global Health Observatory Interactive Graph - Number of Cases of Cutaneous Leishmaniasis Reported 2013. Geneva, Switzerland: World Health Organization (WHO).
	World Health Organization (WHO). WHO Global Health Observatory Interactive Graph - Number of Cases of Visceral Leishmaniasis Reported 2013. Geneva, Switzerland: World Health Organization (WHO).
	World Health Organization (WHO). WHO Global Health Observatory Interactive Graph - Number of Cases of Cutaneous Leishmaniasis Reported 2012. Geneva, Switzerland: World Health Organization (WHO).
	World Health Organization (WHO). WHO Global Health Observatory Interactive Graph - Number of Cases of Visceral Leishmaniasis Reported 2012. Geneva, Switzerland: World Health Organization (WHO).
	World Health Organization (WHO). WHO Global Health Observatory Interactive Graph - Number of Cases of Cutaneous Leishmaniasis Reported 2011. Geneva, Switzerland: World Health Organization (WHO).
	World Health Organization (WHO). WHO Global Health Observatory Interactive Graph - Number of Cases of Visceral Leishmaniasis Reported 2011. Geneva, Switzerland: World Health Organization (WHO).
	World Health Organization (WHO). WHO Global Health Observatory Interactive Graph - Number of Cases of Cutaneous Leishmaniasis Reported 2010. Geneva, Switzerland: World Health Organization (WHO).
	World Health Organization (WHO). WHO Global Health Observatory Interactive Graph - Number of Cases of Visceral Leishmaniasis Reported 2010. Geneva, Switzerland: World Health Organization (WHO).
	World Health Organization (WHO). WHO Global Health Observatory - Antenatal Care Attendees Tested for Syphilis Data by Country. Geneva, Switzerland: World Health Organization (WHO).
	World Health Organization (WHO). WHO Global Health Observatory Interactive Graph - Number of Cases of Cutaneous Leishmaniasis Reported 2009. Geneva, Switzerland: World Health Organization (WHO).

	World Health Organization (WHO). WHO Global Health Observatory Interactive Graph - Number of Cases of Visceral Leishmaniasis Reported 2009. Geneva, Switzerland: World Health Organization (WHO).
	World Health Organization (WHO). WHO Global Health Observatory Interactive Graph - Number of Cases of Visceral Leishmaniasis Reported 2008. Geneva, Switzerland: World Health Organization (WHO).
	World Health Organization (WHO). WHO Global Health Observatory Interactive Graph - Number of Cases of Cutaneous Leishmaniasis Reported 2008. Geneva, Switzerland: World Health Organization (WHO).
	World Health Organization (WHO). WHO Global Health Observatory Interactive Graph - Number of Cases of Visceral Leishmaniasis Reported 2007. Geneva, Switzerland: World Health Organization (WHO).
	Institute of Epidemiology, Disease Control & Research (Bangladesh). IEDCR Dengue Report on 11/6/2017. Dhaka, Bangladesh: Institute of Epidemiology, Disease Control & Research (Bangladesh), 2017.
	World Health Organization (WHO). WHO Global Health Observatory Interactive Graph - Number of Cases of Cutaneous Leishmaniasis Reported 2007. Geneva, Switzerland: World Health Organization (WHO).
	World Health Organization (WHO). WHO Global Health Observatory Interactive Graph - Number of Cases of Cutaneous Leishmaniasis Reported 2006. Geneva, Switzerland: World Health Organization (WHO).
	World Health Organization (WHO). WHO Global Health Observatory Interactive Graph - Number of Cases of Visceral Leishmaniasis Reported 2006. Geneva, Switzerland: World Health Organization (WHO).
	Joint United Nations Program on HIV/AIDS (UNAIDS), United Nations Children's Fund (UNICEF), World Health Organization (WHO). Bangladesh Global AIDS Response Progress Reporting (GARPR) System - Antenatal Care Attendees Positive for Syphilis.
	World Health Organization (WHO). WHO Global Health Observatory Interactive Graph - Number of Cases of Cutaneous Leishmaniasis Reported 2005. Geneva, Switzerland: World Health Organization (WHO).
	World Health Organization (WHO). WHO Global Health Observatory Interactive Graph - Number of Cases of Visceral Leishmaniasis Reported 2005. Geneva, Switzerland: World Health Organization (WHO).

	World Health Organization (WHO). WHO Global Health Observatory Interactive Graph - Number of Cases of Cutaneous Leishmaniasis Reported 2004. Geneva, Switzerland: World Health Organization (WHO).
	World Health Organization (WHO). WHO Global Health Observatory Interactive Graph - Number of Cases of Visceral Leishmaniasis Reported 2004. Geneva, Switzerland: World Health Organization (WHO).
	World Health Organization Regional Office for South-East Asia (SEARO). WHO South-East Asia Region: Reported Cases and Deaths of Dengue from 2003 to 2012. New Delhi, India: World Health Organization Regional Office for South-East Asia (SEARO), 2013.
	World Health Organization (WHO). WHO Global Health Observatory Interactive Graph - Number of Cases of Cutaneous Leishmaniasis Reported 2003. Geneva, Switzerland: World Health Organization (WHO).
	World Health Organization (WHO). WHO Global Health Observatory Interactive Graph - Number of Cases of Visceral Leishmaniasis Reported 2003. Geneva, Switzerland: World Health Organization (WHO).
	World Health Organization (WHO). WHO Global Health Observatory Interactive Graph - Number of Cases of Cutaneous Leishmaniasis Reported 2002. Geneva, Switzerland: World Health Organization (WHO).
	World Health Organization (WHO). WHO Global Health Observatory Interactive Graph - Number of Cases of Visceral Leishmaniasis Reported 2002. Geneva, Switzerland: World Health Organization (WHO).
	World Health Organization (WHO). WHO Global Health Observatory Interactive Graph - Number of Cases of Cutaneous Leishmaniasis Reported 2001. Geneva, Switzerland: World Health Organization (WHO).
	World Health Organization (WHO). WHO Global Health Observatory Interactive Graph - Number of Cases of Visceral Leishmaniasis Reported 2001. Geneva, Switzerland: World Health Organization (WHO).
	World Health Organization Regional Office for the Eastern Mediterranean (EMRO-WHO). EMRO-WHO Weekly Epidemiological Monitor Issue 03, 20 January 2019. Cairo, Egypt: World Health Organization Regional Office for the Eastern Mediterranean (EMRO-WHO), 2017.
	World Health Organization Regional Office for South-East Asia (SEARO). Situation Update of Dengue in the SEA Region, 2010. New Delhi, India: World Health Organization Regional Office for South-East Asia (SEARO).

	World Health Organization (WHO). WHO Global Health Observatory Interactive Graph - Number of Cases of Cutaneous Leishmaniasis Reported 2000. Geneva, Switzerland: World Health Organization (WHO).
	World Health Organization (WHO). WHO Global Health Observatory Interactive Graph - Number of Cases of Visceral Leishmaniasis Reported 2000. Geneva, Switzerland: World Health Organization (WHO).
	World Health Organization (WHO). Bangladesh WHO Leishmaniasis Country Profile 1999-2010. Geneva, Switzerland: World Health Organization (WHO).
	World Health Organization (WHO). WHO Global Health Observatory Interactive Graph - Number of Cases of Cutaneous Leishmaniasis Reported 1999. Geneva, Switzerland: World Health Organization (WHO).
	World Health Organization (WHO). WHO Global Health Observatory Interactive Graph - Number of Cases of Visceral Leishmaniasis Reported 1999. Geneva, Switzerland: World Health Organization (WHO).
	World Health Organization (WHO). WHO Global Health Observatory Interactive Graph - Number of Cases of Cutaneous Leishmaniasis Reported 1998. Geneva, Switzerland: World Health Organization (WHO).
	World Health Organization (WHO). WHO Global Health Observatory Interactive Graph - Number of Cases of Visceral Leishmaniasis Reported 1998. Geneva, Switzerland: World Health Organization (WHO).
Estimate (14)	Middle East Respiratory Syndrome Coronavirus Environmental Suitability and Spillover Potential Geospatial Estimates
	Henipavirus Environmental Suitability and Spillover Potential Geospatial Estimates
	Institute for Health Metrics and Evaluation (IHME). Low- and Middle-Income Country Neonatal, Infant, and Under-5 Mortality Geospatial Estimates 2000-2017. Seattle, United States of America: Institute for Health Metrics and Evaluation (IHME), 2019.
	Institute for Health Metrics and Evaluation (IHME). Low- and Middle-Income Country Educational Attainment Geospatial Estimates 2000-2017. Seattle, United States of America: Institute for Health Metrics and Evaluation (IHME), 2019.
	Institute for Health Metrics and Evaluation (IHME). Low- and Middle-Income Country Child Growth Failure Geospatial Estimates 2000-2017. Seattle, United States of America: Institute for Health Metrics and Evaluation (IHME), 2020.

	Institute for Health Metrics and Evaluation (IHME). Low- and Middle-Income Country Childhood Overweight and Wasting Geospatial Estimates 2000-2017. Seattle, United States of America: Institute for Health Metrics and Evaluation (IHME), 2020.
	Institute for Health Metrics and Evaluation (IHME). Low- and Middle-Income Country Under-5 Diarrhea Incidence, Prevalence, and Mortality Geospatial Estimates 2000-2017. Seattle, United States of America: Institute for Health Metrics and Evaluation (IHME), 2020.
	Institute for Health Metrics and Evaluation (IHME). Low- and Middle-Income Country Oral Rehydration Therapy Coverage Geospatial Estimates 2000-2017. Seattle, United States of America: Institute for Health Metrics and Evaluation (IHME), 2020.
	Institute for Health Metrics and Evaluation (IHME). Global Lymphatic Filariasis Prevalence Geospatial Estimates 2000-2018. Seattle, United States of America: Institute for Health Metrics and Evaluation (IHME), 2020.
	Institute for Health Metrics and Evaluation (IHME). Low- and Middle-Income Country Drinking Water and Sanitation Facilities Access Geospatial Estimates 2000-2017. Seattle, United States of America: Institute for Health Metrics and Evaluation (IHME), 2020.
	Tuberculosis Prevalence Geospatial Estimates 2000-2015
	Low- and Middle-Income Country Household Air Pollution Geospatial Estimates 2000-2018
	Institute for Health Metrics and Evaluation (IHME). Low- and Middle-Income Country MCV1 Coverage Geospatial Estimates 2000-2019. Seattle, United States of America: Institute for Health Metrics and Evaluation (IHME), 2020.
	Centre for Health Informatics and Multiprofessional Education, University College London. Modell's Haemoglobinopathologist's Almanac. London, United Kingdom: Centre for Health Informatics and Multiprofessional Education, University College London, 2008.
Event data (7)	Reuters. Cyclone Bulbul death toll rises to 12 in Bangladesh, five fishermen missing. Reuters [Internet]. 2019 Nov 10.
	Xinhua News Agency. Bangladesh dengue fever death toll tops 100. Xinhua News Agency [Internet]. 2019 Oct 21; Asia & Pacific.
	Doctors Without Borders (MSF). Myanmar/Bangladesh: MSF surveys estimate that at least 6,700 Rohingya were killed during the attacks in Myanmar. Doctors Without Borders (MSF) [press release]. 2017 Dec 12.

	Domonoske C. Floods In South Asia Have Killed More Than 1,000 People This Summer. National Public Radio (NPR) [Internet]. 2017 Aug 29; International.
	Wikipedia. 2015 Mina Stampede. San Francisco, United States: Wikipedia; [updated 2016].
	Singh A. Flooding in Asia Continues to Displace Millions, Death Toll Rising Rapidly. CNN [Internet]. 2015 Aug 9. Asia.
	Climate Change and African Political Stability Project (CCAPS). Armed Conflict Location and Event Dataset, Realtime - Robert S. Strauss Center *as referenced in* Raleigh, Clionadh, Andrew Linke, Havard Hegre and Joakim Karlsen. 2010. Introducing ACLED-Armed Conflict Location and Event Data. *Journal of Peace Research* 47(5), 651-60.
Intervention study (1)	Handzel T, University of North Carolina at Chapel Hill. The Effect of Improved Drinking Water Quality on the Risk of Diarrhoeal Disease in an Urban Slum of Dhaka, Bangladesh: A Home Chlorination Intervention Trial. 1998.
Report (42)	Directorate General of Health Services, Ministry of Health and Family Welfare (Bangladesh). Bangladesh Health Bulletin 2017. Dhaka, Bangladesh: Directorate General of Health Services, Ministry of Health and Family Welfare (Bangladesh).
	Directorate General of Health Services, Ministry of Health and Family Welfare (Bangladesh). Bangladesh Health Bulletin 2015. Dhaka, Bangladesh: Directorate General of Health Services, Ministry of Health and Family Welfare (Bangladesh), 2015.
	Directorate General of Health Services, Ministry of Health and Family Welfare (Bangladesh). Bangladesh Health Bulletin 2016. Dhaka, Bangladesh: Directorate General of Health Services, Ministry of Health and Family Welfare (Bangladesh).
	Directorate General of Health Services, Ministry of Health and Family Welfare (Bangladesh). Bangladesh Communicable Disease Control Annual Report 2012. Dhaka, Bangladesh: Directorate General of Health Services, Ministry of Health and Family Welfare (Bangladesh), 2013.
	Bangladesh Bureau of Statistics (BBS). Bangladesh Statistical Yearbook 2010. Dhaka, Bangladesh: Bangladesh Bureau of Statistics (BBS), 2011.
	Luby S. SHEWA-B School Report 3. 2010. As it appears in Web Appendix 2 of *Disease burden from water: Hygiene and health: systematic review of handwashing practices worldwide and update of health effects*.
	Fulu E, Warner X, Miedema S, Jewkes R, Roselli T, Lang J, United Nations Entity for Gender Equality and the Empowerment of Women (UN Women), United Nations Development Programme (UNDP), United Nations Population Fund (UNFPA), United Nations Volunteers (UNV), Partners for Prevention. Why Do Some Men Use Violence Against Women and How Can We Prevent It? Quantitative Findings from the United Nations Mutli-country Study on Men and Violence in Asia and the Pacific. Bangkok, Thailand: Partners for Prevention. 2013.

	Directorate General of Health Services, Ministry of Health and Family Welfare (Bangladesh). Population Size Estimates for Most at Risk Populations for HIV in Bangladesh. Dhaka, Bangladesh: Directorate General of Health Services, Ministry of Health and Family Welfare (Bangladesh), 2009.
	BRAC, International Centre for Diarrhoeal Disease Research, Bangladesh (ICDDR,B). Bangladesh - Dhaka Causes of Maternal, Neonatal and Child Deaths: An Exploratory Study of Dhaka's Slum Dwellers. Dhaka, Bangladesh: International Centre for Diarrhoeal Disease Research, Bangladesh (ICDDR,B), 2009.
	Joint United Nations Program on HIV/AIDS (UNAIDS), Ministry of Health and Family Welfare (Bangladesh). Bangladesh UNGASS Country Progress Report 2010. Geneva, Switzerland: Joint United Nations Program on HIV/AIDS (UNAIDS), 2010.
	Directorate General of Health Services, Ministry of Health and Family Welfare (Bangladesh). Bangladesh National Malaria Strategic Plan 2015-2020. Dhaka, Bangladesh: Directorate General of Health Services, Ministry of Health and Family Welfare (Bangladesh), 2015.
	Luby S. SHEWA-B Midline Report 8. 2007. As it appears in Web Appendix 2 of *Disease burden from water: Hygiene and health: systematic review of handwashing practices worldwide and update of health effects*.
	Helen Keller International, Institute of Public Health Nutrition (Bangladesh). Bangladesh in Facts and Figures: 2004 Annual Report of the Nutritional Surveillance Project. New York City, United States: Helen Keller International, 2005.
	BRAC, Research Triangle Institute, Inc. (RTI), University of Dhaka, World Health Organization Regional Office for South-East Asia (SEARO). Impact of Tobacco-Related Illness in Bangladesh. New Delhi, India: World Health Organization Regional Office for South-East Asia (SEARO), 2007.
	Bangladesh Draft Report on Sentinel Tobacco Use Prevalence Study.
	Helen Keller International, Institute of Public Health Nutrition (Bangladesh). Bangladesh in Facts and Figures: Annual Report of the Nutritional Surveillance Project 2003. New York City, United States of America: Helen Keller International, 2004.
	World Health Organization (WHO). Global Programme to Eliminate Lymphatic Filariasis: Annual Report on Lymphtic Filariasis 2003. Geneva, Switzerland: World Health Organization (WHO), 2005.
	United Nations Population Fund (UNFPA). South Asia Conference for the Prevention and Treatment of Obstetric Fistula. New York City, United States: United Nations Population Fund (UNFPA), 2004.

	Bloem MW, Moench-Pfanner R, Panagides D, eds. Health and Nutritional Surveillance for Development. Singapore: Helen Keller Worldwide; 2003.
	Helen Keller International. Bangladesh Annual Report of the Nutritional Surveillance Project 2001. New York City, United States of America: Helen Keller International, 2002.
	Directorate General of Health Services, Ministry of Health and Family Welfare (Bangladesh). Bangladesh Health Bulletin 2008. Dhaka, Bangladesh: Directorate General of Health Services, Ministry of Health and Family Welfare (Bangladesh).
	Helen Keller International. Bangladesh Nutritional Surveillance Project Bulletin No. 16, April 2006. New York City, United States of America: Helen Keller International, 2006.
	Helen Keller International, Institute of Public Health Nutrition (Bangladesh). Bangladesh Nutrition and Health Surveillance in the Chittagong Hill Tracts.
	United Nations Children's Fund (UNICEF). Child Mortality and Injury in Asia. New York, United States: United Nations Children's Fund (UNICEF), 2008. (Innocenti Working Papers: Special Series on Child Injuries Nos. 1-4).
	TRL Limited (United Kingdom). The Involvement and Impact of Road Crashes on the Poor: Bangladesh and India Case Studies. Workingham, Berkshire, England: TRL Limited (United Kingdom), 2004.
	World Health Organization (WHO). Global Report on Antimalarial Efficacy and Drug Resistance 2000-2010. Geneva, Switzerland: World Health Organization (WHO), 2010.
	Helen Keller International. High Anemia Prevalence Among Bangladeshi Children in Urban Slums: An Ethical and Economic Rationale for Multi-micronutrient Supplementation. New York City, United States: Helen Keller International, 2000. (HKI-Nutrition Surveillance Project Bulletin, no. 1).
	Data International Ltd. (Bangladesh), German Technical Cooperation Agency (GTZ), Ministry of Health and Family Welfare (Bangladesh). Bangladesh National Health Accounts 1997-2007.
	World Health Organization (WHO). The Global Elimination of Congenital Syphilis: Rationale and Strategy for Action. Geneva, Switzerland: World Health Organization (WHO), 2007.
	Helen Keller International. Bangladesh Nutritional Surveillance Project Bulletin No. 10, March 2002. New York City, United States of America: Helen Keller International, 2002.
	Arimond M, Torheim LE, Wiesmann D, Joseph M, Carriquiry A, Academy for Educational Development (AED). Dietary Diversity as a Measure of the Micronutrient Adequacy of Women’s Diets: Results from Rural Bangladesh

	Site. Washington, DC: Food and Nutrition Technical Assistance II Project, Academy for Educational Development, 2009.
	Ministry of Health and Family Welfare (Bangladesh), Data International Ltd. (Bangladesh), Maxwell Stamp PLC (United Kingdom). Bangladesh National Health Accounts 1996-1997. Dhaka, Bangladesh: Ministry of Health and Family Welfare (Bangladesh), 1998.
	Roll Back Malaria Partnership, World Health Organization (WHO). Susceptibility of Plasmodium Falciparum to Antimalarial Drugs: Report on Global Monitoring 1996-2004. Geneva, Switzerland: World Health Organization (WHO), 2005.
	Institute for Health Policy (Sri Lanka), Data International Ltd. (Bangladesh), Ministry for Health and Family Welfare (Bangladesh), Ministry of Health (Sri Lanka). Bangladesh and Sri Lanka National Health Accounts, Child Health Subaccounts 1996-2002. Colombo, Sri Lanka: Institute for Health Policy (Sri Lanka), 2006.
	Family Health International. The Base of the Iceberg: Prevalence and Perceptions of Maternal Morbidity in Four Developing Countries. Durham, North Carolina: Family Health International, 1997.
	Osendarp S, Wahed MA, Baqui AH, Arifeen SE, Mahmud H, van Raaij J, Fuchs GJ. Determinants of Haemoglobin Level During Pregnancy and Relationship with Pregnancy Outcome in Bangladeshi Urban Poor. In: Khan MSI, Rahim MA, eds. Seventh Annual Scientific Conference. Dhaka, Bangladesh: International Center for Diarrhoeal Disease Research, Bangladesh, 1998.
	Nutritional Surveillance for Disaster Preparedness and Prevention of Nutritional Blindness: Seasonality of Nutritional Status. New York City, United States: Helen Keller International, 1993.
	United Nations Economic and Social Commission for Asia and the Pacific (ESCAP). United Nations Statistical Yearbook for Asia and the Pacific 2014. Bangkok, Thailand: United Nations Economic and Social Commission for Asia and the Pacific (ESCAP), 2014.
	Asian Development Bank (ADB). Key Indicators for Asia and the Pacific 2007. Manila, Philippines: Asian Development Bank (ADB), 2007.
	Community-Based Management of Acute Malnutrition (CMAM) Forum. Putting Child Kwarshiorkor on the Map. Community-Based Management of Acute Malnutrition (CMAM) Forum, 2016.
	Directorate General of Health Services, Ministry of Health and Family Welfare (Bangladesh). Bangladesh Health Bulletin 2013. Dhaka, Bangladesh: Directorate General of Health Services, Ministry of Health and Family Welfare (Bangladesh), 2014.
	Directorate General of Health Services, Ministry of Health and Family Welfare (Bangladesh). Bangladesh Health Bulletin 2014. Dhaka, Bangladesh: Directorate General of Health Services, Ministry of Health and Family Welfare (Bangladesh), 2014.

Scientific literature (380)	Cohen P, Friedrich P, Lam C, Jeha S, Metzger ML, Qaddoumi I, Naidu P, Faughnan L, Rodriguez-Galindo C, Bhakta N. Global Access to Essential Medicines for Childhood Cancer: A Cross-Sectional Survey. *J Glob Oncol*. 4: 1-11.
	Rahman A, Jagnoor J, Baset KU, Ryan D, Ahmed T, Rogers K, Hossain MJ, Ivers R, Rahman AKMF. Vulnerability to fatal drowning among the population in Southern Bangladesh: findings from a cross-sectional household survey. *BMJ Open*. 2019; 9(9): e027896.
	Bradshaw CJ, Bandi AS, Muktar Z, Hasan MA, Chowdhury TK, Banu T, Hailemariam M, Ngu F, Croaker D, Bankolé R, Sholadoye T, Olaomi O, Ameh E, Di Cesare A, Leva E, Ringo Y, Abdur-Rahman L, Salama R, Elhalaby E, Perera H, Parsons C, Cleeve S, Numanoglu A, Van As S, Sharma S, Lakhoo K. International Study of the Epidemiology of Paediatric Trauma: PAPSA Research Study. *World J Surg*. 2018; 42(6): 1885-1894.
	Ahmed F, Khan MR, Shaheen N, Ahmed KMU, Hasan A, Chowdhury IA, Chowdhury R. Anemia and iron deficiency in rural Bangladeshi pregnant women living in areas of high and low iron in groundwater. *Nutrition*. 2018; 51-52: 46-52.
	Amere GA, Nayak P, Salindri AD, Narayan KMV, Magee MJ. Contribution of Smoking to Tuberculosis Incidence and Mortality in High-Tuberculosis-Burden Countries. *Am J Epidemiol*. 2018; 187(9): 1846-1855.
	Rahman, Kazi Mizanur, Samarawickrema,Indira V. M., et al. . Performance of Kala-Azar Surveillance in Gaffargaon Subdistrict of Mymensingh, Bangladesh. *PLoS Negl Trop Dis*. 1969; 9.
	Islam A, Epstein JH, Rostal MK, Islam S, Rahman MZ, Hossain ME, Uzzaman MS, Munster VJ, Peiris M, Flora MS, Rahman M, Daszak P. Middle East Respiratory Syndrome Coronavirus Antibodies in Dromedary Camels, Bangladesh, 2015. *Emerg Infect Dis*. 2018; 24(5): 926-928.
	Hasnain MG, Maruf S, Nath P, Anuwarul A, Ahmed MNU, Chowdhury IH, Basher A. Managing Severe Tetanus without Ventilation Support in a Resource-limited Setting in Bangladesh. *Am J Trop Med Hyg*. 2018; 99(5): 1234-1238.
	Vidyasagaran AL, Siddiqi K, Kanaan M. Use of smokeless tobacco and risk of cardiovascular disease: A systematic review and meta-analysis. *Eur J Prev Cardiol*. 2016; 1970–1981.
	Mahmood A, Hu Y, Nasreen S, Hopke PK. Airborne particulate pollution measured in Bangladesh from 2014 to 2017. *Aerosol Air Qual Res*. 2019; 19(2): 272-281.

	Ene-Iordache B, Perico N, Bikbov B, Carminati S, Remuzzi A, Perna A, Islam N, Bravo RF, Aleckovic-Halilovic M, Zou H, Zhang L, Gouda Z, Tchokhonelidze I, Abraham G, Mahdavi-Mazdeh M, Gallieni M, Codreanu I, Togtokh A, Sharma SK, Koirala P, Uprety S, Ulasi I, Remuzzi G. Chronic kidney disease and cardiovascular risk in six regions of the world (ISN-KDDC): a cross-sectional study. *Lancet Glob Health*. 2016; 4(5): e307–e319.
	George CM, Monira S, Sack DA, Rashid MU, Saif-Ur-Rahman KM, Mahmud T, Rahman Z, Mustafiz M, Bhuyian SI, Winch PJ, Leontsini E, Perin J, Begum F, Zohura F, Biswas S, Parvin T, Zhang X, Jung D, Sack RB, Alam M. Randomized Controlled Trial of Hospital-Based Hygiene and Water Treatment Intervention (CHoBI7) to Reduce Cholera. *Emerg Infect Dis*. 2016; 220(2): 233-41.
	Islam FM, Chakrabarti R, Islam MT, Wahab M, Lamoureux E, Finger RP, Shaw JE. Prediabetes, diagnosed and undiagnosed diabetes, their risk factors and association with knowledge of diabetes in rural Bangladesh: The Bangladesh Population-based Diabetes and Eye Study. *J Diabetes*. 2015; 8(2): nan.
	Palafox B, McKee M, Balabanova D, AlHabib KF, Avezum AJ, Bahonar A, Ismail N, Chifamba J, Chow CK, Corsi DJ, Dagenais GR, Diaz R, Gupta R, Iqbal R, Kaur M, Khatib R, Kruger A, Kruger IM, Lanas F, Lopez-Jaramillo P, Minfan F, Mohan V, Mony PK, Oguz A, Palileo-Villanueva LM, Perel P, Poirier P, Rangarajan S, Rensheng L, Rosengren A, Soman B, Stuckler D, Subramanian SV, Teo K, Tsolekile LP, Wielgosz A, Yaguang P, Yeates K, Yongzhen M, Yusoff K, Yusuf R, Yusufali A, Zatońska K, Yusuf S. Wealth and cardiovascular health: a cross-sectional study of wealth-related inequalities in the awareness, treatment and control of hypertension in high-, middle- and low-income countries. *Int J Equity Health*. 2016; 15(1): 199.
	Tarafder KH, Akhtar N, Zaman MM, Rasel MA, Bhuiyan MR, Datta PG. Disabling hearing impairment in the Bangladeshi population. *J Laryngol Otol*. 2015; 129(2): 126–35.
	Muraduzzaman AKM, Khan MH, Parveen R, Sultana S, Alam AN, Akram A, Rahman M, Shirin T. Event based surveillance of Middle East Respiratory Syndrome Coronavirus (MERS- CoV) in Bangladesh among pilgrims and travelers from the Middle East: An update for the period 2013-2016. *PLoS One*. 2018; 13(1): e0189914.
	Saha SK, Ahmed ZB, Modak JK, Naziat H, Saha S, Uddin MA, Islam M, Baqui AH, Darmstadt GL, Schrag SJ. Group B Streptococcus among Pregnant Women and Newborns in Mirzapur, Bangladesh: Colonization, Vertical Transmission, and Serotype Distribution. *J Clin Microbiol*. 2017; 55(8): 2406-2412.
	Khan S, Nabi G, Naeem M, Ali L, Silburn PA, Mellick GD. A door-to-door survey to estimate the prevalence of Parkinsonism in Pakistan. *Neuropsychiatr Dis Treat*. 2016; 12: 1499-506.

	Benjamin-Chung J, Nazneen A, Halder AK, Haque R, Siddique A, Uddin MS, Koporc K, Arnold BF, Hubbard AE, Unicomb L, Luby SP, Addiss DG, Colford JM. The Interaction of Deworming, Improved Sanitation, and Household Flooring with Soil-Transmitted Helminth Infection in Rural Bangladesh. *PLoS Negl Trop Dis*. 2015; 9(12): e0004256.
	International Centre for Diarrhoeal Disease Research, Bangladesh. Seroprevalence of dengue virus infection in Dhaka, Bangladesh, 2012. *Health Sci Bull*. 2014; 12(2): 1-6.
	Alliance for Maternal and Newborn Health Improvement (AMANHI) mortality study group. Population-based rates, timing, and causes of maternal deaths, stillbirths, and neonatal deaths in south Asia and sub-Saharan Africa: a multi-country prospective cohort study. *Lancet Glob Health*. 6(12): e1297-e308.
	Jit M, Brisson M, Portnoy A, Hutubessy R. Cost-effectiveness of female human papillomavirus vaccination in 179 countries: a PRIME modelling study. *Lancet Glob Health*. 2014; 2(7): e406-14.
	MacDonald NE, Guichard S, Amarasinghe A, Balakrishnan MR, 2014 Inter-country SEAR Workshop Participants. Strengthening of causality assessment of adverse events following immunization in the WHO South East Asia and Western Pacific regions: Lessons from the 2014 SEAR inter-country workshop. *Vaccine*. 2015.
	Hoque M, Alam S, Ahmed A. Pattern of Neonatal Admissions and Outcome in an Intensive Care Unit (ICU) of a Tertiary Care Paediatric Hospital in Bangladesh - A One-Year Analysis. *J Bangladesh Coll Phys Surg*. 2013; 31(3): 134-9.
	Anand S, Khanam MA, Saquib J, Saquib N, Ahmed T, Alam DS, Cullen MR, Barry M, Chertow GM. High prevalence of chronic kidney disease in a community survey of urban Bangladeshis: a cross-sectional study. *Global Health*. 2014; 9.
	Hossain MS, Kypri K, Rahman B, Arslan I, Akter S, Milton AH. Prevalence and correlates of smokeless tobacco consumption among married women in rural Bangladesh. *PLoS One*. 2014; 9(1): e84470.
	Bull FC, Maslin TS, Armstrong T. Global physical activity questionnaire (GPAQ): nine country reliability and validity study. *J Phys Act Health*. 2009; 6(6): 790–804.
	Chan GJ, Baqui AH, Modak JK, Murillo-Chaves A, Mahmud AA, Boyd TK, Black RE, Saha SK. Early-onset neonatal sepsis in Dhaka, Bangladesh: risk associated with maternal bacterial colonisation and chorioamnionitis. *Trop Med Int Health*. 2013; 18(9): 1057-64.

	Chan GJ, Modak JK, Mahmud AA, Baqui AH, Black RE, Saha SK. Maternal and neonatal colonization in Bangladesh: prevalences, etiologies and risk factors. *J Perinatol*. 2013; 33(12): 9716.
	Islam MS, Sazzad HM, Satter SM, Sultana S, Hossain MJ, Hasan M, Rahman M, Campbell S, Cannon DL, Ströher U, Daszak P, Luby SP, Gurley ES. Nipah Virus Transmission from Bats to Humans Associated with Drinking Traditional Liquor Made from Date Palm Sap, Bangladesh, 2011-2014. *Emerg Infect Dis*. 2016; 22(4): 664-70.
	Murthy GV, Mactaggart I, Mohammad M, Islam J, Noe C, Khan AI, Foster A. Assessing the prevalence of sensory and motor impairments in childhood in Bangladesh using key informants. *Arch Dis Child*. 2014; 99(12): 1103-8.
	Chisti MJ, Graham SM, Duke T, Ahmed T, Ashraf H, Faruque AS, La Vincente S, Banu S, Raqib R, Salam MA. A prospective study of the prevalence of tuberculosis and bacteraemia in Bangladeshi children with severe malnutrition and pneumonia including an evaluation of Xpert MTB/RIF assay. *PLoS One*. 2014; 9(4): e93776.
	Saha S, Jhora ST, Talukder KA, Azmi IJ. Isolation and Characterization of Enteropathogenic Escherichia coli (EPEC) in Paediatric Diarrhoeal Patients by Detection of bfpA gene by PCR and HeLa cell Adherence Assay. *Mymensingh Med J*. 2015; 24(4): 800-5.
	Korpe PS, Haque R, Gilchrist C, Valencia C, Niu F, Lu M, Ma JZ, Petri SE, Reichman D, Kabir M, Duggal P, Petri WA. Natural History of Cryptosporidiosis in a Longitudinal Study of Slum-Dwelling Bangladeshi Children: Association with Severe Malnutrition. *PLoS Negl Trop Dis*. 2016; 10(5): e0004564.
	Sarkar SR, Hossain MA, Paul SK, Ray NC, Monwar S. Rotavirus is Predominent Enteropathogen in Acute Childhood Diarrhea in Mymensingh. *Mymensingh Med J*. 2015; 24(4): 665-70.
	Jewkes R, Fulu E, Roselli T, Garcia-Moreno C. Prevalence of and factors associated with non-partner rape perpetration: findings from the UN Multi-country Cross-sectional Study on Men and Violence in Asia and the Pacific. *Lancet Glob Health*. 2013; 1(4): e208-e218.
	Fulu E, Miedema S, Roselli T, McCook S, Chan KL, Haardörfer R, Jewkes R, UN Multi-country Study on Men and Violence study team. Pathways between childhood trauma, intimate partner violence, and harsh parenting: findings from the UN Multi-country Study on Men and Violence in Asia and the Pacific. *Lancet Glob Health*. 2017; 5(5): e512–22.

	Rahman MS, Akter S, Abe SK, Islam MR, Mondal MN, Rahman JA, Rahman MM. Awareness, treatment, and control of diabetes in Bangladesh: a nationwide population-based study. *PLoS One*. 2015; 10(2): e0118365.
	Bishwajit G, Yaya S, Seydou I. Diabetes mellitus and high blood pressure in relation to BMI among adult non-pregnant women in Bangladesh. *Diabetes Metab Syndr*. 2016.
	Nahar S, Afrad MH, Begum N, Al-Mamun F, Sarker AK, Das SK, Faruque ASG, Pourkarim MR, Choudhuri MSK, Azim T, Rahman M. High prevalence of noroviruses among hospitalized diarrheal patients in Bangladesh, 2011. *J Infect Dev Ctries*. 2013; 7(11): 892–6.
	Karageorgou D, Imamura F, Zhang J, Shi P, Mozaffarian D, Micha R. Assessing dietary intakes from household budget surveys: A national analysis in Bangladesh. *PLoS One*. 2018; 13(8): e0202831.
	Akter S, Rahman MM, Abe SK, Sultana P. Prevalence of diabetes and prediabetes and their risk factors among Bangladeshi adults: a nationwide survey. *Bull World Health Organ*. 2014; 92(3): 204–13.
	Hafiz I, Graves P, Haq R, Flora MS, Kelly-Hope LA. Clinical case estimates of lymphatic filariasis in an endemic district of Bangladesh after a decade of mass drug administration. *Trans R Soc Trop Med Hyg*. 2015; 109(11): 700-9.
	Biswas A, Rahman F, Eriksson C, Halim A, Dalal K. Facility Death Review of Maternal and Neonatal Deaths in Bangladesh. *PLoS One*. 2015; 10(11): e0141902.
	Colgate ER, Haque R, Dickson DM, Carmolli MP, Mychaleckyj JC, Nayak U, Qadri F, Alam M, Walsh MC, Diehl SA, Zaman K, Petri WA, Kirkpatrick BD. Delayed Dosing of Oral Rotavirus Vaccine Demonstrates Decreased Risk of Rotavirus Gastroenteritis Associated With Serum Zinc: A Randomized Controlled Trial. *Clin Infect Dis*. 2016; 63(5): 634-41.
	Owais A, Faruque ASG, Das SK, Ahmed S, Rahman S, Stein AD. Maternal and Antenatal Risk Factors for Stillbirths and Neonatal Mortality in Rural Bangladesh: A Case-Control Study. *PLoS One*. 2013; 8(11): e80164.
	Akhter A, Fatema K, Ahmed SF, Afroz A, Ali L, Hussain A. Prevalence and associated risk indicators of retinopathy in a rural Bangladeshi population with and without diabetes. *Ophthalmic Epidemiol*. 2013; 20(4): 220-7.
	Fulu E, Jewkes R, Roselli T, Garcia-Morena C, UN Multi-country Cross-sectional Study on Men and Violence research team. Prevalence of and factors associated with male perpetration of intimate partner violence: findings

	from the UN Multi-country Cross-sectional Study on Men and Violence in Asia and the Pacific. *Lancet Glob Health*. 2013; 1(4): e187-e207.
	Prost A, Colbourn T, Seward N, Azad K, Coomarasamy A, Copas A, Houweling TAJ, Fottrell E, Kuddus A, Lewycka S, MacArthur C, Manandhar D, Morrison J, Mwansambo C, Nair N, Nambiar B, Osrin D, Pagel C, Phiri T, Pulkki-Brännström A-M, Rosato M, Skordis-Worrall J, Saville N, More NS, Shrestha B, Tripathy P, Wilson A, Costello A. Women's groups practising participatory learning and action to improve maternal and newborn health in low-resource settings: a systematic review and meta-analysis. *Lancet*. 2013; 381(9879): 1736-46.
	Ferdous F, Ahmed S, Farzana FD, Das J, Malek MA, Das SK, Salam MA, Faruque ASG. Aetiologies of diarrhoea in adults from urban and rural treatment facilities in Bangladesh. *Epidemiol Infect*. 2015; 143(7): 1377–87.
	Rahman MA, Mahmood MA, Spurrier N, Rahman M, Choudhury SR, Leeder S. Why do Bangladeshi people use smokeless tobacco products?. *Asia Pac J Public Health*. 2015; 27(2): NP2197-209.
	Ahmed M, Abedin J, Alam KF, Al Mamun A, Paul RC, Rahman M, Iuliano AD, Sturm-Ramirez K, Parashar U, Luby SP, Gurley ES. Incidence of Acute Diarrhea-Associated Death among Children < 5 Years of Age in Bangladesh, 2010-12. *Am J Trop Med Hyg*. 2017; 1-12.
	Gilchrist CA, Petri SE, Schneider BN, Reichman DJ, Jiang N, Begum S, Watanabe K, Jansen CS, Elliott KP, Burgess SL, Ma JZ, Alam M, Kabir M, Haque R, Petri WA. Role of the Gut Microbiota of Children in Diarrhea Due to the Protozoan Parasite Entamoeba histolytica. *J Infect Dis*. 2016; 213(10): 1579-85.
	Saquib N, Khanam MA, Saquib J, Anand S, Chertow GM, Barry M, Ahmed T, Cullen MR. High prevalence of type 2 diabetes among the urban middle class in Bangladesh. *BMC Public Health*. 2013; 1032.
	Ishaque T, Islam MB, Ara G, Endtz HP, Mohammad QD, Jacobs BC, Islam Z. High mortality from Guillain-Barré syndrome in Bangladesh. *J Peripher Nerv Syst*. 2017; 22(2): 121–6.
	Fatema K, Abedin Z, Mansur A, Rahman F, Khatun T, Sumi N, Kobura K, Akter S, Ali L. Screening for chronic kidney diseases among an adult population. *Saudi J Kidney Dis Transpl*. 2013; 24(3): 534-41.
	Nasreen S, Luby SP, Brooks WA, Homaira N, Al Mamun A, Bhuiyan MU, Rahman M, Ahmed D, Abedin J, Rahman M, Alamgir AS, Fry AM, Streatfield PK, Rahman A, Bresee J, Widdowson MA, Azziz-Baumgartner E. Population-based incidence of severe acute respiratory virus infections among children aged <5 years in rural Bangladesh, June-October 2010. *PLoS One*. 2014; 9(2): e89978.

	Paul RC, Faruque AS, Alam M, Iqbal A, Zaman K, Islam N, Sobhan A, DAS SK, Malek MA, Qadri F, Cravioto A, Luby SP. Incidence of severe diarrhoea due to Vibrio cholerae in the catchment area of six surveillance hospitals in Bangladesh. *Epidemiol Infect*. 2016; 1440(5): 927-39.
	Das SK, Begum D, Ahmed S, Ferdous F, Farzana FD, Chisti MJ, Latham JR, Talukder KA, Rahman MM, Begum YA, Faruque ASG, Malek MA, Qadri F, Ahmed T, Alam N. Geographical diversity in seasonality of major diarrhoeal pathogens in Bangladesh observed between 2010 and 2012. *Epidemiol Infect*. 2014; 142(12): 2530–41.
	Das SK, Ahmed S, Ferdous F, Farzana FD, Chisti MJ, Latham JR, Talukder KA, Rahman M, Begum YA, Qadri F, Faruque AS, Ahmed T. Etiological diversity of diarrhoeal disease in Bangladesh. *J Infect Dev Ctries*. 2013; 7(12): 900-9.
	Halim A, Utz B, Biswas A, Rahman F, van den Broek N. Cause of and contributing factors to maternal deaths; a cross-sectional study using verbal autopsy in four districts in Bangladesh. *BJOG*. 2014; 86-94.
	Shaha M, Perveen I, Alamgir MJ, Masud MH, Rahman MH. Prevalence and risk factors for gastro-esophageal reflux disease in the North-Eastern part of Bangladesh. *Bangladesh Med Res Counc Bull*. 2012; 38(3): 108-13.
	Das, A. K., A. D. Harries, S. G. Hinderaker, et al. . Active and Passive Case Detection Strategies for the Control of Leishmaniasis in Bangladesh. *Public Health Action*. 1969; 4 no 1: 5–21.
	Biswas A, Rahman F, Halim A, Eriksson C, Dalal K. Maternal and Neonatal Death Review (MNDR): A Useful Approach to Identifying Appropriate and Effective Maternal and Neonatal Health Initiatives in Bangladesh. *Health*. 2014; 06(14): 1669-79.
	Saha AK, Mohanta MK. Bancroftian elephantiasis in Nilphamari, Bangladesh. *Mymensingh Med J*. 2011; 20(1): 40-4.
	Chakraborty A, Sazzad HM, Hossain MJ, Islam MS, Parveen S, Husain M, Banu SS, Podder G, Afroj S, Rollin PE, Daszak P, Luby SP, Rahman M, Gurley ES. Evolving epidemiology of Nipah virus infection in Bangladesh: evidence from outbreaks during 2010-2011. *Epidemiol Infect*. 2016; 144(2): 371-80.
	Islam MdN, Hossain M, Khaleque M, Das M, Khan M, Bari M, Bhuiyan M. Prevalence of Congenital Heart Disease in Neonate in a Tertiary Level Hospital. *Nepal J Med Sci*. 2013; 2(2): 91-5.
	Khatun J, Huda MM, Hossain MS, Presber W, Ghosh D, Kroeger A, Matlashewski G, Mondal D. Accelerated active case detection of visceral

	leishmaniasis patients in endemic villages of Bangladesh. *PLoS One*. 2014; 9(8): e103678.
	Muhit M, Minto H, Parvin A, Jadoon MZ, Islam J, Yasmin S, Khandaker G. Prevalence of refractive error, presbyopia, and unmet need of spectacle coverage in a northern district of Bangladesh: Rapid Assessment of Refractive Error study. *Ophthalmic Epidemiol*. 2018; 25(2): 126-132.
	Huda MM, Chowdhury R, Ghosh D, Dash AP, Bhattacharya SK, Mondal D. Visceral leishmaniasis-associated mortality in Bangladesh: a retrospective cross-sectional study. *BMJ Open*. 2014; 4(7): e005408.
	Fottrell E, Azad K, Kuddus A, Younes L, Shaha S, Nahar T, Aumon BH, Hossen M, Beard J, Hossain T, Pulkki-Brannstrom AM, Skordis-Worrall J, Prost A, Costello A, Houweling TA. The effect of increased coverage of participatory women's groups on neonatal mortality in Bangladesh: A cluster randomized trial. *JAMA Pediatr*. 2013; 167(9): 816-25.
	Bhowmik B, Munir SB, Ahmed KR, Siddiquee T, Diep LM, Wright E, Hassan Z, Debnath PR, Mahtab H, Azad Khan AK, Hussain A. Anthropometric indices of obesity and type 2 diabetes in Bangladeshi population: Chandra Rural Diabetes Study (CRDS). *Obes Res Clin Pract*. 2014; 8(3): e201–98.
	Rodrigues EG, Bellinger DC, Valeri L, Hasan MO, Quamruzzaman Q, Golam M, Kile ML, Christiani DC, Wright RO, Mazumdar M. Neurodevelopmental outcomes among 2- to 3-year-old children in Bangladesh with elevated blood lead and exposure to arsenic and manganese in drinking water. *Environ Health*. 2016; 15: 44.
	Litwak L, Goh SY, Hussein Z, Malek R, Prusty V, Khamseh ME. Prevalence of diabetes complications in people in type 2 diabetes mellitus and its association with baseline characteristics in the multinational A1chieve study . *Diabetol Metab Syndr*. 2013; 5(1): 57.
	Cravedi P, Sharma SK, Bravo RF, Islam N, Tchokhonelidze I, Ghimire M, Pahari B, Thapa S, Basnet A, Tataradze A, Tinatin D, Beglarishvili L, Fwu CW, Kopp JB, Eggers P, Ene-Iordache B, Carminati S, Perna A, Chianca A, Couser WG, Remuzzi G, Perico N. Preventing renal and cardiovascular risk by renal function assessment: insights from a cross-sectional study in low-income countries and the USA. *BMJ Open*. 2012; 2(5): e001357.
	Tanchangya J, Geater AF. Use of traditional cooking fuels and the risk of young adult cataract in rural Bangladesh: a hospital-based case-control study. *BMC Ophthalmol*. 2011; 11: 16.
	Haque U, Glass GE, Bomblies A, Hashizume M, Mitra D, Noman N, Haque W, Kabir MM, Yamamoto T, Overgaard HJ. Risk factors associated with clinical malaria episodes in Bangladesh: a longitudinal study. *Am J Trop Med Hyg*. 2013; 88(4): 727-32.

	Leung DT, Bogetz J, Itoh M, Ganapathi L, Pietroni MAC, Ryan ET, Chisti MJ. Factors associated with encephalopathy in patients with Salmonella enterica serotype Typhi bacteremia presenting to a diarrheal hospital in Dhaka, Bangladesh. *Am J Trop Med Hyg*. 2012; 86(4): 698-702.
	Starzengruber P, Swoboda P, Fuehrer HP, Khan WA, Hofecker V, Siedl A, Fally M, Graf O, Teja-Isavadharm P, Haque R, Ringwald P, Noedl H. Current status of artemisinin-resistant falciparum malaria in South Asia: a randomized controlled artesunate monotherapy trial in Bangladesh. *PLoS One*. 2012; 7(12).
	Banu S, Rahman MT, Uddin MKM, Khatun R, Ahmed T, Rahman MM, Husain MA, van Leth F. Epidemiology of tuberculosis in an urban slum of Dhaka City, Bangladesh. *PLoS One*. 2013; 8(10): e77721.
	Day LT, Hruschka D, Mussell F, Jeffers E, Saha SL, Alam S. Perinatal mortality associated with use of uterotonics outside of Comprehensive Emergency Obstetric and Neonatal Care: a cross-sectional study. *Reprod Health*. 2016; 13(1): 129.
	Sikder SS, Labrique AB, Ullah B, Ali H, Rashid M, Mehra S, Jahan N, Shamim AA, West KP, Christian P. Accounts of severe acute obstetric complications in rural Bangladesh. *BMC Pregnancy Childbirth*. 2011; 11: 76.
	Samad MS, Itoh M, Moji K, Hossain M, Mondal D, Alam MS, Kimura E. Enzyme-linked immunosorbent assay for the diagnosis of Wuchereria bancrofti infection using urine samples and its application in Bangladesh. *Parasitol Int*. 2013; 62(6): 564-7.
	Luby SP, Halder AK, Tronchet C, Akhter S, Bhuiya A, Johnston RB. Household characteristics associated with handwashing with soap in rural Bangladesh. *Am J Trop Med Hyg*. 2009; 81(5): 882-7.
	Bartlett E, Parr J, Lindeboom W, Khanam MA, Koehlmoos TP. Sources and prevalence of self-reported asthma diagnoses in adults in urban and rural settings of Bangladesh. *Glob Public Health*. 2013; 8(1): 79-89.
	Kim SY, Sweet S, Chang J, Goldie SJ. Comparative evaluation of the potential impact of rotavirus versus HPV vaccination in GAVI-eligible countries: a preliminary analysis focused on the relative disease burden. *BMC Infect Dis*. 2011; 11(174): 1-17.
	Nasrin D, Wu Y, Blackwelder WC, Farag TH, Saha D, Sow SO, Alonso PL, Breiman RF, Sur D, Faruque ASG, Zaidi AKM, Biswas K, Van Eijk AM, Walker DG, Levine MM, Kotloff KL. Health care seeking for childhood diarrhea in developing countries: evidence from seven sites in Africa and Asia. *Am J Trop Med Hyg*. 2013; 89(1 Suppl): 3-12.

	Gernand AD, Schulze KJ, Nanayakkara-Bind A, Arguello M, Shamim AA, Ali H, Wu L, West KP, Christian P. Effects of Prenatal Multiple Micronutrient Supplementation on Fetal Growth Factors: A Cluster-Randomized, Controlled Trial in Rural Bangladesh. *PLoS One*. 2015; 10(10): e0137269.
	Chowdhury S, Khan SU, Crameri G, Epstein JH, Broder CC, Islam A, Peel AJ, Barr J, Daszak P, Wang LF, Luby SP. Serological evidence of henipavirus exposure in cattle, goats and pigs in Bangladesh. *PLoS Negl Trop Dis*. 2014; 8(11): e3302.
	Prata N, Bell S, Quaiyum MA. Modeling maternal mortality in Bangladesh: the role of misoprostol in postpartum hemorrhage prevention. *BMC Pregnancy Childbirth*. 2014; 14(1): 78.
	Luby SP, Kadir MA, Yushuf Sharker MA, Yeasmin F, Unicomb L, Sirajul Islam M. A community-randomised controlled trial promoting waterless hand sanitizer and handwashing with soap, Dhaka, Bangladesh. *Trop Med Int Health*. 2010; 15(22): 1508-16.
	Mondal D, Minak J, Alam M, Liu Y, Dai J, Korpe P, Liu L, Haque R, Petri WA. Contribution of enteric infection, altered intestinal barrier function, and maternal malnutrition to infant malnutrition in Bangladesh. *Clin Infect Dis*. 2012; 54(2): 185–92.
	Rahman SN, Fatima P, Chowdhury AQ, Rahman MW. Blood level of lead in women with unexplained infertility. *Mymensingh Med J*. 2013; 22(3): 508-12.
	Lo MK, Lowe L, Hummel KB, Sazzad HM, Gurley ES, Hossain MJ, Luby SP, Miller DM, Comer JA, Rollin PE, Bellini WJ, Rota PA. Characterization of Nipah virus from outbreaks in Bangladesh, 2008-2010. *Emerg Infect Dis*. 2012; 18(2): 248-55.
	Mamun-Al-Mahtab null, Karim F, Foster G, Akbar SF, Rahman S. Prevalence and risk factors of asymptomatic hepatitis C virus infection in Bangladesh. *J Clin Exp Hepatol*. 2011; 1(1): 136.
	Chowdhury F, Rahman MA, Begum YA, Khan AI, Faruque ASG, Saha NC, Baby NI, Malek MA, Kumar AR, Svennerholm A-M, Pietroni M, Cravioto A, Qadri F. Impact of rapid urbanization on the rates of infection by Vibrio cholerae O1 and enterotoxigenic Escherichia coli in Dhaka, Bangladesh. *PLoS Negl Trop Dis*. 2011; 5(4): e999.
	Hirve S, Singh SP, Kumar N, Banjara MR, Das P, Sundar S, Rijal S, Joshi A, Kroeger A, Varghese B, Thakur CP, Huda MM, Mondal D. Effectiveness and feasibility of active and passive case detection in the visceral leishmaniasis elimination initiative in India, Bangladesh, and Nepal. *Am J Trop Med Hyg*. 2010; 83(3): 507-11.

	Khatun F, Rasheed S, Moran AC, Alam AM, Shomik MS, Sultana M, Choudhury N, Iqbal M, Bhuiya A. Causes of neonatal and maternal deaths in Dhaka slums: implications for service delivery. *BMC Public Health*. 2012; 12(84): 84.
	Gurley ES, Salje H, Homaira N, Ram PK, Haque R, Petri Jr. WA, Bresee J, Moss WJ, Luby SP, Breysse P, Azziz-Baumgartner E. Seasonal concentrations and determinants of indoor particulate matter in a low-income community in Dhaka, Bangladesh. *Environ Res*. 2013; 11-6.
	Taniuchi M, Sobuz SU, Begum S, Platts-Mills JA, Liu J, Yang Z, Wang XQ, Petri WA, Haque R, Houpt ER. Etiology of diarrhea in Bangladeshi infants in the first year of life analyzed using molecular methods. *J Infect Dis*. 2013; 208(11): 1794-802.
	Dey AC, Saha L, Shahidullah M. Risk factors, morbidity and mortality of neonatal tetanus. *Mymensingh Med J*. 2011; 20(1): 54-8.
	Nahar S, Rahman A, Nasreen HE. Factors Influencing Stillbirth in Bangladesh: A Case-Control Study. *Paediatr Perinat Epidemiol*. 2013; 27(2): 158-64.
	Yakoob MY, Theodoratou E, Jabeen A, Imdad A, Eisele TP, Ferguson J, Jhass A, Rudan I, Campbell H, Black RE, Bhutta ZA. Preventive zinc supplementation in developing countries: impact on mortality and morbidity due to diarrhea, pneumonia and malaria. *BMC Public Health*. 2011; S23.
	Dey SK, Chisti MJ, Das SK, Shaha CK, Ferdous F, Farzana FD, Ahmed S, Malek MA, Faruque ASG, Ahmed T, Salam MA. Characteristics of diarrheal illnesses in non-breast fed infants attending a large urban diarrheal disease hospital in Bangladesh. *PLoS One*. 2013; 8(3): e58228.
	Gurley ES, Hossain MJ, Paul RC, Sazzad HMS, Islam MS, Parveen S, Faruque LI, Husain M, Ara K, Jahan Y, Rahman M, Luby SP. Outbreak of hepatitis E in urban bangladesh resulting in maternal and perinatal mortality. *Clin Infect Dis*. 2014; 59(5): 658-65.
	Islam MS, Hossain MM, Pasha MM, Azad AK, Murshed KM. Prevalence and risk factors of chronic obstructive pulmonary disease (COPD) in Dhaka city population. *Mymensingh Med J*. 2013; 22(3): 547-51.
	Das SK, Ahmed S, Ferdous F, Farzana FD, Chisti MJ, Leung DT, Malek MA, Talukder KA, Bardhan PK, Salam MA, Faruque AS, Raqib R. Changing emergence of Shigella sero-groups in Bangladesh: observation from four different diarrheal disease hospitals. *PLoS One*. 2013; 8(4): e62029.
	Zaman K, Sack DA, Neuzil KM, Yunus M, Moulton LH, Sugimoto JD, Fleming JA, Hossain I, Arifeen SE, Azim T, Rahman M, Lewis KDC, Feller AJ, Qadri F, Halloran ME, Cravioto A, Victor JC. Effectiveness of a live oral human

	rotavirus vaccine after programmatic introduction in Bangladesh: A cluster-randomized trial. *PLoS Med*. 2017; 14(4): e1002282.
	Homaira N, Rahman M, Hossain MJ, Epstein JH, Sultana R, Khan MS, Podder G, Nahar K, Ahmed B, Gurley ES, Daszak P, Lipkin WI, Rollin PE, Comer JA, Ksiazek TG, Luby SP. Nipah virus outbreak with person-to-person transmission in a district of Bangladesh, 2007. *Epidemiol Infect*. 2010; 138(11): 1630-6.
	Chang S, El Arifeen S, Bari S, Wahed MA, Rahman KM, Rahman MT, Mahmud AB, Begum N, Zaman K, Baqui AH, Black RE. Supplementing iron and zinc: double blind, randomized evaluation of separate or combined delivery. *Eur J Clin Nutr*. 2010; 64(2): 153-60.
	Homaira N, Rahman M, Hossain MJ, Nahar N, Khan R, Rahman M, Podder G, Nahar K, Khan D, Gurley ES, Rollin PE, Comer JA, Ksiazek TG, Luby SP. Cluster of Nipah virus infection, Kushtia District, Bangladesh, 2007. *PLoS One*. 2010; 5(10): e13570.
	Karim R. Seroprevalence of Hepatitis B - surface antigen among non-professional blood donors in selected hospital in Dhaka city. *Retrovirology*. 2010; 7(S1): 52-53.
	Kotloff KL, Blackwelder WC, Nasrin D, Nataro JP, Farag TH, Eijk A van, Adegbola RA, Alonso PL, Breiman RF, Faruque ASG, Saha D, Sow SO, Sur D, Zaidi AKM, Biswas K, Panchalingam S, Clemens JD, Cohen D, Glass RI, Mintz ED, Sommerfelt H, Levine MM. The Global Enteric Multicenter Study (GEMS) of Diarrheal Disease in Infants and Young Children in Developing Countries: Epidemiologic and Clinical Methods of the Case/Control Study. *Clin Infect Dis*. 2012; 55 Suppl 4: S232-S245.
	Ferdous J, Ahmed A, Dasgupta SK, Jahan M, Huda FA, Ronsmans C, Koblinsky M, Chowdhury ME. Occurrence and determinants of postpartum maternal morbidities and disabilities among women in Matlab, Bangladesh. *J Health Popul Nutr*. 2012; 30(2): 143-158.
	Shaheen MM, Raquib A, Ahmad SM. Prevalence and associated socio-demographic factors of chronic suppurative otitis media among rural primary school children of Bangladesh. *Int J Pediatr Otorhinolaryngol*. 2012; 76(8): 1201-4.
	Khan M, Mustafa MG, Ahmad N, Alam MS, Baig RH, Chowdhry ZR, Ahmed M. Seroprevalence of hepatitis C virus in rural population of Bangladesh. *Indian J Gastroenterol*. 2010; 29(1): 38-9.
	Haque U, Ahmed SM, Hossain S, Huda M, Hossain A, Alam MS, Mondal D, Khan WA, Khalequzzaman M, Haque R. Malaria Prevalence in Endemic Districts of Bangladesh. *PLoS One*. 2009; 4(8): e6737.

	Begum S, Ahmed M, Sen B. Do Water and Sanitation Interventions Reduce Childhood Diarrhoea? New Evidence from Bangladesh. *Bangladesh Dev Stud*. 2012; 34(3): 1-30.
	Hossain S, Zaman K, Banu S, Quaiyum MA, Husain MA, Islam MA, Cooreman E, Borgdorff M, van Leth F. Tuberculin survey in Bangladesh, 2007–2009: prevalence of tuberculous infection and implications for TB control. *Int J Tuberc Lung Dis*. 2013; 17(10): 1267–72.
	Hossain S, Quaiyum MA, Zaman K, Banu S, Husain MA, Islam MA, Cooreman E, Borgdorff M, Lönnroth K, Salim AH, van Leth F. Socio economic position in TB prevalence and access to services: results from a population prevalence survey and a facility-based survey in Bangladesh. *PLoS One*. 2012; 7(9): e44980.
	Reid HL, Haque U, Roy S, Islam N, Clements ACA. Characterizing the spatial and temporal variation of malaria incidence in Bangladesh, 2007. *Malar J*. 2012; 170.
	Kotloff KL, Nataro JP, Blackwelder WC, Nasrin D, Farag TH, Panchalingam S, Wu Y, Sow SO, Sur D, Breiman RF, Faruque AS, Zaidi AK, Saha D, Alonso PL, Tamboura B, Sanogo D, Onwuchekwa U, Manna B, Ramamurthy T, Kanungo S, Ochieng JB, Omore R, Oundo JO, Hossain A, Das SK, Ahmed S, Qureshi S, Quadri F, Adegbola RA, Antonio M, Hossain MJ, Akinsola A, Mandomando I, Nhampossa T, Acácio S, Biswas K, O'Reilly CE, Mintz ED, Berkeley LY, Muhsen K, Sommerfelt H, Robins-Browne RM, Levine MM. Burden and aetiology of diarrhoeal disease in infants and young children in developing countries (the Global Enteric Multicenter Study, GEMS): a prospective, case-control study. *Lancet*. 2013; 382(9888): 209–22.
	Zaman K, Hossain S, Banu S, Quaiyum MA, Barua PC, Salim MAH, Begum V, Islam MA, Ahmed J, Rifat M, Cooreman E, Van Der Werf MJ, Borgdorff M, Van Leth F. Prevalence of smear-positive tuberculosis in persons aged ≥ 15 years in Bangladesh: results from a national survey, 2007-2009. *Epidemiol Infect*. 2012; 140(6): 1018-27.
	Homaira N, Rahman M, Hossain MJ, Khatun S, Nahar N, Podder G, Gurley ES, Ksiazek TG, Luby SP. Evidence of Person-to-Person Transmission of Nipah Virus Through Casual Contact. *Int J Infect Dis*. 2008; 12: e99.
	Mitra AK, Haque A, Islam M, Bashar SAMK. Lead poisoning: an alarming public health problem in Bangladesh. *Int J Environ Res Public Health*. 2009; 6(1): 84-95.
	Dey NC, Parvez M, Islam MR, Mistry SK, Levine DI. Effectiveness of a community-based water, sanitation, and hygiene (WASH) intervention in reduction of diarrhoea among under-five children: Evidence from a repeated cross-sectional study (2007-2015) in rural Bangladesh. *Int J Hyg Environ Health*. 2019; 222(8): 1098-1108.

	Mitra AK, Ahua E, Saha PK. Prevalence of and risk factors for lead poisoning in young children in Bangladesh. *J Health Popul Nutr*. 2012; 30(4): 404-9.
	Zaman K, Dang DA, Victor JC, Shin S, Yunus M, Dallas MJ, Podder G, Vu DT, Le TP, Luby SP, Le HT, Coia ML, Lewis K, Rivers SB, Sack DA, Schödel F, Steele AD, Neuzil KM, Ciarlet M. Efficacy of pentavalent rotavirus vaccine against severe rotavirus gastroenteritis in infants in developing countries in Asia: a randomised, double-blind, placebo-controlled trial. *Lancet*. 2010; 376(9741): 615-23.
	Nahar S, Mostafa OA, Farheen A. Verbal autopsy for neonatal mortality: a community-based study. *Med J Cairo Univ*. 2012; 80(2): 25-9.
	Huda FA, Ahmed A, Dasgupta SK, Jahan M, Ferdous J, Koblinsky M, Ronsmans C, Chowdhury ME. Profile of maternal and foetal complications during labour and delivery among women giving birth in hospitals in Matlab and Chandpur, Bangladesh. *J Health Popul Nutr*. 2012; 30(2): 131-42.
	Huda TM, Unicomb L, Johnston RB, Halder AK, Yushuf Sharker MA, Luby SP. Interim evaluation of a large scale sanitation, hygiene and water improvement programme on childhood diarrhea and respiratory disease in rural Bangladesh. *Soc Sci Med*. 2012; 75(4): 604-11.
	Shah R, Mullany LC, Darmstadt GL, Talukder RR, Rahman SM, Mannan I, Arifeen SE, Baqui AH, ProjAHNMo Study Group in Bangladesh. Neonatal Mortality Risks Among Preterm Births in a Rural Bangladeshi Cohort. *Paediatr Perinat Epidemiol*. 2014; 28(6): 510-20.
	Halder AK, Tronchet C, Akhter S, Bhuiya A, Johnston R, Luby SP. Observed hand cleanliness and other measures of handwashing behavior in rural Bangladesh. *BMC Public Health*. 2010; 10: 545.
	Sikder SS, Labrique AB, Shamim AA, Ali H, Mehra S, Wu L, Shaikh S, West KP, Christian P. Risk factors for reported obstetric complications and near misses in rural northwest Bangladesh: analysis from a prospective cohort study. *BMC Pregnancy Childbirth*. 2014; 14: 347.
	Luby SP, Halder AK, Huda T, Unicomb L, Johnston RB. The effect of handwashing at recommended times with water alone and with soap on child diarrhea in rural Bangladesh: an observational study. *PLoS Med*. 2011; 8(6): e1001052.
	West KP, Shamim AA, Mehra S, Labrique AB, Ali H, Shaikh S, Klemm RDW, Wu LS-F, Mitra M, Haque R, Hanif AAM, Massie AB, Merrill RD, Schulze KJ, Christian P. Effect of maternal multiple micronutrient vs iron-folic acid supplementation on infant mortality and adverse birth outcomes in rural Bangladesh: the JiVitA-3 randomized trial. *JAMA*. 2014; 312(24): 2649-58.

	Moisi JC, Saha SK, Falade AG, Njanpop-Lafourcade B-M, Oundo J, Zaidi AKM, Afroj S, Bakare RA, Buss JK, Lasi R, Mueller J, Odekanmi AA, Sangare L, Scott JAG, Knoll MD, Levine OS, Gessner BD. Enhanced diagnosis of pneumococcal meningitis with use of the binax NOW immunochromatographic test of Streptococcus pneumoniae antigen: A multisite study. *Clin Infect Dis*. 2009; 48(Suppl 2): S49-S56.
	Mondal D, Singh SP, Kumar N, Joshi A, Sundar S, Das P, Siddhivinayak H, Kroeger A, Boelaert M. Visceral leishmaniasis elimination programme in India, Bangladesh, and Nepal: reshaping the case finding/case management strategy. *PLoS Negl Trop Dis*. 2009; 3(1): e355.
	Homaira N, Luby SP, Petri WA, Vainionpaa R, Rahman M, Hossain K, Snider CB, Rahman M, Alamgir ASM, Zesmin F, Alam M, Gurley ES, Zaman RU, Azim T, Erdman DD, Fry AM, Bresee J, Widdowson M-A, Haque R, Azziz-Baumgartner E. Incidence of respiratory virus-associated pneumonia in urban poor young children of Dhaka, Bangladesh, 2009-2011. *PLoS One*. 2012; 7(2): e32056.
	Dalal K, Rahman F, Jansson B. Wife abuse in rural Bangladesh. *J Biosoc Sci*. 2009; 41(5): 561-73.
	Haseen F, Chawdhury FA, Hossain ME, Huq M, Bhuiyan MU, Imam H, Rahman DM, Gazi R, Khan SI, Kelly R, Ahmed J, Rahman M. Sexually transmitted infections and sexual behaviour among youth clients of hotel-based female sex workers in Dhaka, Bangladesh. *Int J STD AIDS*. 2012; 23(8): 553-9.
	Azim T, Rahman M, Alam MS, Chowdhury IA, Khan R, Reza M, Rahman M, Chowdhury EI, Hanifuddin M, Rahman AS. Bangladesh moves from being a low-prevalence nation for HIV to one with a concentrated epidemic in injecting drug users. *Int J STD AIDS*. 2008; 19(5): 327-31.
	Fischer Walker CL, Baqui AH, Ahmed S, Zaman K, El Arifeen S, Begum N, Yunus M, Black RE, Caulfield LE. Low-dose weekly supplementation of iron and/or zinc does not affect growth among Bangladeshi infants. *Eur J Clin Nutr*. 2009; 63(1): 87-92.
	Morkrid K, Ali L, Hussain A. Risk factors and prevalence of diabetic peripheral neuropathy: A study of type 2 diabetic outpatients in Bangladesh. *Int J Diabetes Dev Ctries*. 2010; 30(1): 11–7.
	Ahmed S, Hussain M, Akhter S, Islam T, Ahmed SU, Kabir ML. Genotypes of rotavirus diarrhoea in a children hospital of Bangladesh. *Mymensingh Med J*. 2012; 21(3): 497-502.
	Chowdhury Z, Le LT, Al Masud A, Chang KC, Alauddin M, Hossain M, Zakaria ABM, Hopke PK. Quantification of Indoor Air Pollution from Using Cookstoves

	and Estimation of Its Health Effects on Adult Women in Northwest Bangladesh. *Aerosol Air Qual Res*. 2012; 12: 463-75.
	Saha SK, Saha S, Shakur S, Hanif M, Habib MA, Datta SK, Bock HL. Community-based cross-sectional seroprevalence study of hepatitis A in Bangladesh. *World J Gastroenterol*. 2009; 15(39): 4932-7.
	Stoltzfus RJ, Klemm R. Research, policy, and programmatic considerations from the Biomarkers Reflecting Inflammation and Nutritional Determinants of Anemia (BRINDA) project. *Am J Clin Nutr*. 2017; 106(Suppl 1): 428S-434S.
	Ahmed M, Munshi SU, Nessa A, Ullah MS, Tabassum S, Islam MN. High prevalence of hepatitis A virus antibody among Bangladeshi children and young adults warrants pre-immunization screening of antibody in HAV vaccination strategy. *Indian J Med Microbiol*. 2009; 27(1): 48-50.
	Azad K, Barnett S, Banerjee B, Shaha S, Khan K, Rego AR, Barua S, Flatman D, Pagel C, Prost A, Ellis M, Costello A. Effect of scaling up women's groups on birth outcomes in three rural districts in Bangladesh: a cluster-randomised controlled trial. *Lancet*. 2010; 375(9721): 1193-202.
	Wirth JP, Woodruff BA, Engle-Stone R, Namaste SM, Temple VJ, Petry N, Macdonald B, Suchdev PS, Rohner F, Aaron GJ. Predictors of anemia in women of reproductive age: Biomarkers Reflecting Inflammation and Nutritional Determinants of Anemia (BRINDA) project. *Am J Clin Nutr*. 2017; 106(Suppl 1): 416S-427S.
	Azim T, Chowdhury EI, Reza M, Faruque MO, Ahmed G, Khan R, Rahman M, Pervez MM, Jana S, Strathdee SA. Prevalence of infections, HIV risk behaviors and factors associated with HIV infection among male injecting drug users attending a needle/syringe exchange program in Dhaka, Bangladesh. *Subst Use Misuse*. 2008; 43(14): 2124-44.
	Rahman M, Tondel M, Ahmad SA, Chowdhury IA, Faruquee MH, Axelson O. Hypertension and arsenic exposure in Bangladesh. *Hypertension*. 1999; 33(1): 74-8.
	Yusuf S, Rangarajan S, Teo K, Islam S, Li W, Liu L, Bo J, Lou Q, Lu F, Liu T, Yu L, Zhang S, Mony P, Swaminathan S, Mohan V, Gupta R, Kumar R, Vijayakumar K, Lear S, Anand S, Wielgosz A, Diaz R, Avezum A, Lopez-Jaramillo P, Lanas F, Yusoff K, Ismail N, Iqbal R, Rahman O, Rosengren A, Yusufali A, Kelishadi R, Kruger A, Puoane T, Szuba A, Chifamba J, Oguz A, McQueen M, McKee M, Dagenais G. Cardiovascular risk and events in 17 low-, middle-, and high-income countries. *N Engl J Med*. 2014; 371(9.0): 818-27.
	Yusuf S, Joseph P, Rangarajan S, Islam S, Mente A, Hystad P, Brauer M, Kutty VR, Gupta R, Wielgosz A, AlHabib KF, Dans A, Lopez-Jaramillo P, Avezum A, Lanas F, Oguz A, Kruger IM, Diaz R, Yusoff K, Mony P, Chifamba J, Yeates K, Kelishadi R, Yusufali A, Khatib R, Rahman O, Zatonska K, Iqbal

	R, Wei L, Bo H, Rosengren A, Kaur M, Mohan V, Lear SA, Teo KK, Leong D, O'Donnell M, McKee M, Dagenais G. Modifiable risk factors, cardiovascular disease, and mortality in 155722 individuals from 21 high-income, middle-income, and low-income countries (PURE): a prospective cohort study. *Lancet*. 2019.
	Haq SA, Darmawan J, Islam MN, Uddin MZ, Das BB, Rahman F, Chowdhury MAJ, Alam MN, Mahmud TAK, Chowdhury MR, Tahir M. Prevalence of rheumatic diseases and associated outcomes in rural and urban communities in Bangladesh: a COPCORD study. *J Rheumatol*. 2005; 32(2): 348-53.
	Ashraf H, Alam NH, Rothermundt C, Brooks A, Bardhan P, Hossain L, Salam MA, Hassan MS, Beglinger C, Gyr N. Prevalence and risk factors of hepatitis B and C virus infections in an impoverished urban community in Dhaka, Bangladesh. *BMC Infect Dis*. 2010; 10(1): 208.
	Rasul CH, Hasan MA, Yasmin F. Outcome of neonatal hyperbilirubinemia in a tertiary care hospital in bangladesh. *Malays J Med Sci*. 2010; 17(2): 40-4.
	Raina P, Sohel N, Oremus M, Shannon H, Mony P, Kumar R, Li W, Wang Y, Wang X, Yusoff K, Yusuf R, Iqbal R, Szuba A, Oguz A, Rosengren A, Kruger A, Chifamba J, Mohammadifard N, Darwish EA, Dagenais G, Diaz R, Avezum A, Lopez-Jaramillo P, Seron P, Rangarajan S, Teo K, Yusuf S, PURE Investigators. Assessing global risk factors for non-fatal injuries from road traffic accidents and falls in adults aged 35-70years in 17 countries: a cross-sectional analysis of the Prospective Urban Rural Epidemiological (PURE) study. *Inj Prev*. 2016; 22(2): 92-8.
	Razzaque A, Nahar L, Abu Haider Mohammad Golam Mustafa null, Karar Zunaid Ahsan null, Mohammad Shafiqul Islam null, Yunus M. Sociodemographic differentials of selected noncommunicable diseases risk factors among adults in Matlab, Bangladesh: findings from a WHO STEPS survey. *Asia Pac J Public Health*. 2011; 23(2): 183–91.
	Shimada Y, Matsuoka Y. Analysis of indoor PM2.5 exposure in Asian countries using time use survey. *Sci Total Environ*. 2011; 409(24): 5243-52.
	Khanam MA, Lindeboom W, Razzaque A, Niessen L, Milton AH. Prevalence and determinants of pre-hypertension and hypertension among the adults in rural Bangladesh: findings from a community-based study. *BMC Public Health*. 2015; 15: 203.
	Luby SP, Rahman M, Hossain MJ, Blum LS, Husain MM, Gurley E, Khan R, Ahmed BN, Rahman S, Nahar N, Kenah E, Comer JA, Ksiazek TG. Foodborne transmission of Nipah virus, Bangladesh. *Emerg Infect Dis*. 2006; 12(12): 1888-94.
	Wadud Z, Kuper H, Polack S, Lindfield R, Akm MR, Choudhury KA, Lindfield T, Limburg H, Foster A. Rapid assessment of avoidable blindness and needs

	assessment of cataract surgical services in Satkhira District, Bangladesh. *Br J Ophthalmol*. 2006; 90(10): 1225-9.
	Ng N, Hakimi M, Van Minh H, Juvekar S, Razzaque A, Ashraf A, Masud Ahmed S, Kanungsukkasem U, Soonthornthada K, Huu Bich T. Prevalence of physical inactivity in nine rural INDEPTH Health and Demographic Surveillance Systems in five Asian countries. *Glob Health Action*. 2009.
	Klemm RDW, Labrique AB, Christian P, Rashid M, Shamim AA, Katz J, Sommer A, West KP Jr. Newborn vitamin A supplementation reduced infant mortality in rural Bangladesh. *Pediatrics*. 2008; 122(1): e242-250.
	Goldie SJ, Diaz M, Kim SY, Levin CE, Van Minh H, Kim JJ. Mathematical models of cervical cancer prevention in the Asia Pacific region. *Vaccine*. 2008; 26 Suppl 12: M17-29.
	Huu Bich T, Thi Quynh Nga P, Ngoc Quang L, Van Minh H, Ng N, Juvekar S, Razzaque A, Ashraf A, Masud Ahmed S, Soonthornthada K, Kanungsukkasem U. Patterns of alcohol consumption in diverse rural populations in the Asian Region. *Glob Health Action*. 2009.
	Engle-Stone R, Aaron GJ, Huang J, Wirth JP, Namaste SM, Williams AM, Peerson JM, Rohner F, Varadhan R, Addo OY, Temple V, Rayco-Solon P, Macdonald B, Suchdev PS. Predictors of anemia in preschool children: Biomarkers Reflecting Inflammation and Nutritional Determinants of Anemia (BRINDA) project. *Am J Clin Nutr*. 2017; 106(Suppl 1): 402S-15S.
	Goldie SJ, O'Shea M, Campos NG, Diaz M, Sweet S, Kim SY. Health and economic outcomes of HPV 16,18 vaccination in 72 GAVI-eligible countries. *Vaccine*. 2008; 26(32): 4080-93.
	Larson LM, Namaste SM, Williams AM, Engle-Stone R, Addo OY, Suchdev PS, Wirth JP, Temple V, Serdula M, Northrop-Clewes CA. Adjusting retinol-binding protein concentrations for inflammation: Biomarkers Reflecting Inflammation and Nutritional Determinants of Anemia (BRINDA) project. *Am J Clin Nutr*. 2017; 106(Suppl 1): 390S-401S.
	Rahman A, Moran A, Pervin J, Rahman A, Rahman M, Yeasmin S, Begum H, Rashid H, Yunus M, Hruschka D, Arifeen SE, Streatfield PK, Sibley L, Bhuiya A, Koblinsky M. Effectiveness of an integrated approach to reduce perinatal mortality: recent experiences from Matlab, Bangladesh. *BMC Public Health*. 2011; 11(1): 914.
	Ellis M, Azad K, Banerjee B, Shaha SK, Prost A, Rego AR, Barua S, Costello A, Barnett S. Intrapartum-Related Stillbirths and Neonatal Deaths in Rural Bangladesh: A Prospective, Community-Based Cohort Study. *Pediatrics*. 2011; 127(5): e1182-90.

	Baqui AH, El-Arifeen S, Darmstadt GL, Ahmed S, Williams EK, Seraji HR, Mannan I, Rahman SM, Shah R, Saha SK, Syed U, Winch PJ, Lefevre A, Santosham M, Black RE, Projahnmo Study Group. Effect of community-based newborn-care intervention package implemented through two service-delivery strategies in Sylhet district, Bangladesh: a cluster-randomised controlled trial. *Lancet*. 2008; 371(9628): 1936-44.
	Mei Z, Namaste SM, Serdula M, Suchdev PS, Rohner F, Flores-Ayala R, Addo OY, Raiten DJ. Adjusting total body iron for inflammation: Biomarkers Reflecting Inflammation and Nutritional Determinants of Anemia (BRINDA) project. *Am J Clin Nutr*. 2017; 106(Suppl 1): 383S-389S.
	Siddique AK, Ahmed S, Iqbal A, Sobhan A, Poddar G, Azim T, Sack DA, Rahman M, Sack RB. Epidemiology of rotavirus and cholera in children aged less than five years in rural Bangladesh. *J Health Popul Nutr*. 2011; 29(1): 1–8.
	Merrill RD, Burke RM, Northrop-Clewes CA, Rayco-Solon P, Flores-Ayala R, Namaste SM, Serdula MK, Suchdev PS. Factors associated with inflammation in preschool children and women of reproductive age: Biomarkers Reflecting Inflammation and Nutritional Determinants of Anemia (BRINDA) project. *Am J Clin Nutr*. 2017; 106(Suppl 1): 348S-358S.
	Rokonuzzaman SM, Bhuian MR, Ali MH, Paul GK, Khan MR, Mamun AA. Epidemiological study of gastro-esophageal reflux disease in rural population. *Mymensingh Med J*. 2011; 20(3): 463-71.
	Namaste SM, Rohner F, Huang J, Bhushan NL, Flores-Ayala R, Kupka R, Mei Z, Rawat R, Williams AM, Raiten DJ, Northrop-Clewes CA, Suchdev PS. Adjusting ferritin concentrations for inflammation: Biomarkers Reflecting Inflammation and Nutritional Determinants of Anemia (BRINDA) project. *Am J Clin Nutr*. 2017; 106(Suppl 1): 359S-371S.
	Rahman MM, Rahim MA, Nahar Q. Prevalence and risk factors of type 2 diabetes in an urbanizing rural community of Bangladesh. *Bangladesh Med Res Counc Bull*. 2007; 33(2): 48-54.
	Namaste SM, Aaron GJ, Varadhan R, Peerson JM, Suchdev PS; BRINDA Working Group. Methodologic approach for the Biomarkers Reflecting Inflammation and Nutritional Determinants of Anemia (BRINDA) project. *Am J Clin Nutr*. 2017; 106(Suppl 1): 333S-347S.
	Dewan AM, Corner R, Hashizume M, Ongee ET. Typhoid Fever and its association with environmental factors in the Dhaka Metropolitan Area of Bangladesh: a spatial and time-series approach. *PLoS Negl Trop Dis*. 2013; 7(1): e1998.

	Bermudez OI, Lividini K, Smitz M-F, Fiedler JL. Estimating micronutrient intakes from Household Consumption and Expenditures Surveys (HCES): an example from Bangladesh. *Food Nutr Bull*. 2012; 33(3 Suppl): S208-213.
	Rohner F, Namaste SM, Larson LM, Addo OY, Mei Z, Suchdev PS, Williams AM, Sakr Ashour FA, Rawat R, Raiten DJ, Northrop-Clewes CA. Adjusting soluble transferrin receptor concentrations for inflammation: Biomarkers Reflecting Inflammation and Nutritional Determinants of Anemia (BRINDA) project. *Am J Clin Nutr*. 2017; 106(Suppl 1): 372S-382S.
	Halder AK, Gurley ES, Naheed A, Saha SK, Brooks WA, Arifeen SE, Sazzad HMS, Kenah E, Luby SP. Causes of early childhood deaths in urban Dhaka, Bangladesh. *PLoS One*. 2009; 4(12): e8145.
	Yusuf HKM, Rahman AM, Chowdhury FP, Mohiduzzaman M, Banu CP, Sattar MA, Islam MN. Iodine deficiency disorders in Bangladesh, 2004-05: ten years of iodized salt intervention brings remarkable achievement in lowering goitre and iodine deficiency among children and women. *Asia Pac J Clin Nutr*. 2008; 17(4): 620-8.
	Mullick MSI, Goodman R. The prevalence of psychiatric disorders among 5-10 year olds in rural, urban and slum areas in Bangladesh: an exploratory study. *Soc Psychiatry Psychiatr Epidemiol*. 2005; 40(8): 663-71.
	Rahim MA, Azad Khan AK, Nahar Q, Ali SMK, Hussain A. Impaired fasting glucose and impaired glucose tolerance in rural population of Bangladesh. *Bangladesh Med Res Counc Bull*. 2010; 36(2): 47–51.
	Baqui AH, Ahmed S, El Arifeen S, Darmstadt GL, Rosecrans AM, Mannan I, Rahman SM, Begum N, Mahmud AB, Seraji HR, Williams EK, Winch PJ, Santosham M, Black RE, Projahnmo 1 Study Group. Effect of timing of first postnatal care home visit on neonatal mortality in Bangladesh: a observational cohort study. *BMJ*. 2009; 339: b2826.
	Brooks WA, Goswami D, Rahman M, Nahar K, Fry AM, Balish A, Iftekharuddin N, Azim T, Xu X, Klimov A, Bresee J, Bridges C, Luby S. Influenza is a major contributor to childhood pneumonia in a tropical developing country. *Pediatr Infect Dis J*. 2010; 29(3): 216–21.
	Montgomery JM, Hossain MJ, Gurley E, Carroll GD, Croisier A, Bertherat E, Asgari N, Formenty P, Keeler N, Comer J, Bell MR, Akram K, Molla AR, Zaman K, Islam MR, Wagoner K, Mills JN, Rollin PE, Ksiazek TG, Breiman RF. Risk factors for Nipah virus encephalitis in Bangladesh. *Emerg Infect Dis*. 2008; 14(10): 1526-32.
	West KP Jr, Christian P, Labrique AB, Rashid M, Shamim AA, Klemm RD, Massie AB, Mehra S, Schulze KJ, Ali H, Ullah B, Wu LS, Katz J, Banu H, Akhter HH, Sommer A. Effects of vitamin A or beta carotene supplementation

	on pregnancy-related mortality and infant mortality in rural Bangladesh: a cluster randomized trial. *JAMA*. 2011; 305(19): 1986-95.
	Stockman LJ, Brooks WA, Streatfield PK, Rahman M, Goswami D, Nahar K, Rahman MZ, Luby SP, Anderson LJ. Challenges to Evaluating Respiratory Syncytial Virus Mortality in Bangladesh, 2004-2008. *PLoS One*. 2013; 8(1): e53857.
	Khan MM, Aklimunnessa K, Kabir MA, Mori M. Tobacco consumption and its association with illicit drug use among men in Bangladesh. *Addiction*. 2006; 101(8): 1178-86.
	Haque R, Mondal D, Karim A, Molla IH, Rahim A, Faruque ASG, Ahmad N, Kirkpatrick BD, Houpt E, Snider C, Petri WA. Prospective Case-Control Study of the Association between Common Enteric Protozoal Parasites and Diarrhea in Bangladesh. *Clin Infect Dis*. 2009; 48(9): 1191–7.
	Wasserman GA, Liu X, Parvez F, Ahsan H, Factor-Litvak P, Kline J, van Geen A, Slavkovich V, Loiacono NJ, Levy D, Cheng Z, Graziano JH. Water arsenic exposure and intellectual function in 6-year-old children in Araihazar, Bangladesh. *Environ Health Perspect*. 2007; 115(2): 285-9.
	Wasserman GA, Liu X, Parvez F, Ahsan H, Levy D, Factor-Litvak P, Kline J, van Geen A, Slavkovich V, LoIacono NJ, Cheng Z, Zheng Y, Graziano JH. Water manganese exposure and children's intellectual function in Araihazar, Bangladesh. *Environ Health Perspect*. 2006; 114(1): 124-9.
	Arifeen SE, Rahman S, Rahman K.M, Rahman SM, Bari S, Naheed A, Mannan I, Seraji MHR, Hassan MS, Huda N, Siddik AU, Quasem I, Brooks WA, Sack D, Luby SP, Saha SK, Islam M, Fatima K, Al-Emran H, Ahmed NU, Baqui AH, Breiman RF. Invasive pneumococcal disease among children in rural Bangladesh: Results from a population-based surveillance. *Clin Infect Dis*. 2009; 48(Suppl 2): S103-S113.
	Gurley ES, Montgomery JM, Hossain MJ, Bell M, Azad AK, Islam MR, Molla MA, Carroll DS, Ksiazek TG, Rota PA, Lowe L, Comer JA, Rollin P, Czub M, Grolla A, Feldmann H, Luby SP, Woodward JL, Breiman RF. Person-to-person transmission of Nipah virus in a Bangladeshi community. *Emerg Infect Dis*. 2007; 13(7): 1031-7.
	Fatema N, Chowdhury R, Chowdhury L. Incidence of Congenital Heart Disease among Hospital Live Birth in a Tertiary Hospital of Bangladesh. *Cardiovasc J*. 2008; 1(1): 14-20.
	Kongsbak K, Wahed MA, Friis H, Thilsted SH. Acute-phase protein levels, diarrhoea, Trichuris trichiura and maternal education are predictors of serum retinol: a cross-sectional study of children in a Dhaka slum, Bangladesh. *Br J Nutr*. 2006; 96(4): 725-34.

	Gurley ES, Hossain MJ, Montgomery SP, Petersen LR, Sejvar JJ, Mayer LW, Whitney A, Dull P, Nahar N, Uddin AK, Rahman ME, Ekram AR, Luby SP, Breiman RF. Etiologies of bacterial meningitis in Bangladesh: results from a hospital-based study. *Am J Trop Med Hyg*. 2009; 81(3): 475-83.
	Alam DS, Jha P, Ramasundarahettige C, Streatfield PK, Niessen LW, Chowdhury MAH, Siddiquee AT, Ahmed S, Evans TG. Smoking-attributable mortality in Bangladesh: proportional mortality study. *Bull World Health Organ*. 2013; 91(10): 757-64.
	Storla DG, Rahim Z, Islam MA, Plettner S, Begum V, Mannsaaker T, Myrvang B, Bjune G, Dahle UR. Heterogeneity of Mycobacterium tuberculosis isolates in Sunamganj District, Bangladesh. *Scand J Infect Dis*. 2006; 38(8): 593–6.
	Labrique AB, Zaman K, Hossain Z, Saha P, Yunus M, Hossain A, Ticehurst J, Nelson KE. Population seroprevalence of hepatitis E virus antibodies in rural Bangladesh. *Am J Trop Med Hyg*. 2009; 81(5): 875-81.
	Salam A, Alim A, Noguchi T. Spousal abuse against women and its consequences on reproductive health: a study in the urban slums in Bangladesh. *Matern Child Health J*. 2006; 10(1): 83-94.
	icddr,b. Impact of Hepatitis B vaccination programme in Bangladesh. *Health Sci Bull*. 2013; 2(1).
	Chowdhury HR, Thompson S, Ali M, Alam N, Yunus M, Streatfield PK. Causes of Neonatal Deaths in a Rural Subdistrict of Bangladesh: Implications for Intervention. *J Health Popul Nutr*. 2010; 28(4): 375-82.
	Chowdhury ME, Botlero R, Koblinsky M, Saha SK, Dieltiens G, Ronsmans C. Determinants of reduction in maternal mortality in Matlab, Bangladesh: a 30-year cohort study. *Lancet*. 2007; 370(9595): 1320-8.
	Chowdhury EK, Arifeen S El, Rahman M, Hoque DE, Hossain MA, Begum K, Siddik A, Begum N, Sadeq-ur Rahman Q, Akter T, Haque TM, Al-Helal ZM, Baqui AH, Bryce J, Black RE. Care at first-level facilities for children with severe pneumonia in Bangladesh: a cohort study. *Lancet*. 2008; 372(9641): 822–30.
	Aaron GJ, Friesen VM, Jungjohann S, Garrett GS, Neufeld LM, Myatt M. Coverage of Large-Scale Food Fortification of Edible Oil, Wheat Flour, and Maize Flour Varies Greatly by Vehicle and Country but Is Consistently Lower among the Most Vulnerable: Results from Coverage Surveys in 8 Countries. *J Nutr*. 2017; 147(5): 984S-994S.
	Hystad P, Larkin A, Rangarajan S, PURE country investigators, Yusuf S, Brauer M. Outdoor fine particulate matter air pollution and cardiovascular

	disease: Results from 747 communities across 21 countries in the PURE Study [Unpublished].
	Mashreky SR, Shawon RA, Biswas A, Ferdoush J, Unjum A, Rahman AKMF. Changes in burn mortality in Bangladesh: Findings from Bangladesh Health and Injury Survey (BHIS) 2003 and 2016. *Burns*. 2018; 44(6): 1579-1584.
	Naheed A, Ram PK, Brooks WA, Hossain MA, Parsons MB, Talukder KA, Mintz E, Luby S, Breiman RF. Burden of typhoid and paratyphoid fever in a densely populated urban community, Dhaka, Bangladesh. *Int J Infect Dis*. 2010; 14(Suppl 3): e93-e99.
	Petry N, Olofin I, Hurrell RF, Boy E, Wirth JP, Moursi M, Angel MD, Rohner F. The Proportion of Anemia Associated with Iron Deficiency in Low, Medium, and High Human Development Index Countries: A Systematic Analysis of National Surveys. *Nutrients*. 2016; 8(11).
	Palmer K, Kabir ZN, Ahmed T, Hamadani JD, Cornelius C, Kivipelto M, Wahlin Å. Prevalence of dementia and factors associated with dementia in rural Bangladesh: data from a cross-sectional, population-based study. *Int Psychogeriatr*. 2014; 26(11): 1905–15.
	Mihrshahi S, Ichikawa N, Shuaib M, Oddy W, Ampon R, Dibley MJ, Kabir AKMI, Peat JK. Prevalence of exclusive breastfeeding in Bangladesh and its association with diarrhoea and acute respiratory infection: results of the multiple indicator cluster survey 2003. *J Health Popul Nutr*. 2007; 25(2): 195–204.
	Lear SA, Hu W, Rangarajan S, Gasevic D, Leong D, Iqbal R, Casanova A, Swaminathan S, Anjana RM, Kumar R, Rosengren A, Wei L, Yang W, Chuangshi W, Huaxing L, Nair S, Diaz R, Swidon H, Gupta R, Mohammadifard N, Lopez-Jaramillo P, Oguz A, Zatonska K, Seron P, Avezum A, Poirier P, Teo K, Yusuf S. The effect of physical activity on mortality and cardiovascular disease in 130 000 people from 17 high-income, middle-income, and low-income countries: the PURE study. *Lancet*. 2017; 390(10113): 2643-2654.
	Afrad MH, Hassan Z, Farjana S, Moni S, Barua S, Das SK, Faruque AS, Azim T, Rahman M. Changing profile of rotavirus genotypes in Bangladesh, 2006-2012. *BMC Infect Dis*. 2013; 320.
	Baqui AH, Zaman K, Persson LA, El Arifeen S, Yunus M, Begum N, Black RE. Simultaneous weekly supplementation of iron and zinc is associated with lower morbidity due to diarrhea and acute lower respiratory infection in Bangladeshi infants. *J Nutr*. 2003; 133(12): 4150-7.
	Rahman AE, Moinuddin M, Molla M, Worku A, Hurt L, Kirkwood B, Mohan SB, Mazumder S, Bhutta Z, Raza F, Mrema S, Masanja H, Kadobera D, Waiswa P, Bahl R, Zangenberg M, Muhe L, on behalf of the Persistent Diarrhoea

	Research Group. Childhood diarrhoeal deaths in seven low- and middle-income countries. *Bull World Health Organ*. 2014; 92(9): 664-71.
	Baqui AH, Choi Y, Williams EK, Arifeen SE, Mannan I, Darmstadt GL, Black RE. Levels, timing, and etiology of stillbirths in Sylhet district of Bangladesh. *BMC Pregnancy Childbirth*. 2011; 11: 25.
	Von Seidlein L, Kim DR, Ali M, Lee H, Wang X, Thiem VD, Canh DG, Chaicumpa W, Agtini MD, Hossain A, Bhutta ZA, Mason C, Sethabutr O, Talukder K, Nair GB, Deen JL, Kotloff K, Clemens J. A Multicentre Study of Shigella Diarrhoea in Six Asian Countries: Disease Burden, Clinical Manifestations, and Microbiology. *PLoS Med*. 2006; 3(9): e353.
	Sobsey MD, Handzel T, Venczel L. Chlorination and safe storage of household drinking water in developing countries to reduce waterborne disease. *Water Sci Technol*. 2003; 47(3): 221-8.
	Ahsan H, Chen Y, Parvez F, Zablotska L, Argos M, Hussain I, Momotaj H, Levy D, Cheng Z, Slavkovich V, van Geen A, Howe GR, Graziano JH. Arsenic exposure from drinking water and risk of premalignant skin lesions in Bangladesh: baseline results from the Health Effects of Arsenic Longitudinal Study. *Am J Epidemiol*. 2006; 163(12): 1138-48.
	Lindstrom E, Hossain MB, Lönnerdal B, Raqib R, El Arifeen S, Ekstrom E-C. Prevalence of anemia and micronutrient deficiencies in early pregnancy in rural Bangladesh, the MINIMat trial. *Acta Obstet Gynecol Scand*. 2011; 90(1): 47–56.
	Srivastava A. An overview of an indoor radon study carried out in dwellings in India and Bangladesh during the last decade using solid state nuclear track detectors. *J Environ Radioact*. 2005; 78(1): 113–21.
	Gamble MV, Ahsan H, Liu X, Factor-Litvak P, Ilievski V, Slavkovich V, Parvez F, Graziano JH. Folate and cobalamin deficiencies and hyperhomocysteinemia in Bangladesh. *Am J Clin Nutr*. 2005; 81(6): 1372-7.
	Chen Y, Graziano JH, Parvez F, Hussain I, Momotaj H, van Geen A, Howe GR, Ahsan H. Modification of risk of arsenic-induced skin lesions by sunlight exposure, smoking, and occupational exposures in Bangladesh. *Epidemiology*. 2006; 17(4): 459-67.
	Karim MF, Brunetti E, Rahman S, Budke CM, Ahsan ASMA, Al-Mahtab M, Zaki KMJ, Alam MJ, Akbar SMF, Jalil MA. Abdominal cystic echinococcosis in Bangladesh: a hospital-based study. *J Infect Dev Ctries*. 2015; 9(1): 70–5.
	Hopke PK, Cohen DD, Begum BA, Biswas SK, Ni B, Pandit GG, Santoso M, Chung YS, Rahman SA, Hamzah MS, Davy P, Markwitz A, Waheed S, Siddique N, Santos FL, Pabroa PC, Seneviratne MC, Wimolwattanapun W,

	Bunprapob S, Vuong TB, Duy Hien P, Markowicz A. Urban air quality in the Asian region. *Sci Total Environ*. 2008; 404(1): 103-12.
	Nessa K, Waris S-A, Sultan Z, Monira S, Hossain M, Nahar S, Rahman H, Alam M, Baatsen P, Rahman M. Epidemiology and etiology of sexually transmitted infection among hotel-based sex workers in Dhaka, Bangladesh. *J Clin Microbiol*. 2004; 42(2): 618-21.
	Sayeed MA, Mahtab H, Khanam PA, Latif ZA, Banu A, Khan AK. Prevalence of diabetes and impaired fasting glucose in urban population of Bangladesh. *Bangladesh Med Res Counc Bull*. 2007; 33(1): 1-12.
	Wasserman GA, Liu X, Parvez F, Ahsan H, Factor-Litvak P, van Geen A, Slavkovich V, LoIacono NJ, Cheng Z, Hussain I, Momotaj H, Graziano JH. Water arsenic exposure and children's intellectual function in Araihazar, Bangladesh. *Environ Health Perspect*. 2004; 112(13): 1329-33.
	Hussain A, Rahim MA, Azad Khan AK, Ali SM, Vaaler S. Type 2 diabetes in rural and urban population: diverse prevalence and associated risk factors in Bangladesh. *Diabet Med*. 2005; 22(7): 931-6.
	Rahman SM, Åkesson A, Kippler M, Grandér M, Hamadani JD, Streatfield PK, Persson L-Å, Arifeen SE, Vahter M. Elevated Manganese Concentrations in Drinking Water May Be Beneficial for Fetal Survival. *PLoS One*. 2013; 8(9): e74119.
	Abu Sayeed M, Mahtab H, Akter Khanam P, Abul Ahsan K, Banu A, Rashid AN, Azad Khan AK. Diabetes and impaired fasting glycemia in the tribes of Khagrachari hill tracts of Bangladesh. *Diabetes Care*. 2004; 27(5): 1054-9.
	Qadri F, Saha A, Ahmed T, Al Tarique A, Begum YA, Svennerholm AM. Disease burden due to enterotoxigenic Escherichia coli in the first 2 years of life in an urban community in Bangladesh. *Infect Immun*. 2007; 75(8): 3961-8.
	Shamputa IC, Rigouts L, Eyongeta LA, El Aila NA, van Deun A, Salim AH, Willery E, Locht C, Supply P, Portaels F. Genotypic and phenotypic heterogeneity among Mycobacterium tuberculosis isolates from pulmonary tuberculosis patients. *J Clin Microbiol*. 2004; 42(12): 5528–36.
	Haq SA, Darmawan J, Islam MN, Ahmed M, Banik SK, Fazlur Rahman AKM, Alam MN, Tahir M, Rasker JJ. Incidence of musculoskeletal pain and rheumatic disorders in a Bangladeshi rural community: a WHO-APLAR-COPCORD study. *Int J Rheum Dis*. 2008; 11(3): 216-23.
	Mohammad QD, Habib M, Hoque A, Alam B, Haque B, Hossain S, Rahman KM, Khan SU. Prevalence of stroke above forty years. *Mymensingh Med J*. 2011; 20(4): 640-4.

	Hossain MJ, Gurley ES, Montgomery JM, Bell M, Carroll DS, Hsu VP, Formenty P, Croisier A, Bertherat E, Faiz MA, Azad AK, Islam R, Molla MA, Ksiazek TG, Rota PA, Comer JA, Rollin PE, Luby SP, Breiman RF. Clinical presentation of nipah virus infection in Bangladesh. *Clin Infect Dis*. 2008; 46(7): 977-84.
	Moore SE, Fulford AJ, Wagatsuma Y, Persson LA, Arifeen SE, Prentice AM. Thymus development and infant and child mortality in rural Bangladesh. *Int J Epidemiol*. 2014; 43(1): 216-23.
	Hsu VP, Hossain MJ, Parashar UD, Ali MM, Ksiazek TG, Kuzmin I, Niezgoda M, Rupprecht C, Bresee J, Breiman RF. Nipah virus encephalitis reemergence, Bangladesh. *Emerg Infect Dis*. 2004; 10(12): 2082-7.
	Kamal N, Joarder AH, Chowdhury AA, Khan AW. Prevalence of chronic suppurative otitis media among the children living in two selected slums of Dhaka City. *Bangladesh Med Res Counc Bull*. 2004; 30(3): 95-104.
	Hamid Salim MA, Declercq E, Van Deun A, Saki KAR. Gender differences in tuberculosis: a prevalence survey done in Bangladesh. *Int J Tuberc Lung Dis*. 2004; 8(8): 952-7.
	Biswas AC, Joarder AH, Siddiquee BH. Prevalence of CSOM among rural school going children. *Mymensingh Med J*. 2005; 14(2): 152-5.
	Sarkar NR, Anwar KS, Biswas KB, Mannan MA. Effect of deworming on nutritional status of ascaris infested slum children of Dhaka, Bangladesh. *Indian Pediatr*. 2002; 39(11): 1021-6.
	Alam DS, Chowdhury MAH, Siddiquee AT, Ahmed S, Hossain MD, Pervin S, Streatfield K, Cravioto A, Niessen LW. Adult Cardiopulmonary Mortality and Indoor Air Pollution: A 10-Year Retrospective Cohort Study in a Low-Income Rural Setting. *Glob Heart*. 2012; 7(3): 215–21.
	Luby SP, Hossain MJ, Gurley ES, Ahmed BN, Banu S, Khan SU, Homaira N, Rota PA, Rollin PE, Comer JA, Kenah E, Ksiazek TG, Rahman M. Recurrent zoonotic transmission of Nipah virus into humans, Bangladesh, 2001-2007. *Emerg Infect Dis*. 2009; 15(8): 1229-35.
	Karim MJ, Haq R, Mableson HE, Sultan Mahmood ASM, Rahman M, Chowdhury SM, Rahman AKMF, Hafiz I, Betts H, Mackenzie C, Taylor MJ, Kelly-Hope LA. Developing the first national database and map of lymphatic filariasis clinical cases in Bangladesh: Another step closer to the elimination goals. *PLoS Negl Trop Dis*. 2019; 13(7): e0007542.
	Zaman K, Yunus M, Arifeen SE, Baqui AH, Sack DA, Hossain S, Rahim Z, Ali M, Banu S, Islam MA, Begum N, Begum V, Breiman RF, Black RE.

	Prevalence of sputum smear-positive tuberculosis in a rural area in Bangladesh. *Epidemiol Infect*. 2006; 134(5): 1052-9.
	Srivastava A, Zaman MR, Dwivedi KK, Ramachandran TV. Indoor radon level in the dwellings of the Rajshahi and Chuadanga regions of Bangladesh. *Radiat Meas*. 2001; 34(1): 497–9.
	Arifeen SE, Hoque DME, Akter T, Rahman M, Hoque ME, Begum K, Chowdhury EK, Khan R, Blum LS, Ahmed S, Hossain MA, Siddik A, Begum N, Sadeq-ur Rahman Q, Haque TM, Billah SM, Islam M, Rumi RA, Law E, Al-Helal ZAM, Baqui AH, Schellenberg J, Adam T, Moulton LH, Habicht J-P, Scherpbier RW, Victora CG, Bryce J, Black RE. Effect of the Integrated Management of Childhood Illness strategy on childhood mortality and nutrition in a rural area in Bangladesh: a cluster randomised trial. *Lancet*. 2009; 374(9687): 393-403.
	Khan NZ, Muslima H, Parveen M, Bhattacharya M, Begum N, Chowdhury S, Jahan M, Darmstadt GL. Neurodevelopmental outcomes of preterm infants in Bangladesh. *Pediatrics*. 2006; 118(1): 280-9.
	Labrique AB, Sikder SS, Wu L, Rashid M, Ali H, Ullah B, Shamim AA, Mehra S, Klemm R, Banu H, West KP, Christian P. Beyond pregnancy--the neglected burden of mortality in young women of reproductive age in Bangladesh: a prospective cohort study. *BJOG*. 2013; 120(9): 1085-9.
	Rahim Z, Zaman K, van der Zanden AGM, Möllers MJ, van Soolingen D, Raqib R, Zaman K, Begum V, Rigouts L, Portaels F, Rastogi N, Sola C. Assessment of population structure and major circulating phylogeographical clades of Mycobacterium tuberculosis complex in Bangladesh suggests a high prevalence of a specific subclade of ancient M. tuberculosis genotypes. *J Clin Microbiol*. 2007; 45(11): 3791–4.
	Labrique AB, Sikder SS, Krain LJ, West KP, Christian P, Rashid M, Nelson KE. Hepatitis E, a vaccine-preventable cause of maternal deaths. *Emerg Infect Dis*. 2012; 18(9): 1401-4.
	Khan AM, Faruque ASG, Hossain MS. Isolation of Vibrio cholerae from neonates admitted to an urban diarrhoeal diseases hospital in Bangladesh. *Ann Trop Paediatr*. 2005; 25(3): 179-82.
	Flora MS, Mascie-Taylor CG, Rahman M. Gender and locality differences in tobacco prevalence among adult Bangladeshis. *Tob Control*. 2009; 18(6): 445-50.
	Shamsuzzaman AKM, Haq R, Karim MJ, Azad MB, Mahmood ASMS, Khair A, Rahman MM, Hafiz I, Ramaiah KD, Mackenzie CD, Mableson HE, Kelly-Hope LA. The significant scale up and success of Transmission Assessment Surveys ñTASî for endgame surveillance of lymphatic filariasis in Bangladesh:

	One step closer to the elimination goal of 2020. *PLoS Negl Trop Dis*. 2017; 11(1): e0005340.
	Cherry N, Shaikh K, McDonald C, Chowdhury Z. Stillbirth in rural Bangladesh: arsenic exposure and other etiological factors: a report from Gonoshasthaya Kendra. *Bull World Health Organ*. 2008; 86(3): 172-7.
	Adjuik M, Smith T, Clark S, Todd J, Garrib A, Kinfu Y, Kahn K, Mola M, Ashraf A, Masanja H, Adazu K, Sacarlal J, Alam N, Marra A, Gbangou A, Mwageni E, Binka F. Cause-specific mortality rates in sub-Saharan Africa and Bangladesh. *Bull World Health Organ*. 2006; 84(4): 181-8.
	Chen Y, McClintock TR, Segers S, Parvez F, Islam T, Ahmed A, Rakibuz-Zaman M, Hasan R, Sarwar G, Ahsan H. Prospective investigation of major dietary patterns and risk of cardiovascular mortality in Bangladesh. *Int J Cardiol*. 2013; 167(4): 1495-501.
	Tarleton JL, Haque R, Mondal D, Shu J, Farr BM, Petri WA Jr. Cognitive effects of diarrhea, malnutrition, and Entamoeba histolytica infection on school age children in Dhaka, Bangladesh. *Am J Trop Med Hyg*. 2006; 74(3): 475-81.
	Begum A, Nilufar S, Akther K, Rahman A, Khatun F, Rahman M, Khatoon F. Prevalence of selected reproductive tract infections among pregnant women attending an urban maternal and childcare unit in Dhaka, Bangladesh. *J Health Popul Nutr*. 2003; 21(2): 112-6.
	Dineen BP, Bourne RR, Ali SM, Huq DM, Johnson GJ. Prevalence and causes of blindness and visual impairment in Bangladeshi adults: results of the National Blindness and Low Vision Survey of Bangladesh [Underlying data]. *Br J Ophthalmol*. 2003; 87(7): 820-8.
	Baqui AH, El Arifeen S, Saha SK, Persson L, Zaman K, Gessner BD, Moulton LH, Black RE, Santosham M. Effectiveness of Haemophilus influenzae type B conjugate vaccine on prevention of pneumonia and meningitis in Bangladeshi children: a case-control study. *Pediatr Infect Dis J*. 2007; 26(7): 565-71.
	Monawar Hosain GM, Chatterjee N, Ara N, Islam T. Prevalence, pattern and determinants of mental disorders in rural Bangladesh. *Public Health*. 2007; 121(1): 18-24.
	Mollah AH, Nahar N, Siddique MA, Anwar KS, Hassan T, Azam MG. Common transfusion-transmitted infectious agents among thalassaemic children in Bangladesh. *J Health Popul Nutr*. 2003; 21(1): 67-71.
	Yinon L, Chen Y, Parvez F, Bangalore S, Islam T, Ahmed A, Rakibuz-Zaman M, Hasan R, Sarwar G, Ahsan H. A prospective study of variability in systolic blood pressure and mortality in a rural Bangladeshi population cohort. *Prev Med*. 2013; 57(6): 807–12.

	Shafique S, Akhter N, Stallkamp G, de Pee S, Panagides D, Bloem MW. Trends of under- and overweight among rural and urban poor women indicate the double burden of malnutrition in Bangladesh. *Int J Epidemiol*. 2007; 36(2): 449-57.
	Osendarp SJM, Santosham M, Black RE, Wahed MA, van Raaij JMA, Fuchs GJ. Effect of zinc supplementation between 1 and 6 mo of life on growth and morbidity of Bangladeshi infants in urban slums. *Am J Clin Nutr*. 2002; 76(6): 1401-8.
	Brooks WA, Hossain A, Goswami D, Sharmeen AT, Nahar K, Alam K, Ahmed N, Naheed A, Nair GB, Luby S, Breiman RF. Bacteremic Typhoid Fever in Children in an Urban Slum, Bangladesh. *Emerg Infect Dis*. 2005; 11(2): 326-9.
	Argos M, Melkonian S, Parvez F, Rakibuz-Zaman M, Ahmed A, Chen Y, Ahsan H. A population-based prospective study of energy-providing nutrients in relation to all-cause cancer mortality and cancers of digestive organs mortality. *Int J Cancer*. 2013; 133(10): 2422-8.
	Northrop-Clewes CA, Rousham EK, Mascie-Taylor CN, Lunn PG. Anthelmintic treatment of rural Bangladeshi children: effect on host physiology, growth, and biochemical status. *Am J Clin Nutr*. 2000; 73(1): 53-60.
	Chen Y, Ge W, Parvez F, Bangalore S, Eunus M, Ahmed A, Islam T, Rakibuz-Zaman M, Hasan R, Argos M, Levy D, Sarwar G, Ahsan H. A prospective study of arm circumference and risk of death in Bangladesh. *Int J Epidemiol*. 2014; 43(4): 1187-96.
	Minamoto K, Mascie-Taylor CGN, Moji K, Karim E, Rahman M. Arsenic-contaminated water and extent of acute childhood malnutrition (wasting) in rural Bangladesh. *Environ Sci*. 2005; 12(5): 283–92.
	Hypertension Study Group. Prevalence, awareness, treatment and control of hypertension among the elderly in Bangladesh and India: a multicentre study. *Bull World Health Organ*. 2001; 79(6): 490-500.
	Chen Y, Wu F, Liu M, Parvez F, Slavkovich V, Eunus M, Ahmed A, Argos M, Islam T, Rakibuz-Zaman M, Hasan R, Sarwar G, Levy D, Graziano J, Ahsan H. A Prospective Study of Arsenic Exposure, Arsenic Methylation Capacity, and Risk of Cardiovascular Disease in Bangladesh. *Environ Health Perspect*. 2013; 121(7): 832-8.
	Chen Y, Factor-Litvak P, Howe GR, Parvez F, Ahsan H. Nutritional influence on risk of high blood pressure in Bangladesh: a population-based cross-sectional study. *Am J Clin Nutr*. 2006; 84(5): 1224–32.

	Wu F, Chen Y, Parvez F, Segers S, Argos M, Islam T, Ahmed A, Rakibuz-Zaman M, Hasan R, Sarwar G, Ahsan H. A Prospective Study of Tobacco Smoking and Mortality in Bangladesh. *PLoS One*. 2013; 8(3): e58516.
	Qutub M, Akhter J. Epidemiology of genital herpes (HSV-2) among brothel based female sex workers in Bangladesh. *Eur J Epidemiol*. 2003; 18(9): 903-5.
	Kaiser R, Henderson AK, Daley WR, Naughton M, Khan MH, Rahman M, Kieszak S, Rubin CH. Blood lead levels of primary school children in Dhaka, Bangladesh. *Environ Health Perspect*. 2001; 109(6): 563-6.
	Zaman K, Yunus M, Faruque AS, El Arifeen S, Hossain I, Azim T, Rahman M, Podder G, Roy E, Luby S, Sack DA. Surveillance of rotavirus in a rural diarrhoea treatment centre in Bangladesh, 2000-2006. *Vaccine*. 2009; 27 Suppl 5: F31-4.
	Sayeed MA, Mahtab H, Akter Khanam P, Abdul Latif Z, Keramat Ali SM, Banu A, Ahren B, Azad Khan AK. Diabetes and impaired fasting glycemia in a rural population of Bangladesh. *Diabetes Care*. 2003; 26(4): 1034-9.
	Colombara DV, Cowgill KD, Faruque AS. Risk factors for severe cholera among children under five in rural and urban Bangladesh, 2000-2008: a hospital-based surveillance study. *PLoS One*. 2013; 8(1): e54395.
	Chen Y, Wu F, Parvez F, Islam T, Ahmed A, Rakibuz-Zaman M, Hasan R, Argos M, Levy D, Sarwar G, Ahsan H. Betel quid use and mortality in Bangladesh: a cohort study. *Bull World Health Organ*. 2015; 93(10): 684–92.
	Wu J. Diarrheal diseases in rural Bangladesh: spatial-temporal patterns, risk factors and pathogen detection. *Diss Abstr Int*. 2011; 73(1).
	Heck JE, Marcotte EL, Argos M, Parvez F, Ahmed A, Islam T, Sarwar G, Hasan R, Ahsan H, Chen Y. Betel quid chewing in rural Bangladesh: prevalence, predictors and relationship to blood pressure. *Int J Epidemiol*. 2012; 41(2): 462-71.
	Asghar S, Khan AKA, Ali SMK, Sayeed MA, Bhowmik B, Diep ML, Shi Z, Hussain A. Incidence of diabetes in Asian-Indian subjects: a five year follow-up study from Bangladesh. *Prim Care Diabetes*. 2011; 5(2): 117-24.
	Mihrshahi S, Oddy WH, Peat JK, Kabir I. Association between infant feeding patterns and diarrhoeal and respiratory illness: a cohort study in Chittagong, Bangladesh. *Int Breastfeed J*. 2008; 3(28).
	Baqui AH, Rahman M, Zaman K, Arifeen S El, Chowdhury HR, Begum N, Bhattacharya G, Chotani RA, Yunus M, Santosham M, Black RE. A

	population-based study of hospital admission incidence rate and bacterial aetiology of acute lower respiratory infections in children aged less than five years in Bangladesh. *J Health Popul Nutr*. 2007; 25(2): 179–88.
	Mondal D, Petri WA, Sack RB, Kirkpatrick BD, Haque R. Entamoeba histolytica-associated diarrheal illness is negatively associated with the growth of preschool children: evidence from a prospective study. *Trans R Soc Trop Med Hyg*. 2006; 100(11): 1032–8.
	Rahman MM, Tofail F, Wahed MA, Fuchs GJ, Baqui AH, Alvarez JO. Short-term supplementation with zinc and vitamin A has no significant effect on the growth of undernourished Bangladeshi children. *Am J Clin Nutr*. 2002; 75(1): 87-91.
	Saha SK, Darmstadt GL, Hanif M, Khan R. Seroepidemiology of varicella-zoster virus in Bangladesh. *Ann Trop Paediatr*. 2002; 22(4): 341-5.
	Brooks WA, Santosham M, Naheed A, Goswami D, Wahed MA, Diener-West M, Faruque ASG, Black RE. Effect of weekly zinc supplements on incidence of pneumonia and diarrhoea in children younger than 2 years in an urban, low-income population in Bangladesh: randomised controlled trial. *Lancet*. 2005; 366(9490): 999-1004.
	Hussain A, Vaaler S, Sayeed MA, Mahtab H, Ali SM, Khan AK. Type 2 diabetes and impaired fasting blood glucose in rural Bangladesh: a population-based study. *Eur J Public Health*. 2007; 17(3): 291-6.
	Adjuik M, Smith T, Clark S, Todd J, Garrib A, Kinfu Y, Kahn K, Mola M, Ashraf A, Masanja H, Adazu K, Sacarlal J, Alam N, Marra A, Gbangou A, Mwageni E, Binka F. Cause-specific mortality rates in sub-Saharan Africa and Bangladesh [Unpublished data]. *Bull World Health Organ*. 2006; 84(4): 181-8.
	Haque R, Mondal D, Kirkpatrick BD, Akther S, Farr BM, Sack RB, Petri WA Jr. Epidemiologic and clinical characteristics of acute diarrhea with emphasis on Entamoeba histolytica infections in preschool children in an urban slum of Dhaka, Bangladesh. *Am J Trop Med Hyg*. 2003; 69(4): 398-405.
	Bhowmik B, Afsana F, My Diep L, Binte Munir S, Wright E, Mahmood S, Khan AKA, Hussain A. Increasing prevalence of type 2 diabetes in a rural bangladeshi population: a population based study for 10 years. *Diabetes Metab*. 2013; 37(1): 46-53.
	Rahim MA, Hussain A, Azad Khan AK, Sayeed MA, Keramat Ali SM, Vaaler S. Rising prevalence of type 2 diabetes in rural Bangladesh: a population based study. *Diabetes Res Clin Pract*. 2007; 77(2): 300-5.

	Banu S, Gordon SV, Palmer S, Islam MR, Ahmed S, Alam KM, Cole ST, Brosch R. Genotypic analysis of Mycobacterium tuberculosis in Bangladesh and prevalence of the Beijing strain. *J Clin Microbiol*. 2004; 42(2): 674–82.
	Persson V, Ahmed F, Gebre-Medhin M, Greiner T. Increase in serum beta-carotene following dark green leafy vegetable supplementation in Mebendazole-treated school children in Bangladesh. *Eur J Clin Nutr*. 2001; 55(1): 1-9.
	Rahman M, Alam A, Nessa K, Hossain A, Nahar S, Datta D, Alam Khan S, Amin Mian R, Albert MJ. Etiology of sexually transmitted infections among street-based female sex workers in Dhaka, Bangladesh. *J Clin Microbiol*. 2000; 38(3): 1244-6.
	Ahmed F, Mahmuda I, Sattar A, Akhtaruzzaman M. Anaemia and vitamin A deficiency in poor urban pregnant women of Bangladesh. *Asia Pac J Clin Nutr*. 2003; 12(4): 460-6.
	Persson V, Ahmed F, Gebre-Medhin M, Greiner T. Relationships between vitamin A, iron status and helminthiasis in Bangladeshi school children. *Public Health Nutr*. 2000; 3(1): 83-9.
	Zaman MM, Yoshiike N. Prevalence of overweight defined by body mass index in a rural adult population of Bangladesh. *J Health Popul Nutr*. 2003; 21(2): 162-3.
	Zaki H, Darmstadt GL, Baten A, Ahsan CR, Saha SK. Seroepidemiology of hepatitis B and delta virus infections in Bangladesh. *J Trop Pediatr*. 2003; 49(6): 371-4.
	Chowdhury F, Kuchta A, Khan AI, Faruque AS, Calderwood SB, Ryan ET, Qadri F. The increased severity in patients presenting to hospital with diarrhea in Dhaka, Bangladesh since the emergence of the hybrid strain of Vibrio cholerae O1 is not unique to cholera patients. *Int J Infect Dis*. 2015; 400: 9-14.
	Ekström E-C, Hyder SMZ, Chowdhury AMR, Chowdhury SA, Lönnerdal B, Habicht J-P, Persson LA. Efficacy and trial effectiveness of weekly and daily iron supplementation among pregnant women in rural Bangladesh: disentangling the issues. *Am J Clin Nutr*. 2002; 76(6): 1392-400.
	El Ayadi AM, Hill K, Langer A, Subramanian SV, McCormick M. Comparability of sociodemographic and pregnancy characteristics of pregnancy-related deaths identified via the sisterhood method versus the household/verbal autopsy method. *Int J Gynaecol Obstet*. 2015.

	Bhargava A, Bouis HE, Scrimshaw NS. Dietary intakes and socioeconomic factors are associated with the hemoglobin concentration of Bangladeshi women. *J Nutr*. 2001; 131(3): 758-64.
	Hall A, Anwar K, Tomkins A, Rahman L. The distribution of Ascaris lumbricoides in human hosts: a study of 1765 people in Bangladesh. *Trans R Soc Trop Med Hyg*. 1999; 93(5): 503-10.
	Rashid, H. 1986-1996: Bangladesh Renal Registry Report. *Bangladesh Renal J*. 2002; 21: 25-28.
	Mascie-Taylor CG, Alam M, Montanari RM, Karim R, Ahmed T, Karim E, Akhtar S. A study of the cost effectiveness of selective health interventions for the control of intestinal parasites in rural Bangladesh. *J Parasitol*. 1999; 85(1): 6-11.
	Zaman MM, Yoshiike N, Rouf MA, Syeed MH, Khan MR, Hague S, Mahtab H, Tanaka H. Cardiovascular risk factors: distribution and prevalence in a rural population of Bangladesh. *J Cardiovasc Risk*. 2001; 8(2): 103-8.
	Islam MM, Ali M, Ferroni P, Underwood P, Alam MF. Prevalence of psychiatric disorders in an urban community in Bangladesh. *Gen Hosp Psychiatry*. 2003; 25(5): 353-7.
	Rahman MR, Paul DC, Rashid M, Ghosh A, Bangali AM, Jalil MA, Faiz MA. A randomized controlled trial on the efficacy of alternative treatment regimens for uncomplicated falciparum malaria in a multidrug-resistant falciparum area of Bangladesh--narrowing the options for the National Malaria Control Programme. *Trans R Soc Trop Med Hyg*. 2001; 95(6): 661-7.
	Razzaque A, Da Vanzo J, Rahman M, Gausia K, Hale L, Khan MA, Mustafa AHMG. Pregnancy spacing and maternal morbidity in Matlab, Bangladesh. *Int J Gynaecol Obstet*. 2005; 89(Suppl 1): S41-49.
	Faruque ASG, Malek MA, Khan AI, Huq S, Salam MA, Sack DA. Diarrhoea in elderly people: aetiology, and clinical characteristics. *Scand J Infect Dis*. 2004; 36(3): 204-8.
	Leung DT, Das SK, Malek MA, Ahmed D, Khanam F, Qadri F, Faruque AS, Ryan ET. Non-typhoidal Salmonella gastroenteritis at a diarrheal hospital in Dhaka, Bangladesh, 1996-2011. *Am J Trop Med Hyg*. 2013; 88(4): 661-9.
	Khanum H, Chawdhury S, Bhuiyan ZJ. Infestation of three intestinal worms in children of three selected rural areas, Bangladesh. *Pak J Zool*. 1999; 31(4): 391–6.

	Yusuf HR, Akhter HH, Rahman MH, Chowdhury ME, Rochat RW. Injury-related deaths among women aged 10-50 years in Bangladesh, 1996-97. *Lancet*. 2000; 355(9211): 1220-4.
	Leung DT, Das SK, Malek MA, Qadri F, Faruque ASG, Ryan ET. Impact of Ramadan on clinical and microbiologic parameters of patients seen at a diarrheal hospital in urban Dhaka, Bangladesh, 1996-2012. *Am J Trop Med Hyg*. 2014; 90(2): 294–8.
	Bogaerts J, Ahmed J, Akhter N, Begum N, Rahman M, Nahar S, Van Ranst M, Verhaegen J. Sexually transmitted infections among married women in Dhaka, Bangladesh: unexpected high prevalence of herpes simplex type 2 infection. *Sex Transm Infect*. 2001; 77(2): 114-9.
	Ziauddin Hyder S, Persson Lk, Chowdhury A, Ekström EC. Anaemia among non-pregnant women in rural Bangladesh. *Public Health Nutr*. 2001; 4(1): 79-83.
	Ahmed F, Khan MR, Islam M, Kabir I, Fuchs GJ. Anaemia and iron deficiency among adolescent schoolgirls in peri-urban Bangladesh. *Eur J Clin Nutr*. 2000; 54(9): 678-83.
	Sayeed MA, Hussain MZ, Banu A, Rumi MA, Azad Khan AK. Prevalence of diabetes in a suburban population of Bangladesh. *Diabetes Res Clin Pract*. 1997; 34(3): 149-55.
	Kolsteren P, Rahman SR, Hilderbrand K, Diniz A. Treatment for iron deficiency anaemia with a combined supplementation of iron, vitamin A and zinc in women of Dinajpur, Bangladesh. *Eur J Clin Nutr*. 1999; 53(2): 102-6.
	Sheikh A, Sugitani M, Kinukawa N, Moriyama M, Arakawa Y, Komiyama K, Li T-C, Takeda N, Ishaque SM, Hasan M, Suzuki K. Hepatitis e virus infection in fulminant hepatitis patients and an apparently healthy population in Bangladesh. *Am J Trop Med Hyg*. 2002; 66(6): 721-4.
	McCord C, Chowdhury Q. A cost effective small hospital in Bangladesh: what it can mean for emergency obstetric care. *Int J Gynaecol Obstet*. 2003; 81(1): 83-92.
	CHOICE Study Group. Multicenter, randomized, double-blind clinical trial to evaluate the efficacy and safety of a reduced osmolarity oral rehydration salts solution in children with acute watery diarrhea. *Pediatrics*. 2001; 107(4): 613-8.
	Rahman S, Hilderbrand K, Kolsteren P, Diniz A. A nutritional profile of non-pregnant women from the slums of Dinajpur, Bangladesh. *Trop Doct*. 1999; 29(4): 221-4.

	Bern C, Chowdhury R. The epidemiology of visceral leishmaniasis in Bangladesh: prospects for improved control. *Indian J Med Res*. 2006; 123(3): 275-88.
	Hussain F, Bhuiyan AB, Haque YA, Flora MS. Verbal autopsy for maternal death. *Bangladesh Med Res Counc Bull*. 2002; 28(1): 45-53.
	Nizam S, Khalequzzaman M, Yatsuya H, Khanam PA, Sayeed MA, Naito H, Nakajima T. Incidence of young onset insulin-requiring diabetes mellitus among 18- to 30-year-olds in Dhaka, Bangladesh (1994-2003). *Nagoya J Med Sci*. 2012; 74(1-2): 149-56.
	Filteau SM, Sullivan KR, Anwar US, Anwar ZR, Tomkins AM. Iodine deficiency alone cannot account for goitre prevalence among pregnant women in Modhupur, Bangladesh. *Eur J Clin Nutr*. 1994; 48(4): 293-302.
	Arifeen S, Black RE, Antelman G, Baqui A, Caulfield L, Becker S. Exclusive breastfeeding reduces acute respiratory infection and diarrhea deaths among infants in Dhaka slums. *Pediatrics*. 2001; 108(4): E67.
	Rahim DA, Ali SM, Rabbani MG, Rahman MA. Analysis of psychiatric morbidity of outpatient children in Mitford Hospital, Dhaka. *Bangladesh Med Res Counc Bull*. 1997; 23(2): 60-2.
	Dhar SC, Ansari S, Saha M, Ahmad MM, Rahman MT, Hasan M, Khan AK. Gallstone disease in a rural Bangladeshi community. *Indian J Gastroenterol*. 2001; 20(6): 223-6.
	Yasmin S, Osrin D, Paul E, Costello A. Neonatal mortality of low-birth-weight infants in Bangladesh. *Bull World Health Organ.* 2001; 79(7): 608-14.
	Arifeen SE, Black RE, Caulfield LE, Antelman G, Baqui AH, Nahar Q, Alamgir S, Mahmud H. Infant growth patterns in the slums of Dhaka in relation to birth weight, intrauterine growth retardation, and prematurity. *Am J Clin Nutr*. 2000; 72(4): 1010-7.
	Mamun AA, Padmadas SS, Khatun M. Maternal health during pregnancy and perinatal mortality in Bangladesh: evidence from a large-scale community-based clinical trial. *Paediatr Perinat Epidemiol*. 2006; 20(6): 482–90.
	Molla A, Bari A, Greenough WB 3rd. Rice oral rehydration solution hastens recovery from dysentery. *J Diarrhoeal Dis Res*. 1995; 13(1): 8-11.
	Stoltzfus R, Chakraborty J, Rice A, Briere B, Francisco A. Plausible evidence of effectiveness of an iron supplementation pr ogramme for pregnant and post-partum women in rural Bangladesh. *Food Nutr Bull*. 1998; 197-204.

	Chowdhury R, Mondal D, Chowdhury V, Faria S, Alvar J, Nabi SG, Boelaert M, Dash AP. How far are we from visceral leishmaniasis elimination in Bangladesh? An assessment of epidemiological surveillance data. *PLoS Negl Trop Dis*. 2014; 8(8): e3020.
	Pathela P, Zahid Hasan K, Roy E, Huq F, Kasem Siddique A, Bradley Sack R. Diarrheal illness in a cohort of children 0-2 years of age in rural Bangladesh: I Incidence and risk factors. *Acta Paediatr*. 2006; 95(4): 430-7.
	Ahmed KR, Karim MN, Bhowmik B, Habib SH, Bukht MS, Ali L, Hussain A. Incidence of diabetic retinopathy in Bangladesh: a 15-year follow-up study. *J Diabetes*. 2012; 4(4): 386-91.
	Tanaka G, Faruque ASG, Luby SP, Malek MA, Glass RI, Parashar UD. Deaths from rotavirus disease in Bangladeshi children: estimates from hospital-based surveillance. *Pediatr Infect Dis J*. 2007; 26(11): 1014-8.
	Hasan K, Jolly P, Marquis G, Roy E, Podder G, Alam K, Huq F, Sack R. Viral etiology of pneumonia in a cohort of newborns till 24 months of age in Rural Mirzapur, Bangladesh. *Scand J Infect Dis*. 2006; 38(8): 690-5.
	Akbar SMF, Hossain M, Hossain Md F, et al. Seroepidemiolgy of hepatitis viruses of chronic liver diseases in bangladesh: High prevalence of hcv among blood donors and healthy person. *Hepatol Res*. 1997; 7(2): 113-120.
	Fronczak N, Antelman G, Moran AC, Caulfield LE, Baqui AH. Delivery-related complications and early postpartum morbidity in Dhaka, Bangladesh. *Int J Gynaecol Obstet*. 2005; 91(3): 271-278.
	Roy E, Hasan KZ, Haque F, Siddique AKM, Sack RB. Acute otitis media during the first two years of life in a rural community in Bangladesh: a prospective cohort study. *J Health Popul Nutr*. 2007; 25(4): 414-21.
	Liu L, Li Q, Lee RA, Friberg IK, Perin J, Walker N, Black RE. Trends in causes of death among children under 5 in Bangladesh, 1993-2004: an exercise applying a standardized computer algorithm to assign causes of death using verbal autopsy data. *Popul Health Metr*. 2011; 9: 43.
	Hasin A, Begum R, Khan MR, Ahmed F. Relationship between birth weight and biochemical measures of maternal nutritional status at delivery in Bangladeshi urban poors. *Int J Food Sci Nutr*. 1996; 47(3): 273-9.
	Baqui AH, Black RE, Arifeen SE, Hill K, Mitra SN, al Sabir A. Causes of childhood deaths in Bangladesh: results of a nationwide verbal autopsy study. *Bull World Health Organ*. 1998; 76(2): 161-71.

	Khanum S, Noor K, Kawser CA. Studies on congenital abnormalities and related risk factors. *Mymensingh Med J*. 2004; 13(2): 177-80.
	Albert MJ, Faruque AS, Faruque SM, Sack RB, Mahalanabis D. Case-control study of enteropathogens associated with childhood diarrhea in Dhaka, Bangladesh. *J Clin Microbiol*. 1999; 37(11): 3458-64.
	Islam S, Durkin MS, Zaman SS. Socioeconomic status and the prevalence of mental retardation in Bangladesh. *Ment Retard*. 1993; 31(6): 412-7.
	Khan NZ, Ferdous S, Munir S, Huq S, McConachie H. Mortality of urban and rural young children with cerebral palsy in Bangladesh. *Dev Med Child Neurol*. 1998; 40(11): 749-53.
	Hoque BA, Juncker T, Sack RB, Ali M, Aziz KM. Sustainability of a water, sanitation and hygiene education project in rural Bangladesh: a 5-year follow-up. *Bull World Health Organ*. 1996; 74(4): 431-7.
	Hussain A, Ali SM, Kvâle G. Determinants of mortality among children in the urban slums of Dhaka city, Bangladesh. *Trop Med Int Health*. 1999; 4(11): 758-64.
	Zaman S, Khan M, Alam K, Williams R. Primary hepatocellular carcinoma and viral hepatitis B and C infection in Bangladeshi subjects. *J Trop Med Hyg*. 1995; 98(1): 64-8.
	Chowdhury ME, Akhter HH, Chongsuvivatwong V, Geater AF. Neonatal mortality in rural Bangladesh: an exploratory study. *J Health Popul Nutr*. 2005; 23(1): 16-24.
	Ahmed F, Khan MR, Karim R, Taj S, Hyderi T, Faruque MO, Margetts BM, Jackson AA. Serum retinol and biochemical measures of iron status in adolescent schoolgirls in urban Bangladesh. *Eur J Clin Nutr*. 1996; 50(6): 346-51.
	Hall A, Nahar Q. Albendazole and infections with Ascaris lumbricoides and Trichuris trichiura in children in Bangladesh. *Trans R Soc Trop Med Hyg*. 1994; 88(1): 110-2.
	Durkin MS, Davidson LL, Hasan ZM, Hasan Z, Hauser WA, Khan N, Paul TJ, Shrout PE, Thorburn MJ, Zaman S. Estimates of the prevalence of childhood seizure disorders in communities where professional resources are scarce: results from Bangladesh, Jamaica and Pakistan. *Paediatr Perinat Epidemiol*. 1992; 6(2): 166-80.

	Hoque BA, Chakraborty J, Chowdhury JTA, Chowdhury UK, Ali M, Arifeen SE, Sack RB. Effects of environmental factors on child survival in Bangladesh: a case control study. *Public Health*. 1999; 113(2): 57–64.
	Ahsan H, Underwood P, Atkinson D. Smoking among male teenagers in Dhaka, Bangladesh. *Prev Med*. 1998; 27(1): 70-6.
	Khan AM, Hossain MS, Khan AI, Chisti MJ, Chowdhury F, Faruque ASG, Salam MA. Bacterial enteropathogens of neonates admitted to an urban diarrhoeal hospital in Bangladesh. *J Trop Pediatr*. 2009; 55(2): 122-4.
	Ahmed J, Mostafa Zaman M, Monzur Hassan MM. Prevalence of rheumatic fever and rheumatic heart disease in rural Bangladesh. *Trop Doct*. 2005; 35(3): 160-1.
	Lee JE, McLerran DF, Rolland B, Chen Y, Grant EJ, Vedanthan R, Inoue M, Tsugane S, Gao YT, Tsuji I, Kakizaki M, Ahsan H, Ahn YO, Pan WH, Ozasa K, Yoo KY, Sasazuki S, Yang G, Watanabe T, Sugawara Y, Parvez F, Kim DH, Chuang SY, Ohishi W, Park SK, Feng Z, Thornquist M, Boffetta P, Zheng W, Kang D, Potter J, Sinha R. Meat intake and cause-specific mortality: a pooled analysis of Asian prospective cohort studies. *Am J Clin Nutr*. 2013; 98(4): 1032-41.
	Baqui AH, Sabir AA, Begum N, Arifeen SE, Mitra SN, Black RE. Causes of childhood deaths in Bangladesh: an update. *Acta Paediatr*. 2001; 90(6): 682-90.
	Mulder-Sibanda M, Sibanda-Mulder FS. Prolonged breastfeeding in Bangladesh: indicators of inadequate feeding practices or mothers' response to children's poor health? *Public Health*. 1999; 113(2): 65-8.
	Kusiako T, Ronsmans C, Van der Paal L. Perinatal mortality attributable to complications of childbirth in Matlab, Bangladesh. *Bull World Health Organ*. 2000; 78(5): 621-7.
	Salway S, Nasim SM. Levels, Trends and Causes of Mortality in Children Below 5 Years of Age in Bangladesh: Findings from a national survey. *J Diarrhoeal Dis Res*. 1994; 12(3): 187-193.
	Unicomb LE, Kilgore PE, Faruque SG, Hamadani JD, Fuchs GJ, Albert MJ, Glass RI. Anticipating rotavirus vaccines: hospital-based surveillance for rotavirus diarrhea and estimates of disease burden in Bangladesh. *Pediatr Infect Dis J*. 1997; 16(10): 947-51.
	Mitra AK, Akramuzzaman SM, Fuchs GJ, Rahman MM, Mahalanabis D. Long-term oral supplementation with iron is not harmful for young children in a poor community of Bangladesh. *J Nutr*. 1997; 127(8): 1451-5.

	Oliwa JN, Karumbi JM, Marais BJ, Madhi SA, Graham SM. Tuberculosis as a cause or comorbidity of childhood pneumonia in tuberculosis-endemic areas: a systematic review. *Lancet Respir Med*. 2015; 3(3): 23543.
Survey (132)	ICF Macro, Mitra and Associates, National Institute of Population Research and Training (NIPORT). Bangladesh Demographic and Health Survey 2011-2012. Calverton, United States of America: ICF Macro.
	Center for Public Health Genomics, University of Virginia, Centers for Disease Control and Prevention (CDC), International Vaccine Institute, School of Medicine, University of Virginia, University of Vermont. Bangladesh - Dhaka Performance of Rotovirus and Oral Vaccines in Developing Countries Study 2011-2014.
	Ara R, Hoy D, Haq SA, Islam MN, Alam MZ, Shahin MA, Rahman M. Bangladesh Estimation of the Burden of Knee Osteoarthritis in a Rural Community 2011. [Unpublished].
	International Centre for Eye Health (ICEH). Bangladesh - Kushtia Rapid Assessment of Avoidable Blindness Survey 2011. Grootebroek, Netherlands: RAAB Repository, 2011.
	International Centre for Diarrhoeal Disease Research, Bangladesh (ICDDR,B). Bangladesh - Chandpur and Comilla District Verbal Autopsy Study 2011-2014.
	Gallup. Bangladesh World Poll 2011.
	International Centre for Diarrhoeal Disease Research, Bangladesh (ICDDR,B), National Institute of Cardiovascular Disease (Bangladesh). Bangladesh Risk of Acute Vascular Events (BRAVE) Tabulation on BMI by Age and Sex.
	Institute for Health Metrics and Evaluation (IHME), International Centre for Diarrhoeal Disease Research, Bangladesh (ICDDR,B), Melbourne School of Population and Global Health, University of Melbourne. Improving Methods to Measure Comparable Mortality by Cause - Gold Standard Verbal Autopsy Data 2011-2014. Seattle, United States of America: Institute for Health Metrics and Evaluation (IHME), 2020.
	International Centre for Diarrhoeal Disease Research, Bangladesh (ICDDR,B), Ministry of Health and Family Welfare (Bangladesh), Mitra and Associates, National Institute of Population Research and Training (NIPORT), United Nations Population Fund (UNFPA), United States Agency for International Development (USAID). Bangladesh Maternal Mortality and Health Care Survey 2016.
	CSF Global (Bangladesh), International Centre for Eye Health (ICEH). Bangladesh - Gazipur, Kishoreganj, and Cox's Bazar Districts Rapid

	Assessment of Avoidable Blindness 2010. Grootebroek, Netherlands: RAAB Repository.
	Bangladesh Bureau of Statistics (BBS). Bangladesh Labor Force Survey 2010. Dhaka, Bangladesh: Bangladesh Bureau of Statistics (BBS).
	Bangladesh Maternal Mortality and Health Care Survey 2010, BMMS 2010.
	Institute for Health Metrics and Evaluation (IHME), International Centre for Diarrhoeal Disease Research, Bangladesh (ICDDR,B), Research Institute for Tropical Medicine (Philippines), School of Population Health, University of Queensland (Australia). Improving Methods to Measure Comparable Mortality by Cause - Verbal Autopsy Timing Data 2010-2013. Seattle, United States of America: Institute for Health Metrics and Evaluation (IHME), 2018.
	Gallup. Bangladesh World Poll 2010.
	CSF Global (Bangladesh), International Centre for Eye Health (ICEH). Bangladesh - Narail and Jamalpur Districts Rapid Assessment of Avoidable Blindness 2010.
	Gallup. Bangladesh World Poll 2018.
	Gallup. Bangladesh World Poll 2017.
	Bangladesh Bureau of Statistics (BBS), CDC Foundation, Centers for Disease Control and Prevention (CDC), Johns Hopkins Bloomberg School of Public Health, Ministry of Health and Family Welfare (Bangladesh), RTI International, World Health Organization (WHO). Bangladesh Global Adult Tobacco Survey 2017.
	Gallup. Bangladesh World Poll 2016.
	Malaria Atlas Project Group. Multinational Annual Parasite Incidence Data, Personal Communication with Malaria Atlas Project Group 2016. [Unpublished].
	Directorate General of Health Services, Ministry of Health and Family Welfare (Bangladesh), Joint United Nations Program on HIV/AIDS (UNAIDS), Save the Children International. Bangladesh Mapping and Size Estimation of Key Populations Study 2015-2016.
	Center for Social and Market Research (CSMR) (Bangladesh), Directorate General of Health Services, Ministry of Health and Family Welfare (Bangladesh), United Nations Children's Fund (UNICEF), World Health

	Organization (WHO). Bangladesh EPI Coverage Evaluation Survey 2015-2016.
	Gallup. Bangladesh World Poll 2015.
	Bangladesh Bureau of Statistics (BBS), Centers for Disease Control and Prevention (CDC), Directorate General of Health Services, Ministry of Health and Family Welfare (Bangladesh), Global Fund to Fight Aids Tuberculosis and Malaria (GFATM), Institute of Tropical Medicine (Belgium), National Tuberculosis Control Program (NTP) (Bangladesh), Research Institute of Tuberculosis/Japan Anti-Tuberculosis Association (RIT/JATA), United States Agency for International Development (USAID). Bangladesh National Tuberculosis Prevalence Survey 2015-2016.
	Alam S. Bangladesh Annual Parasite Incidence Data, Personal Communication with R. Namgay 2018. [Unpublished].
	International Food Policy Research Institute (IFPRI). 2016. Bangladesh Integrated Household Survey (BIHS) 2015. Washington, DC: International Food Policy ResearchInstitute (IFPRI) [dataset]. http://dx.doi.org/10.7910/DVN/BXSYEL
	ICF International, Mitra and Associates, National Institute of Population Research and Training (NIPORT). Bangladesh Demographic and Health Survey 2014. Fairfax, United States of America: ICF International, 2015.
	International Centre for Diarrhoeal Disease Research, Bangladesh (ICDDR,B). Bangladesh - Dhaka Cohort Study of Cryptosporidiosis in Children 2014. [Unpublished].
	Centers for Disease Control and Prevention (CDC), World Health Organization (WHO). Bangladesh Global School-Based Student Health Survey 2014. Geneva, Switzerland: World Health Organization (WHO), 2019.
	Institute for Health Metrics and Evaluation (IHME), International Centre for Diarrhoeal Disease Research, Bangladesh (ICDDR,B). Bangladesh Gavi FCE Health Facility Survey 2014. Seattle, United States of America: Institute for Health Metrics and Evaluation (IHME), 2017.
	Institute for Health Metrics and Evaluation (IHME), International Centre for Diarrhoeal Disease Research, Bangladesh (ICDDR,B). Bangladesh Gavi FCE Patient Exit Interview Survey 2014. Seattle, United States of America: Institute for Health Metrics and Evaluation (IHME), 2017.
	Gallup. Bangladesh World Poll 2014.
	Institute for Health Metrics and Evaluation (IHME), International Centre for Diarrhoeal Disease Research, Bangladesh (ICDDR,B). Bangladesh Gavi FCE

	Measles-Rubella Pre-Vaccine Campaign Household Survey 2013. Seattle, United States of America: Institute for Health Metrics and Evaluation (IHME), 2017.
	CSF Global (Bangladesh), International Centre for Eye Health (ICEH). Bangladesh - Barisal Rapid Assessment of Avoidable Blindness 2013.
	Associates for Community and Population Research (ACPR), National Institute of Population Research and Training (NIPORT). Bangladesh Utilization of Essential Service Delivery Survey 2013. Dhaka, Bangladesh: National Institute of Population Research and Training (NIPORT).
	Institute for Health Metrics and Evaluation (IHME), International Centre for Diarrhoeal Disease Research, Bangladesh (ICDDR,B), National Institute of Health Sciences (Sri Lanka), Research Institute for Tropical Medicine (Philippines), School of Population Health, University of Queensland (Australia). Improving Methods to Measure Comparable Mortality by Cause - PHMRC Shortened Questionnaire Open and Closed Response Sections 2013. Seattle, United States of America: Institute for Health Metrics and Evaluation (IHME), 2017.
	CDC Foundation, Centers for Disease Control and Prevention (CDC), National Center for Control of Rheumatic Fever and Heart Disease (Bangladesh), RTI International, World Health Organization (WHO). Bangladesh Global Youth Tobacco Survey 2013. Atlanta, United States of America: Centers for Disease Control and Prevention (CDC).
	Bangladesh Bureau of Statistics (BBS), International Labour Organization (ILO). Bangladesh Labor Force and Child Labor Force Survey 2013. Dhaka, Bangladesh: Bangladesh Bureau of Statistics (BBS).
	Gallup. Bangladesh World Poll 2013.
	ACNielsen Bangladesh, Directorate General of Health Services, Ministry of Health and Family Welfare (Bangladesh), United Nations Children's Fund (UNICEF), World Health Organization (WHO). Bangladesh EPI Coverage Evaluation Survey 2013.
	Bangladesh Bureau of Statistics (BBS), Government of Bangladesh, Ministry of Planning (Bangladesh), United Nations Children's Fund (UNICEF). Bangladesh Multiple Indicator Cluster Survey 2012-2013. New York, United States of America: United Nations Children's Fund (UNICEF), 2015.
	Bangladesh Bureau of Statistics (BBS), Ministry of Planning (Bangladesh). Bangladesh Health and Morbidity Status Survey 2012. Dhaka, Bangladesh: Bangladesh Bureau of Statistics (BBS).
	Gallup. Bangladesh World Poll 2012.

	CSF Global (Bangladesh), International Centre for Eye Health (ICEH). Bangladesh - Brahmanbaria and Satkhira Districts Rapid Assessment of Avoidable Blindness 2012.
	Center for Vaccine Development (Chile), Center for Vaccine Development, University of Maryland, Centers for Disease Control and Prevention (CDC), Department of Medical Microbiology and Immunology, Göteborg University, International Centre for Diarrhoeal Disease Research, Bangladesh (ICDDR,B), International Vaccine Institute, Perry Point Cooperative Studies Program Coordinating Center, U.S. Department of Veterans Affairs, School of Medicine, University of Virginia, University of Chile. Bangladesh - Mizrapur Global Enteric Multicenter Study 2011-2013. Baltimore, MD, United States: Center for Vaccine Development, University of Maryland.
	Global Alliance for Improved Nutrition (GAIN), Institute of Public Health Nutrition (Bangladesh), International Centre for Diarrhoeal Disease Research, Bangladesh (ICDDR,B), United Nations Children's Fund (UNICEF). Bangladesh National Micronutrients Status Survey 2011.
	Data Analysis and Technical Assistance Limited (Bangladesh), International Food Policy Research Institute (IFPRI), United States Agency for International Development (USAID). Bangladesh Integrated Household Survey 2011-2012. Washington D.C., United States of America: United States Agency for International Development (USAID).
	Directorate General of Health Services, Ministry of Health and Family Welfare (Bangladesh), Nielsen Company Bangladesh, United Nations Children's Fund (UNICEF), World Health Organization (WHO). Bangladesh EPI Coverage Evaluation Survey 2011.
	CSF Global (Bangladesh), International Centre for Eye Health (ICEH). Bangladesh - Tangail Rapid Assessment of Avoidable Blindness 2011. Grootebroek, Netherlands: RAAB Repository.
	Center for Social and Market Research (CSMR) (Bangladesh), Directorate General of Health Services, Ministry of Health and Family Welfare (Bangladesh), United Nations Children's Fund (UNICEF), World Health Organization (WHO). Bangladesh EPI Coverage Evaluation Survey 2014.
	ACNielsen Bangladesh, Directorate General of Health Services, Ministry of Health and Family Welfare (Bangladesh), United Nations Children's Fund (UNICEF), World Health Organization (WHO). Bangladesh EPI Coverage Evaluation Survey 2010.
	Fogarty International Center, National Institutes of Health (NIH), Foundation for the National Institutes of Health (FNIH), International Centre for Diarrhoeal Disease Research, Bangladesh (ICDDR,B). Bangladesh - Dhaka Malnutrition and Enteric Disease Study 2009-2014.

	Bangladesh Society of Medicine, Directorate General of Health Services, Ministry of Health and Family Welfare (Bangladesh), Ministry of Health and Family Welfare (Bangladesh), World Health Organization (WHO). Bangladesh STEPS Noncommunicable Disease Risk Factors Survey 2009-2010.
	Global Burden of Disease Collaborative Network. Disability Weights Measurement Study 2009-2010.
	Bangladesh Bureau of Statistics (BBS), CDC Foundation, Centers for Disease Control and Prevention (CDC), Ministry of Health and Family Welfare (Bangladesh), National Institute of Population Research and Training (NIPORT), National Institute of Preventive and Social Medicine, University of Dhaka (Bangladesh), World Health Organization (WHO). Bangladesh Global Adult Tobacco Survey 2009.
	Bangladesh Bureau of Statistics (BBS). Bangladesh Multiple Indicator Cluster Survey 2009. Dhaka, Bangladesh: Bangladesh Bureau of Statistics (BBS).
	Bangladesh Bureau of Statistics (BBS), Ministry of Planning (Bangladesh). Bangladesh Welfare Monitoring Survey 2009.
	International Tobacco Control Policy Evaluation Project, University of Dhaka, University of Waterloo (Canada). Bangladesh International Tobacco Control Survey 2009. Waterloo, Canada: International Tobacco Control Policy Evaluation Project.
	Gallup. Bangladesh World Poll 2009.
	ACNielsen Bangladesh, Directorate General of Health Services, Ministry of Health and Family Welfare (Bangladesh), United Nations Children's Fund (UNICEF), World Health Organization (WHO). Bangladesh EPI Coverage Evaluation Survey 2009.
	Mitra and Associates, United Nations Children's Fund (UNICEF), World Food Programme (WFP). Bangladesh Household Food Security and Nutrition Assessment 2008-2009.
	Gallup. Bangladesh World Poll 2008.
	ACNielsen Bangladesh, Directorate General of Health Services, Ministry of Health and Family Welfare (Bangladesh), United Nations Children's Fund (UNICEF), World Health Organization (WHO). Bangladesh EPI Coverage Evaluation Survey 2007-2008.
	Center for Vaccine Development (Chile), Center for Vaccine Development, University of Maryland, Centers for Disease Control and Prevention (CDC), Department of Medical Microbiology and Immunology, Göteborg University,

	International Centre for Diarrhoeal Disease Research, Bangladesh (ICDDR,B), International Vaccine Institute, Perry Point Cooperative Studies Program Coordinating Center, U.S. Department of Veterans Affairs, School of Medicine, University of Virginia, University of Chile. Bangladesh - Mizrapur Global Enteric Multicenter Study 2007-2011. Baltimore, MD, United States: Center for Vaccine Development, University of Maryland.
	Center for Vaccine Development (Chile), Center for Vaccine Development, University of Maryland, Centers for Disease Control and Prevention (CDC), Department of Medical Microbiology and Immunology, Göteborg University, International Centre for Diarrhoeal Disease Research, Bangladesh (ICDDR,B), International Vaccine Institute, Perry Point Cooperative Studies Program Coordinating Center, U.S. Department of Veterans Affairs, School of Medicine, University of Virginia, University of Chile. Bangladesh - Mizrapur GEMS 2007-2011 qPCR Diagnostic Results - University of Virginia.
	Macro International, Inc, Mitra and Associates, National Institute of Population Research and Training (NIPORT). Bangladesh Demographic and Health Survey 2007. Fairfax, United States of America: ICF International, 2009.
	BRAC, Damien Foundation, International Centre for Diarrhoeal Disease Research, Bangladesh (ICDDR,B), KNCV Tuberculosis Foundation, World Health Organization (WHO). Bangladesh Tuberculosis Disease-cum-Infection Prevalence Survey 2007-2009.
	Gallup. Bangladesh World Poll 2007.
	Centers for Disease Control and Prevention (CDC) and World Health Organization (WHO). Bangladesh Global Youth Tobacco Survey 2007. United States: Centers for Disease Control and Prevention (CDC), 2007.
	Centers for Disease Control and Prevention (CDC) and World Health Organization (WHO). Bangladesh - Dhaka Global Youth Tobacco Survey 2007. United States: Centers for Disease Control and Prevention (CDC), 2007.
	ACNielsen Bangladesh, Expanded Program on Immunization (Bangladesh), United Nations Children's Fund (UNICEF), World Health Organization (WHO). Bangladesh EPI Coverage Evaluation Survey 2006.
	Associates for Community and Population Research (ACPR), International Centre for Diarrhoeal Disease Research, Bangladesh (ICDDR,B), MEASURE Evaluation Project, Carolina Population Center, University of North Carolina, National Institute of Population Research and Training (NIPORT). Bangladesh Urban Health Survey 2006.
	Bangladesh Bureau of Statistics (BBS), Mitra and Associates, United Nations Children's Fund (UNICEF). Bangladesh Multiple Indicator Cluster Survey

	2006. New York, United States of America: United Nations Children's Fund (UNICEF).
	Gallup. Bangladesh World Poll 2005-2006.
	CSF Global (Bangladesh), International Centre for Eye Health (ICEH). Bangladesh - Satkhira District Rapid Assessment of Avoidable Blindness 2005. Grootebroek, Netherlands: RAAB Repository.
	Angeles, G; Lance, P, 2015, Bangladesh Urban NGO Service Delivery Program Evaluation Survey 2005. Bangladesh NSDP 2005 [RESTRICTED], https://hdl.handle.net/1902.29/11217, UNC Dataverse, V1
	Angeles, G; Lance, P, 2015, Bangladesh Rural NGO Service Delivery Program Evaluation Survey 2005. Bangladesh NSDP 2005 [RESTRICTED], https://hdl.handle.net/1902.29/11217, UNC Dataverse, V1
	BRAC, Population Council. Bangladesh Adolescent Survey 2005.
	Bangladesh Bureau of Statistics (BBS). Bangladesh Child and Mother Nutrition Survey 2005.
	Bangladesh Bureau of Statistics (BBS). Bangladesh Household Income and Expenditure Survey 2005. Dhaka, Bangladesh: Bangladesh Bureau of Statistics (BBS), 2007.
	ACNielsen, Directorate General of Health Services, Ministry of Health and Family Welfare (Bangladesh), United Nations Children's Fund (UNICEF), World Health Organization (WHO). Bangladesh EPI Coverage Evaluation Survey 2005.
	Institute of Public Health Nutrition (Bangladesh), International Centre for Diarrhoeal Disease Research, Bangladesh (ICDDR,B), National Institute of Population Research and Training (NIPORT). Bangladesh National Nutrition Programme Baseline Survey 2004-2005.
	Mitra and Associates, ORC Macro. Bangladesh Demographic and Health Survey 2004. Fairfax, United States of America: ICF International.
	ACNielsen Bangladesh, Expanded Program on Immunization (Bangladesh), United Nations Children's Fund (UNICEF), World Health Organization (WHO). Bangladesh EPI Coverage Evaluation Survey 2004.
	Centers for Disease Control and Prevention (CDC) and World Health Organization (WHO). Bangladesh - Dhaka Global Youth Tobacco Survey 2004. United States: Centers for Disease Control and Prevention (CDC), 2004.

	CARE Bangladesh, Data Analysis and Technical Assistance Limited (Bangladesh), International Food Policy Research Institute (IFPRI). Bangladesh - Dinajpur Supporting Household Activities for Health, Assets, and Revenue Survey, Round 3 2003. Washington, D.C., United States: International Food Policy Research Institute (IFPRI), 2009.
	MEASURE Evaluation Project, Carolina Population Center, University of North Carolina, Mitra and Associates. Bangladesh Urban NGO Service Delivery Program Evaluation Survey 2003.
	Associates for Community and Population Research (ACPR), MEASURE Evaluation Project, Carolina Population Center, University of North Carolina. Bangladesh Rural NGO Service Delivery Program Evaluation Survey 2003.
	International Food Policy Research Institute (IFPRI), 2009, "Bangladesh SHAHAR Dinajpur Baseline Survey, 2002-2003", https://hdl.handle.net/1902.1/17082, Harvard Dataverse, V1
	Ministry of Health and Family Welfare (Bangladesh), The Alliance for Safe Children (TASC), United Nations Children's Fund (UNICEF). Bangladesh Health and Injury Survey 2003.
	World Health Organization (WHO). Bangladesh World Health Survey 2003. Geneva, Switzerland: World Health Organization (WHO), 2005.
	Directorate General of Health Services, Ministry of Health and Family Welfare (Bangladesh), United Nations Children's Fund (UNICEF), World Health Organization (WHO). Bangladesh EPI Coverage Evaluation Survey 2003.
	Department of Biological Anthropology, University of Cambridge (UK), Institute of Epidemiology, Disease Control & Research (Bangladesh), National Center for Control of Rheumatic Fever & Heart Disease (Bangladesh), National Institute of Preventive and Social Medicine, University of Dhaka (Bangladesh), World Health Organization (WHO). Bangladesh STEPS Noncommunicable Disease Risk Factors Survey 2002.
	CARE Bangladesh, Data Analysis and Technical Assistance Limited (Bangladesh), International Food Policy Research Institute (IFPRI). Bangladesh - Dinajpur Supporting Household Activities for Health, Assets, and Revenue Survey, Round 1 2002. Washington, D.C., United States: International Food Policy Research Institute (IFPRI), 2009.
	Directorate General of Health Services, Ministry of Health and Family Welfare (Bangladesh), United Nations Children's Fund (UNICEF), World Health Organization (WHO). Bangladesh EPI Coverage Evaluation Survey 2002.
	Hamilton Health Sciences, McMaster University (Canada), Population Health Research Institute (PHRI). Bangladesh Prospective Urban and Rural Epidemiological Study.

	Associates for Community and Population Research (ACPR), International Centre for Diarrhoeal Disease Research, Bangladesh (ICDDR,B), Johns Hopkins University, Mitra and Associates, National Institute of Population Research and Training (NIPORT), ORC Macro. Bangladesh Special Demographic and Health Survey 2001. Fairfax, United States of America: ICF International.
	Directorate General of Health Services, Ministry of Health and Family Welfare (Bangladesh), United Nations Children's Fund (UNICEF), World Health Organization (WHO). Bangladesh EPI Coverage Evaluation Survey 2001.
	International Centre for Diarrhoeal Disease Research, Bangladesh (ICDDR,B), Naripokkho, Uppsala University, World Health Organization (WHO). Bangladesh WHO Multi-country Study on Women's Health and Domestic Violence Against Women 2001.
	Associates for Community and Population Research (ACPR), Bangladesh Bureau of Statistics (BBS), House of Survey Research (SURCH) (Bangladesh), United Nations Children's Fund (UNICEF). Bangladesh Multiple Indicator Cluster Survey 2000. Dhaka, Bangladesh: Bangladesh Bureau of Statistics (BBS).
	Directorate General of Health Services, Ministry of Health and Family Welfare (Bangladesh), United Nations Children's Fund (UNICEF), World Health Organization (WHO). Bangladesh EPI Coverage Evaluation Survey 2000.
	National Institute of Preventive and Social Medicine, University of Dhaka (Bangladesh), School of Public Health, Columbia University. Bangladesh - Araihazar Health Effects of Arsenic Longitudinal Study - Baseline 2000-2002.
	Macro Systems, Inc, Mitra and Associates, National Institute of Population Research and Training (NIPORT). Bangladesh Demographic and Health Survey 1999-2000. Fairfax, United States of America: ICF International.
	International Centre for Eye Health (ICEH), National Institute of Ophthalmology (Bangladesh). Bangladesh National Blindness and Low Vision Prevalence Survey 1999-2000.
	Macro International, Inc, Mitra and Associates, National Institute of Population Research and Training (NIPORT). Bangladesh Service Provision Assessment 1999-2000. Fairfax, United States of America: ICF International.
	Institute of Public Health Nutrition (Bangladesh). Bangladesh National Iodine Deficiency Disorders Survey 1999.
	ISSP Research Group (1999): International Social Survey Programme: Work Orientations II - ISSP 1997. GESIS Data Archive, Cologne. ZA3090 Data file Version 1.0.0, doi:10.4232/1.3090.

	Directorate General of Health Services, Ministry of Health and Family Welfare (Bangladesh), United Nations Children's Fund (UNICEF), World Health Organization (WHO). Bangladesh EPI Coverage Evaluation Survey 1998.
	Directorate General of Health Services, Ministry of Health and Family Welfare (Bangladesh), United Nations Children's Fund (UNICEF), World Health Organization (WHO). Bangladesh EPI Coverage Evaluation Survey 1997.
	Helen Keller International, Institute of Public Health Nutrition (Bangladesh), National Institute of Ophthalmology (Bangladesh), University of Dhaka. Bangladesh National Vitamin A Survey 1997-1998.
	International Centre for Diarrhoeal Disease Research, Bangladesh (ICDDR,B), Johns Hopkins School of Hygiene and Public Health, National Institute of Population Research and Training (NIPORT). Bangladesh Verbal Autopsy Study 1997.
	Macro International, Inc, Mitra and Associates, National Institute of Population Research and Training (NIPORT). Bangladesh Demographic and Health Survey 1996-1997. Fairfax, United States of America: ICF International.
	Bangladesh Bureau of Statistics (BBS), United Nations Children's Fund (UNICEF). Achieving the Mid-Decade Goals for Children in Bangladesh 1996.
	Bangladesh Bureau of Statistics (BBS), United Nations Children's Fund (UNICEF). Bangladesh Multiple Indicator Cluster Survey 1996.
	Bangladesh Bureau of Statistics (BBS). Bangladesh Smoking, Tobacco, and Health Survey 1995.
	Bangladesh Bureau of Statistics (BBS), United Nations Children's Fund (UNICEF). Bangladesh Child Nutrition Survey 1995-1996.
	Directorate General of Health Services, Ministry of Health and Family Welfare (Bangladesh), United Nations Children's Fund (UNICEF), World Health Organization (WHO). Bangladesh EPI Coverage Evaluation Survey 1995.
	Bangladesh Progress toward the Achievement of the Goals for the 1990's, 1995.
	Global Fund to Fight Aids Tuberculosis and Malaria (GFATM). Data Availability Among MARPs Prevalence and Population Size: 22 GF High Impact Countries and 5 Focus Countries.

	Bangladesh Bureau of Statistics (BBS), United Nations Children's Fund (UNICEF). Bangladesh Multiple Indicator Cluster Survey 1995.
	International Centre for Diarrhoeal Disease Research, Bangladesh (ICDDR,B), Johns Hopkins School of Hygiene and Public Health. Bangladesh Verbal Autopsy Study 1994-1995.
	Directorate General of Health Services, Ministry of Health and Family Welfare (Bangladesh), United Nations Children's Fund (UNICEF), World Health Organization (WHO). Bangladesh EPI Coverage Evaluation Survey 1994.
	Bangladesh Progress toward the Achievement of the Goals for the 1990's, 1994.
	Bangladesh Bureau of Statistics (BBS), United Nations Children's Fund (UNICEF). Bangladesh Multiple Indicator Cluster Survey 1994.
	Macro International, Inc, Mitra and Associates, National Institute of Population Research and Training (NIPORT). Bangladesh Demographic and Health Survey 1993-1994. Fairfax, United States of America: ICF International.
	Directorate General of Health Services, Ministry of Health and Family Welfare (Bangladesh), United Nations Children's Fund (UNICEF), World Health Organization (WHO). Bangladesh EPI Coverage Evaluation Survey 1993.
	International Council for the Control of Iodine Deficiency Disorders (ICCIDD), United Nations Children's Fund (UNICEF), University of Dhaka. Bangladesh National Iodine Deficiency Disorders Survey 1993.
	Bangladesh Bureau of Statistics (BBS), Institute of Statistical Research and Training, University of Dhaka (ISRT), United Nations Children's Fund (UNICEF), United Nations Statistics Division (UNSD). Bangladesh Multiple Indicator Cluster Survey 1993. New York, United States of America: United Nations Children's Fund (UNICEF).
	Bangladesh Bureau of Statistics (BBS). Bangladesh Child Nutrition Survey 1992.
	Directorate General of Health Services, Ministry of Health and Family Welfare (Bangladesh), United Nations Children's Fund (UNICEF), World Health Organization (WHO). Bangladesh EPI Coverage Evaluation Survey 1992.
	Mitra and Associates, National Institute of Population Research and Training (NIPORT), United States Agency for International Development (USAID). Bangladesh Contraceptive Prevalence Survey 1991.

	Directorate General of Health Services, Ministry of Health and Family Welfare (Bangladesh), United Nations Children's Fund (UNICEF), World Health Organization (WHO). Bangladesh EPI Coverage Evaluation Survey 1991.
Vital registration (10)	Bangladesh Bureau of Statistics (BBS). Bangladesh Sample Vital Registration System 2018. Dhaka, Bangladesh: Bangladesh Bureau of Statistics (BBS), 2019.
	Bangladesh Bureau of Statistics (BBS). Bangladesh Sample Vital Registration System 2017. Dhaka, Bangladesh: Bangladesh Bureau of Statistics (BBS), 2018.
	Bangladesh Bureau of Statistics (BBS). Bangladesh Sample Vital Registration System 2016. Dhaka, Bangladesh: Bangladesh Bureau of Statistics (BBS), 2017.
	Bangladesh Bureau of Statistics (BBS). Bangladesh Sample Vital Registration System 2015. Dhaka, Bangladesh: Bangladesh Bureau of Statistics (BBS), 2016.
	Bangladesh Bureau of Statistics (BBS). Bangladesh Sample Vital Registration System 2014. Dhaka, Bangladesh: Bangladesh Bureau of Statistics (BBS), 2015.
	Bangladesh Bureau of Statistics (BBS). Bangladesh Sample Vital Registration System 2013. Dhaka, Bangladesh: Bangladesh Bureau of Statistics (BBS), 2015.
	Bangladesh Bureau of Statistics (BBS). Bangladesh Sample Vital Registration System 2012. Dhaka, Bangladesh: Bangladesh Bureau of Statistics (BBS), 2014.
	Bangladesh Stillbirths Estimates 2011-2013.
	Bangladesh Bureau of Statistics (BBS). Bangladesh Sample Vital Registration System 2010.
	Bangladesh Bureau of Statistics (BBS). Bangladesh Sample Vital Registration System 2003. Dhaka, Bangladesh: Bangladesh Bureau of Statistics (BBS), 2006.

Table S13. Bangladesh current healthcare expenditure in 2020

(Source: Health expenditure series. Geneva: World Health Organization 2023, available at: http://apps.who.int/nha/database/DataExplorerRegime.aspx)3

Indicators	Value (Percentage)
Current Health Expenditure (CHE) as % Gross Domestic Product (GDP)	3
Current Health Expenditure (CHE) per Capita in US$	51
Current Health Expenditure (CHE) % Gross domestic product (GDP)	3
Domestic General Government Health Expenditure (GGHE-D) % Gross domestic product (GDP)	0
Domestic Private Health Expenditure (PVT-D) % Gross domestic product (GDP)	2
External Health Expenditure (EXT) % Gross domestic product (GDP)	0
Domestic General Government Health Expenditure (GGHE-D) as % Current Health Expenditure (CHE)	18
Domestic Private Health Expenditure (PVT-D) as % Current Health Expenditure (CHE)	77
Out-of-pocket (OOPS) as % of Current Health Expenditure (CHE)	74
Out-of-Pocket Expenditure (OOPS) per Capita in US$	37
External Health Expenditure (EXT) per Capita in US$	3

N.B.: WHO regional, income-group and global aggregates are calculated using absolute amounts in national currency units converted to Purchasing Power Parity (PPP) equivalents. Values smaller than 0.05% may appear as zero. For per capita indicators, when the value is less than 0.5 it is represented as < 1. Absolute values of expenditures are expressed in nominal terms (current prices). National currency unit per US$ are calculated using the average exchange rates for the year. Purchasing power parity (PPP); international dollar (Int $) values have been revised based on the World Bank's 2011 International Comparison Program (ICP). 3

Table S14. Health expenditure and donor assistance for health in Bangladesh and comparator countries in 2020

(Source: Health expenditure series. Geneva: World Health Organization 2023, available at: http://apps.who.int/nha/database/DataExplorerRegime.aspx)3

Countries	Indicators	2020 Value
**Pakistan**	Current Health Expenditure (CHE) in million US$	8,674
	Current Health Expenditure (CHE) per capita US$	38
	Domestic General Government Health Expenditure (GGHE-D) in million US$	3,054
	Domestic Private Health Expenditure (PVT-D) in million US$	5,073
	External Health Expenditure (EXT) in million US$	**547**
	External Health Expenditure (EXT) in % of Current Health Expenditure (CHE)	**6**
**Bangladesh**	Current Health Expenditure (CHE) in million US$	8,482
	Current Health Expenditure (CHE) per capita US$	51
	Domestic General Government Health Expenditure (GGHE-D) in million US$	1,531
	Domestic Private Health Expenditure (PVT-D) in million US$	6,493
	External Health Expenditure (EXT) in million US$	**459**
	External Health Expenditure (EXT) in % of Current Health Expenditure (CHE)	**5**
**Bhutan**	Current Health Expenditure (CHE) in million US$	103
	Current Health Expenditure (CHE) per capita US$	134
	Domestic General Government Health Expenditure (GGHE-D) in million US$	81
	Domestic Private Health Expenditure (PVT-D) in million US$	17
	External Health Expenditure (EXT) in million US$	**5**
	External Health Expenditure (EXT) in % of Current Health Expenditure (CHE)	**5**
**India**	Current Health Expenditure (CHE) in million US$	79,076
	Current Health Expenditure (CHE) per capita US$	57
	Domestic General Government Health Expenditure (GGHE-D) in million US$	28,980
	Domestic Private Health Expenditure (PVT-D) in million US$	49,315
	External Health Expenditure (EXT) in million US$	**781**
	External Health Expenditure (EXT) in % of Current Health Expenditure (CHE)	**1**
**Nepal**	Current Health Expenditure (CHE) in million US$	1,711
	Current Health Expenditure (CHE) per capita US$	58
	Domestic General Government Health Expenditure (GGHE-D) in million US$	514
	Domestic Private Health Expenditure (PVT-D) in million US$	1,016
	External Health Expenditure (EXT) in million US$	**181**
	External Health Expenditure (EXT) in % of Current Health Expenditure (CHE)	**11**

Section 6. References

1. Vos T, Lim SS, Abbafati C, et al. Global burden of 369 diseases and injuries in 204 countries and territories, 1990–2019: a systematic analysis for the Global Burden of Disease Study 2019. *The Lancet* 2020; **396**(10258): 1204-22.

2. Stevens GA, Alkema L, Black RE, et al. Guidelines for accurate and transparent health estimates reporting: the GATHER statement. *The Lancet* 2016; **388**(10062): e19-e23.

3. World Health Organization. http://apps.who.int/nha/database/DataExplorerRegime.aspx. 2023 (accessed 22/07/2023 2023).

Section 7. Author's Contributions

Providing data or critical feedback on data sources

Sheikh Mohammad Alif, Palash Chandra Banik, Fazle Rabbi Chowdhury, Rajat Das Gupta, Syed Emdadul Haque, Simon I Hay, Sheikh Jamal Hossain, Sheikh Mohammed Shariful Islam, M Nuruzzaman Khan, Mohammed A Mamun, Ali H Mokdad, Mohammad Ali Moni, Christopher J L Murray, Mohsen Naghavi, Mahfuzar Rahman, Zubair Ahmed Ratan, Rezaul Karim Ripon, and Riaz Uddin.

Developing methods or computational machinery

Sheikh Mohammed Shariful Islam, Sheikh Mohammad Alif, Palash Chandra Banik, Simon I Hay, M Nuruzzaman Khan, Ali H Mokdad, Mohammad Ali Moni, Christopher J L Murray, Mohsen Naghavi, KM Saif-Ur-Rahman, and Sojib Bin Zaman.

Providing critical feedback on methods or results

Md. Abdul Alim, Ripon Kumar Adhikary, Syed Masud Ahmed, Sheikh Mohammad Alif, Afifa Anjum, Palash Chandra Banik, Rahat I. Chowdhury, Sohel Reza Choudhury, Subasish Das, Md Omar Faruk, Rajat Das Gupta, Md Abdul Hannan, Md Nuruzzaman Haque, Syed Emdadul Haque, M Tasdik Hasan, Simon I Hay, Md Belal Hossain, Md Mahbub Hossain, Muttaquina Hossain, Sahadat Hossain, Sheikh Jamal Hossain, Md. Tanvir Hossen, Sheikh Mohammed Shariful Islam, M Nuruzzaman Khan, Md Jobair Khan, Katherine M Livingstone, Ralph Maddison, Mohammed A Mamun, Ali H Mokdad, Mohammad Ali Moni, Christopher J L Murray, Mohsen Naghavi, Mahfuzar Rahman, Md Mosfequr Rahman, Mosiur Rahman, Zubair Ahmed Ratan, Md. Marufur Rahman, Rezaul Karim Ripon, KM Saif-Ur-Rahman, Abu Sayeed, Md Shahjahan Siraj, Saima Sultana, Riaz Uddin, and Sojib Bin Zaman.

Drafting the work or revising it critically for important intellectual content

Md. Abdul Alim, Syed Imran Ahmed, Syed Masud Ahmed, Sheikh Mohammad Alif, Afifa Anjum, Palash Chandra Banik, Rahat I. Chowdhury, Fazle Rabbi Chowdhury, Sohel Reza Choudhury, Subasish Das, Md Omar Faruk, Rajat Das Gupta, Md Abdul Hannan, M Tasdik Hasan, Simon I Hay, Md Belal Hossain, Md Mahbub Hossain, Muttaquina Hossain, Sahadat Hossain, Md Tanvir Hossen, Sheikh Mohammed Shariful Islam, M Nuruzzaman Khan, Md Jobair Khan, Katherine M Livingstone, Ralph Maddison, Max L Mehlman, Ali H Mokdad, Mohammad Ali Moni, Christopher J L Murray, Mohsen Naghavi, Mahfuzar Rahman, Md Mosfequr Rahman, Zubair Ahmed Ratan, Md. Marufur Rahman, Rezaul Karim Ripon, KM Saif-Ur-Rahman, Malabika Sarker, Abu Sayeed, George Siopis, Saima Sultana, Riaz Uddin, and Sojib Bin Zaman.

Managing the estimation or publications process

Sheikh Mohammed Shariful Islam, Simon I Hay, Max L Mehlman, Ali H Mokdad, Christopher J L Murray, Mohsen Naghavi, and Riaz Uddin.
